# Bacterial nasopharyngeal colonisation in children in South Africa before and during the COVID-19 pandemic: an observational study

**DOI:** 10.1016/S2666-5247(23)00260-4

**Published:** 2024-01

**Authors:** Courtney P Olwagen, Sarah L Downs, Alane Izu, Lebohang Tharasimbi, Lara Van Der Merwe, Marta C Nunes, Shabir A Madhi

**Affiliations:** a South Africa Medical Research Council Vaccines and Infectious Diseases Analytics Research Unit, University of the Witwatersrand, Faculty of Health Science, Johannesburg, South Africa; b Department of Science–National Research Foundation, Vaccine Preventable Diseases, University of the Witwatersrand, Faculty of Health Science, Johannesburg, South Africa; c Wits Infectious Diseases and Oncology Research Institute, University of the Witwatersrand, Faculty of Health Science, Johannesburg, South Africa; d Centre of Excellence in Respiratory Pathogens, Hospices Civils de Lyon, and Centre International de Recherche en Infectiologie, Inserm, U1111, Université Claude Bernard Lyon 1, CNRS, UMR5308, Lyon, France

## Abstract

**Background:**

Deployment of non-pharmaceutical interventions such as face masking and physical distancing during the COVID-19 pandemic could have altered the transmission dynamics and carriage of respiratory organisms. We evaluated colonisation with *Streptococcus pneumoniae* and other upper respiratory tract bacterial colonisers before and during the COVID-19 pandemic.

**Methods:**

We did two cross-sectional surveys in Soweto, South Africa from July 3 to Dec 13, 2018 (pre-COVID-19 period) and from Aug 4, 2021, to March 31, 2022 (COVID-19 period) in healthy children (aged ≤60 months) who had recorded HIV status and had not received antibiotics in the 21 days before enrolment. At enrolment, we collected nasopharyngeal swab samples from child participants. Following nucleic acid extraction, nanofluidic quantitative PCR was used to screen all samples for 92 *S pneumoniae* serotypes and 14 other bacteria. The primary objective was to compare the prevalence and density of pneumococcal nasopharyngeal colonisation, overall and stratified by 13-valent pneumococcal conjugate vaccine (PCV13) serotypes and non-vaccine serotypes. Secondary study objectives included a comparison of serotype-specific pneumococcal colonisation and density, as well as colonisation by the 14 other bacteria in the COVID-19 versus pre-COVID-19 period. We used an adjusted multiple logistic and linear regression model to compare the colonisation prevalence and density between study periods.

**Findings:**

We analysed nasopharyngeal swabs from 1107 children (n=571 in the pre-COVID-19 period; n=536 in the COVID-19 period). We observed no change in overall pneumococcal colonisation between periods (274 [51%] of 536 in the COVID-19 period *vs* 282 [49%] of 571 in the pre-COVID-19 period; adjusted odds ratio [aOR] 1·03 [95% CI 0·95–1·12]). The prevalence of PCV13 serotypes was lower in the COVID-19 than in the pre-COVID-19 period (72 [13%] *vs* 106 [19%]; 0·87 [0·78–0·97]), whereas the prevalence of non-typeable *S pneumoniae* was higher (34 [6%] *vs* 63 [12%]; 1·30 [1·12–1·50]). The mean log_10_ density for overall pneumococcal colonisation was lower in the COVID-19 period than in the pre-COVID-19 period (3·96 [95% CI 3·85–4·07] *vs* 4·72 [4·63–4·80] log_10_ genome equivalents per mL; p<0·0001). A lower density of non-vaccine serotypes (3·63 [3·51–3·74] *vs* 4·08 [3·95–4·22] log_10_ genome equivalents per mL; p<0·0001) and non-typeable *S pneumoniae* (3·11 [2·94–3·29] *vs* 4·41 [4·06–4·75] log_10_ genome equivalents per mL; p<0·00001) was also observed in the COVID-19 period. There was no difference in the density of PCV13 serotypes between the periods. The prevalence of colonisation during the COVID-19 versus pre-COVID-19 period was lower for non-typeable *Haemophilus influenzae* (280 [49%] *vs* 165 [31%]; aOR 0·77 [95% CI 0·71–0·84]), *Moraxella catarrhalis* (328 [57%] *vs* 242 [45%]; 0·85 [0·79–0·92]), and *Neisseria lactamica* (51 [9%] *vs* 13 [2%]; 0·64 [0·52–0·78]), but higher for *Acinetobacter baumannii* (34 [6%] *vs* 102 [19%]; 1·55 [1·35–1·77]) and *Staphylococcus aureus* (29 [5%] *vs* 52 [10%]; 1·28 [1·10–1·50]).

**Interpretation:**

There were variable effects on the colonisation prevalence and density of bacterial organisms during the COVID-19 compared with the pre-COVID-19 period. The lower prevalence of PCV13 serotype together with other respiratory organisms including non-typeable *H influenzae* and *M catarrhalis* could have in part contributed to a decrease in all-cause lower respiratory tract infections observed in South Africa during the initial stage of the COVID-19 pandemic. The pathophysiological mechanism for the increase in *A baumannii* and *S aureus* colonisation warrants further investigation, as does the clinical relevance of these findings.

**Funding:**

The Bill & Melinda Gates Foundation.

## Introduction

Pneumococcal conjugate vaccines (PCVs) have been introduced in the childhood immunisation programmes of most countries, which has led to a reduction in vaccine-serotype nasopharyngeal colonisation and more than 80% reduction in vaccine-serotype invasive pneumococcal disease globally, including in South Africa.^1^ Nevertheless, *Streptococcus pneumoniae* remains a leading cause of morbidity and mortality worldwide, with the highest burden in children younger than 5 years living in sub-Saharan Africa.^1^

**Research in context**

**Evidence before this study**

The association between the COVID-19 pandemic and the circulation of respiratory organisms in children during the time that public health measures aimed at reducing the transmission of the SARS-CoV-2 virus were in place, is unclear. Most studies have been undertaken in adults and have focused on the effect of invasive disease or on SARS-CoV-2 co-infections. To investigate the effect that non-pharmaceutical interventions imposed during the COVID-19 pandemic had on bacterial colonisation in children, we did a literature search using PubMed to identify studies from database inception to May 16, 2023, using the search terms “pandemic” or “COVID-19” or “SARS-CoV-2” and “carriage” or “colonisation” or “colonization” and “children” and “non-pharmaceutical interventions” or “NPI” or “containment”. Notably, we found few data were available, with only six studies undertaken in Belgium, Indonesia, Israel, Serbia, and the USA (in the states of Texas and New York). All of the studies were limited in that they only tested for one to four bacterial organisms and only three of the studies (from Israel, Serbia, and Texas) reported on the overall colonisation prevalence of *Streptococcus pneumoniae* before and during the COVID-19 pandemic, although the studies done in Israel and Serbia did not report on serotype-specific changes.

**Added value of this study**

Our study is the first to do an in-depth investigation of the colonisation of 92 pneumococcal serotypes together with 14 other bacteria in children (aged ≤60 months) before and during the COVID-19 pandemic. Our study provides insights into the complex and dynamic relationships between co-circulating respiratory organisms during the COVID-19 pandemic. The lower prevalence of colonisation with 13-valent pneumococcal conjugate vaccine serotypes together with other respiratory organisms including non-typeable *Haemophilus influenzae* and *Moraxella catarrhalis* could have, in part, contributed to the decrease in all-cause lower respiratory tract infections observed in South Africa during the initial stage of the COVID-19 pandemic. Furthermore, the study identified a 3·2-fold increase in the prevalence of *Acinetobacter baumannii* colonisation and a 2-fold increase in the prevalence of *Staphylococcus aureus* colonisation in the COVID-19 compared with the pre-COVID-19 period. These organisms form part of the highly virulent ESKAPE pathogens (ie, *Enterococcus faecium, S aureus, Klebsiella pneumoniae, Acinetobacter baumannii, Pseudomonas aeruginosa, and Enterobacter* spp), which are often associated with multidrug resistant infections in South Africa.

**Implications of all the available evidence**

We detected changes in the colonisation prevalence and density across multiple respiratory organisms including *S pneumoniae* and *A baumannii* in young children living in South Africa during the COVID-19 pandemic compared with the pre-COVID-19 period. The study provides an early caution for the risk of transmission and disease from *A baumannii* and *S aureus*, the pathophysiological mechanism and biological relevance of which warrant further investigation.

In response to the COVID-19 pandemic in early 2020, most countries implemented a range of stringent non-pharmaceutical interventions aimed at limiting infections by SARS-CoV-2.^2^ The COVID-19 responses might have inadvertently led to the interruption of surveillance of bacterial diseases and immunisation programmes in many settings.^3^ Between Jan 1, 2018, and May, 31, 2020, the Invasive Respiratory Infections Surveillance Project in 26 countries, including South Africa, reported reductions in invasive bacterial disease from *S pneumoniae*, *Haemophilus influenzae*, and *Neisseria meningitidis* during the early months of the COVID-19 pandemic, which was attributed to a decrease in transmission events.^4^ However, the effect that SARS-CoV-2 infections and public health interventions during the COVID-19 pandemic had on common bacterial colonisers of the upper respiratory tract—ie, pneumococcus, non-typeable *H influenzae*, and *Moraxella catarrhalis*—remains unclear. Published studies on the effect of COVID-19 and non-pharmaceutical interventions have focused on evaluating bacterial carriage or secondary bacterial infections in people infected with SARS-CoV-2,^5^, ^6^ but not at the population level.

We aimed to evaluate serotype-specific pneumococcal colonisation, together with colonisation by other bacteria, including *H influenzae* type-b*,* non-typeable *H influenzae, M catarrhalis*, *Klebsiella pneumoniae,* and *Acinetobacter baumannii,* during the COVID-19 pandemic compared with the pre-pandemic period in South African children aged 5 years or younger.

## Methods

### Study population

Two prospective cross-sectional surveys were undertaken in Soweto, a low–middle-income settlement near Johannesburg, Gauteng province, South Africa from July 3 to Dec 13, 2018, (pre-COVID-19 period) and from Aug 4, 2021, to March 31, 2022 (COVID-19 period). The 2018 survey was undertaken to evaluate the prevalence of serotype-specific *S pneumoniae* and other bacterial nasopharyngeal colonisation 8 years after the introduction of the 13-valent PCV (PCV13) into the South African childhood public immunisation programme, and has been reported on previously.^7^ The 2021 survey was undertaken to compare the prevalence of serotype-specific *S pneumoniae* and other bacterial nasopharyngeal colonisation during the COVID-19 pandemic with the pre-COVID-19 period. In both surveys, children were enrolled through household-level surveys and were selected using the WHO Expanded Programme on Immunisation survey method that involved two-stage, systematic, random sampling of dwelling units, within enumeration areas with probability proportional to the cluster or enumeration area size, as detailed in the appendix (p 2).

Inclusion criteria were written informed consent from a parent or legal guardian (aged ≥18 years), child being aged 60 months or younger, and recorded HIV-exposure status. Exclusion criteria were symptoms of febrile illness (fever ≥37·5°C), having received antibiotics during the 21 days before sample collection (except for prophylactic use of cotrimoxazole in infants born to women living with HIV), and the primary caregiver being unwilling to undergo an HIV rapid test or to provide up-to-date information on HIV status (documented test results within 6 months of sampling date).

Since 2009, children receive PCV using a 2 + 1 schedule (age 6, 14, and 40 weeks) as part of the South African public immunisation programme, with transition from the PCV7 to PCV13 taking place in May, 2011. Details of other childhood vaccines administered are detailed in the appendix (p 2).

Some households might have been randomly selected in both surveys; however, due to the interval between study periods, enrolled children would not have contributed to the same age strata in the pre-COVID-19 and COVID-19 periods. During the COVID-19 pandemic, the government imposed societal restrictions in a five-tiered level approach in South Africa, with level 5 being the strictest lockdown with a stay-at-home order and both international and provincial travel not permitted. Details of the societal restrictions are in the appendix (pp 3–5). When enrolment into the COVID-19 period survey began (Aug 4, 2021), South Africa had endured three waves of COVID-19 and enrolments were conducted during adjusted restriction levels 3, 2, and 1.

The study was approved by the Human Research Ethics Committee of the University of Witwatersrand (M170314 and M200421). Written informed consent was obtained from the parents or legal guardians, including on behalf of their children.

### Procedures

Nasopharyngeal flocked swab (FLOQSwabsTM, Copan Diagnostics, Murrieta, CA, USA) samples were collected from all eligible children within selected households and placed in 1·5 mL of skim milk–tryptone–glucose–glycerol medium (prepared in house). Samples were kept on ice for a maximum of 6 h until transported to Wits-VIDA Research Unit (Chris Hani Baragwanath Hospital, Soweto, South Africa) for storage at –70°C, according to WHO recommendations. At the time of swab collection, information on demographics and risk factors for colonisation were collected, as detailed in the appendix (p 2). Children were only sampled at the time of enrolment with no follow-up visits.

Nucleic acids were extracted automatically from the nasopharyngeal flocked swabs using the NucliSens easyMAG extraction system (BioMérieux, Marcy l'Etoile, France) following the manufacturer's instructions. All extracts underwent nanofluidic quantitative PCR testing on the Biomark HD System (Standard BioTools formerly known as Fluidigm; San Francisco, CA, USA) as previously described^8^ and were tested for 15 bacterial species (*S pneumoniae, H influenzae, M catarrhalis, Neisseria lactamica, N meningitidis, Staphylococcus aureus, Streptococcus pyogenes, Bordetella pertussis, Bordetella holmesii, Bordetella bronchiseptica, Bordetella parapertussis, Klebsiella pneumoniae, Acinetobacter baumannii,* and *Streptococcus oralis*) and 92 pneumococcal serotypes (appendix p 6). Extracts from the COVID-19 period were also tested for 13 respiratory viruses (human parainfluenza virus 1 [PIV1]; PIV3; respiratory syncytial virus [RSV] A and B; human metapneumovirus; influenza virus A and B; rhinovirus; seasonal coronaviruses OC43, NL63, 229E, and HKU1; and SARS-CoV-2) and two additional pneumococcal serotypes (19F-atypical and 19C) that were optimised and validated using a previously described approach^8^ with some modifications (appendix pp 2–3, 7–12). The quantification cycle values were converted to density (log_10_ genome equivalents per mL) and log_10_-transformed. Relevant, previously described algorithms^8^ were applied to interpret pneumococcal serotypes and bacterial and viral targets. Samples were considered positive for a target if the serotype-specific density was above the limit of detection for that assay (appendix pp 13–15)^9^ and at least three out of the four pneumococcal reference genes (*lytA, piaB, ply,* and *Xisco*) were detected. Non-typeable *S pneumoniae* was defined as pneumococcal-positive samples where no serotype was identified or where the difference in the average density of quantitative pneumococcal reference genes (*lytA* and *piaB*) and the sum of all detected serotype-specific densities were outside of the upper limit of the 95% CI when compared using Bland Altman plots—ie, when non-typeable *S pneumoniae* was co-carried with a typeable serotype (appendix p 44).

### Outcomes

The primary study objective was to evaluate the effect of the COVID-19 pandemic on the colonisation prevalence and density of overall pneumococcus and of combined PCV13 serotypes and combined non-vaccine serotypes. The secondary study objective was to evaluate the prevalence and density of individual pneumococcal serotypes and of other bacteria before and during the COVID-19 pandemic. Children enrolled in the COVID-19 period were also tested for 13 respiratory viruses and we report on the prevalence and density of these viruses as exploratory analyses.

### Statistical analysis

Statistical analysis was performed with Stata version 11.0. A minimum sample size of 408 was adequate to detect 80% change in the overall colonisation with PCV13 serotypes between the study groups assuming a two-sided significance level of 0·05 and 80% power. To determine immunisation coverage, children were stratified into three groups according to the PCV immunisation schedule used in South Africa (ie, 2 + 1 PCV13 schedule administered at ages 6, 14, and 40 weeks) and their eligibility for vaccination (ie, ≥6 to <14 weeks, ≥14 to <40 weeks, and ≥40 weeks). Student's *t* test or Pearson's χ^2^ test was used to compare demographic characteristics and risk factors for colonisation between the study periods. A multiple logistic and linear regression model adjusted for breastfeeding status, gender, HIV infection, age at sample collection, cotrimoxazole prophylaxis, and tuberculosis treatment was used to compare the colonisation prevalence and density, respectively, between the study periods. Only records with complete information for all covariates were included in the model, with two participants excluded from the pre-COVID-19 group during adjusted analysis. The prevalence of colonisation is reported along with adjusted odds ratios (aORs). To summarise the density of each organism, the mean log_10_ density is reported. All statistics are reported alongside 95% CIs. To determine the ranking of multiple colonisers, each serotype, bacterium, or virus was ranked according to its relative carriage density. Single colonisers were included in the analysis as dominant colonising serotypes, bacteria, or viruses. p values of 0·05 or less were considered significant. All analyses are exploratory and no adjustment for multiple comparisons was made.

### Role of the funding source

The funder of the study had no role in study design, data collection, data analysis, data interpretation, or writing of the report.

## Results

**Figure 1:**
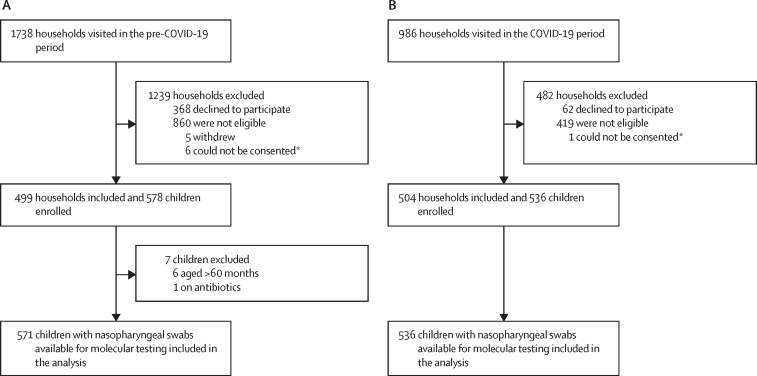
Study profiles for the cross-sectional surveys done in the pre-COVID-19 (A) and COVID-19 (B) periods *Parent, or guardian, was not present or able to give consent for inclusion into the study.

Nanofluidic quantitative PCR analysis was undertaken on 1107 nasopharyngeal flocked swabs collected from 1107 children (571 in the pre-COVID-19 period and 536 in the COVID-19 period; figure 1). The mean age at the time of sample collection did not differ between the periods (table 1). Although there was no difference in the proportion of children living with HIV, the frequency of children taking cotrimoxazole prophylaxis was higher in the COVID-19 period (18 [3%] of 536) compared with the pre-COVID-19 period (two [<1%] of 571; p<0·0001). Immunisation with three doses of PCV for age-eligible children (ie, ≥40 weeks) was 93% during both periods, and 555 (99%) of 560 children during the pre-COVID-19 and 514 (98%) of 526 during the COVID-19 period had received at least one dose of PCV (appendix p 16).

**Table 1:** Baseline characteristics

		**Covid-19 period (n=571)**	**Pre-COVID-19 period (n=536)**	p value*
Age, years				
All children		2·2 (1·3)	2·3 (1·5)	0·25
Children aged 24 months or younger		1·0 (0·5, n=289)	1·1 (0·5, n=267)	0·41
Children older than 24 months		3·3 (0·8, n=282)	3·4 (0·9, n=269)	0·12
Gender		..	..	0·51
	Male	281 (49%)	275 (51%)	..
	Female	289 (51%)	261 (49%)	..
	Unknown	1 (<1%)	0	..
Race		..	..	0·59
	Black	569 (100%)	534 (100%)	..
	Mixed	1 (<1%)	2 (<1%)	..
	Unknown	1 (<1%)	0	..
Currently breastfed		137 (24%)	133 (25%)	0·75
Ever breastfed		454 (80%)	408 (76%)	0·15
Attendance at day-care		115 (20%)	102 (19%)	0·64
Children living with HIV		3 (1%)	2 (<1%)	0·71
Prophylaxis cotrimoxazole treatment		2 (<1%)	18 (3%)	<0·0001
Treated for tuberculosis in the past year		2/570 (<1%)	4/536 (1%)	0·37
Currently taking antibiotics		1 (<1%)	0	0·33
Hospitalised in last 3 months		0	3 (1%)	0·073

Data are mean (SD), n (%), or n/N (%).

* Student's *t* test or Pearson χ^2^ test was used to compare demographic and risk factors for colonisation between the cohorts.

Although there was no difference in the overall pneumococcal colonisation prevalence between the study periods, the prevalence of PCV13 serotypes was lower in the COVID-19 period than in the pre-COVID-19 period (table 2; appendix p 17). The prevalence of overall non-vaccine serotype colonisation did not differ between the study periods; however, there were 1·30 times higher odds of colonisation by non-typeable *S pneumoniae* in the COVID-19 period than in the pre-COVID-19 period (63 [12%] of 536 *vs* 34 [6%] of 571, 95% CI 1·12–1·50; table 2; appendix pp 17). The serotype-specific prevalences are shown in the appendix (pp 18–27). Briefly, the colonisation prevalence of individual PCV13 serotypes remained similar between the periods, except for a lower prevalence of serotype 19A (2% [95% CI 1–3], nine of 571 *vs* 0 [0–1%]; p=0·0039) and serotype 5 (2% [1–4], 13 of 571 *vs* 1% [0–2], five of 536; p=0·043) in the COVID-19 period.

**Table 2:** Prevalence of *Streptococcus pneumoniae* colonisation

	**Pre-COVID-19 period, n (% [95% CI])**	**COVID-19 period, n (% [95% CI])**	**OR (95% CI); p value**	**aOR (95% CI); p value**
**All children (pre-COVID-19, n=571; COVID-19 n=536)**				
Overall pneumococcus	282 (49% [45–54])	274 (51% [47–55])	1·07 (0·84–1·30); p=0·58	1·03 (0·95–1·12); p=0·42
Non-PCV13 serotypes	216 (38% [34–42])	222 (41% [37–46])	1·16 (0·91–1·47); p=0·22	1·07 (0·98–1·16); p=0·12
PCV13 serotypes	106 (19% [16–22])	72 (13% [2–17])	0·68 (0·49–0·94); p=0·021	0·87 (0·78–0·97); p=0·014
Non-typeable*S pneumoniae*	34 (6% [4–8])	63 (12% [9–15])	2·10 (1·36–3·25); p=0·0010	1·30 (1·12–1·50); p=0·0005
**Children aged 24 months or younger (pre-COVID-19, n=289; COVID-19 n=267)**				
Overall pneumococcus	147 (51% [45–57])	135 (50% [46–58])	0·98 (0·70–1·37); p=0·94	1–00 (0·89–1·12); p=0·99
Non-PCV13 serotypes	111 (38% [33–44])	105 (39% [34–45])	1·04 (0·74–1·46); p=0·82	1·03 (0·91–1·15); p=0·66
PCV13 serotypes	57 (20% [15–25])	38 (14% [10–19])	0·67 (0·42–1·05); p=0·08	0·86 (0·74–1–00); p=0·056
Non-typeable*S pneumoniae*	22 (7% [5–11])	31 (12% [8–16])	1·59 (0·90–2·83); p=0·11	1·19 (0·98–1·44); p=0·076
**Children older than 24 months (pre-COVID-19, n=282; COVID-19 n=269)**				
Overall pneumococcus	135 (48% [42–54])	139 (52% [44–56])	1·16 (0·82–1·65); p=0·395	1·07 (0·95–1·20); p=0·25
Non-PCV13 serotypes	105 (37% [32–43])	117 (44% [38–50])	1·30 (0·91–1·85); p=0·14	1·11 (0·99–1·24); p=0·086
PCV13 serotypes	49 (17% [13–22])	34 (13% [9–17])	0·69 (0·41–1·13); p=0·124	0·89 (0·76–1·04); p=0·14
Non-typeable*S pneumoniae*	12 (4% [3–7])	32 (12% [9–16])	3·03 (1·48–6·62); p=0·0014	1·46 (1·16–1·84); p=0·0013

Data are PCV13 serotypes including serotypes or serogroups: 1, 3, 4, 5, 6A, 6B, 7A/F, 9A/V, 14, 18B/C, 19A, 19F, and 23F. aOR=adjusted OR. OR=odds ratio. PCV13=13-valent pneumococcal conjugate vaccine. aORs and 95% CI were calculated using logistic regression analyses.

There was no difference in the overall pneumococcal colonisation prevalence and non-vaccine serotype colonisation between the periods in children aged 24 months or younger and those older than 24 months; however, the prevalence of PCV13 serotypes was 1·4-fold lower in the COVID-19 compared with the pre-COVID-19 periods for both children aged 24 months or younger and those older than 24 months, although this difference was not significant (table 2). The prevalence of non-typeable *S pneumoniae* was significantly higher in the COVID-19 period (32 [12%] of 269) compared with the pre-COVID-19 period (12 [4%] of 282, aOR 1·46 [95% CI 1·16–1·84]) in children older than 24 months; no significant difference was observed in children aged 24 months or younger (31 [12%] of 267 *vs* 22 [8%] of 289; aOR 1·19 [95% CI 0·98–1·44]; table 2).

**Figure 2:**
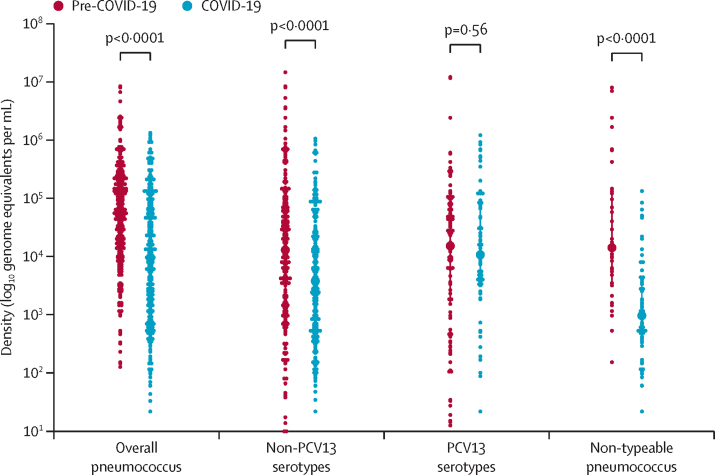
Mean log_10_ density (genome equivalents per mL) of *Streptococcus pneumoniae* in children aged 0–60 months Overall includes all pneumococci. Non-PCV13 serotypes are all serotypes or serogroups not included in PCV13. PCV13 serotypes are serotypes or serogroups 1, 3, 4, 5, 6A, 6B, 7A/F, 9A/V, 14, 18B/C, 19A, 19F, and 23F. PCV13=13-valent pneumococcal conjugate vaccine.

The density for overall pneumococcal colonisation was lower in the COVID-19 period (3·96 log_10_ genome equivalents per mL [95% CI 3·85–4·07]) than in the pre-COVID-19 period (4·72 log_10_ genome equivalents per mL [4·63–4·80]; p<0·0001). The lower density of pneumococcal colonisation in the COVID-19 period was driven by lower densities of non-vaccine serotypes (3·63 [3·51–3·74] *vs* 4·08 [3·95–4·22] log_10_ genome equivalents per mL; p<0·0001) and non-typeable *S pneumoniae* (3·11 [2·94–3·29] *vs* 4·41 [4·06–4·75] log_10_ genome equivalents per mL; p<0·0001). There was no difference in the density of PCV13 serotypes between the study periods (figure 2; appendix pp 28–31). The findings were similar when stratifying by age, although the density of non-typeable *S pneumoniae* was only significantly lower in the COVID-19 (2·99 [95% CI 2·75–3·24]) than in the pre-COVID-19 period (4·87 [4·44–5·30]; p<0·0001) in children aged 24 months or younger.

The serotype-specific densities are shown in the appendix (pp 28–31). Briefly, the densities of vaccine serotype 4 (5·94 [95% CI 5·89–5·98] *vs* 2·08 [1·78–2·38] log_10_ genome equivalents per mL; p=0·019), serotype 7AF (2·97 [2·44–3·49] *vs* 2·52 [0·98–4·06] log_10_ genome equivalents per mL; p=0·004), and non-vaccine serotypes 35A, 35C, and 42 (2·72 [1·91–3·52] *vs* 1·96 [1·46–2·46] log_10_ genome equivalents per mL; p=0·040) were higher in the COVID-19 period than in the pre-COVID-19 period. By contrast, the densities of serotype 19F (4·23 [4·06–4·40] *vs* 4·63 [4·47–4·78] log_10_ genome equivalents per mL; p=0·0067) and serotype 34 (2·87 [2·35–3·39] *vs* 3·58 [3·15–4·02] log_10_ genome equivalents per mL; p=0·024) were lower in the COVID-19 period than in the pre-COVID-19 period.

The number of different colonising serotypes or serogroups in the pre-COVID-19 period was 57 whereas in the COVID-19 period it was 43. Among children with pneumococcus colonisation, fewer were co-colonised in the COVID-19 period than in the pre-COVID-19 period by two serotypes (34 [12%] of 274 *vs* 57 [20%] of 282, aOR 0·82 [95% CI 0·7–0·96]), three serotypes (eight [3%] of 274 *vs* 20 [7%] of 282, 0·71 [0·53–0·94]), and four or more serotypes (one [<1%] of 274 *vs* 18 [6%] of 282, 0·37 [0·19–0·73]; appendix p 32). The serotype-specific ranking is detailed in the appendix (pp 33–36).

**Figure 3:**
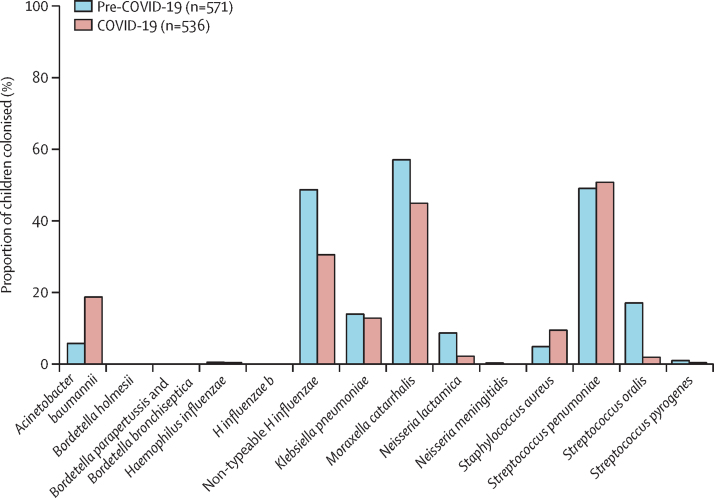
Prevalence of bacterial colonisers in children aged 0–60 months in the pre-COVID-19 period and the COVID-19 period

In the multivariable adjusted analysis, the prevalence of *A baumannii* (102 [19%] of 536 *vs* 34 [6%] of 571, aOR 1·55 [95% CI 1·35–1·77]) and *S aureus* (52 [10%] of 536 *vs* 29 [5%] of 571, 1·28 [1·10–1·50]) were higher in the COVID-19 compared with the pre-COVID-19 period. By contrast, the prevalence of non-typeable *H influenzae* (65 [31%] of 536 *vs* 280 [49%] of 571, 0·77 [0·71–0·84]), *M catarrhalis* (242 [45%] of 536 *vs* 328 [57%] of 571, 0·85 [0·79–0·92]), *N lactamica* (13 [2%] of 536 *vs* 51 [9%] of 571, 0·64 [0·52–0·78]) and *S oralis* (11 [2%] of 536 *vs* 99 [17%] of 571, 0·46 [0·37–0·57]) were lower in the COVID-19 period (figure 3, appendix pp 37–38). These findings were similar when stratifying by age, although the prevalence of *S aureus* was only significantly higher in the COVID-19 (28 [10%] of 269) than in the pre-COVID-19 period (ten [4%] of 282, 1·48 [1·15–1·89]) in children older than 24 months, and the prevalence of *M catarrhalis* was only significantly lower in the COVID-19 period (116 [43%] of 267) than in the pre-COVID 19 period (173 [60%] of 289, 0·80 [0·71–0·90]) in children aged 24 months or younger.

**Figure 4:**
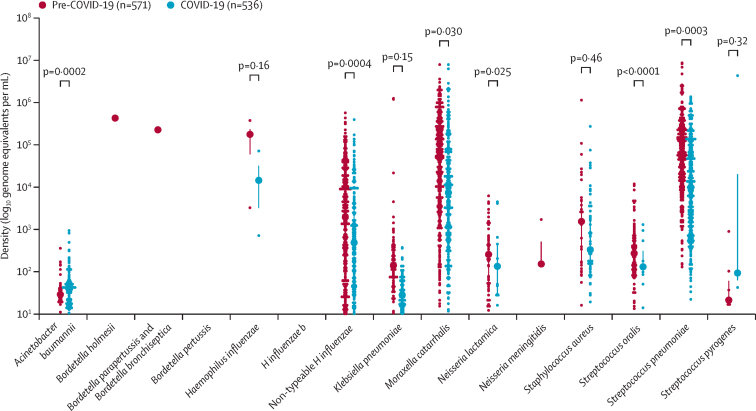
Mean log_10_ density (genome equivalents per mL) of bacterial colonisers in children aged 0–60 months in the pre-COVID-19 period and the COVID-19 period

Densities of *Bordetella* spp, *H influenzae*, *N meningitidis, A baumannii, N lactamica, S aureus, S oralis,* and *S pyogenes* did not differ between periods; however the densities of *K pneumoniae*, *M catarrhalis*, and non-typeable *H influenzae* were lower in the COVID-19 compared with the pre-COVID-19 period (figure 4; appendix p 39). These findings were similar when stratified by age groups of 24 months or younger and those older than 24 months.

No difference was observed between the pre-COVID-19 and COVID-19 periods in the number of children carrying two or three bacteria species concurrently, although concurrent colonisation by four or more bacteria was less common in the COVID-19 period (53 [12%] of 433) than in the pre-COVID-19 period (92 [10%] of 495, 0·85 [0·75–0·96]; appendix p 40). Concurrent bacterial species colonisation is detailed in the appendix (p 41).

Testing for respiratory viruses was only done on samples from the COVID-19 period. Rhinovirus was the most prevalent virus (in 71 [13%] of 536, 95% CI 10–16) in the COVID-19 period, followed by endemic coronavirus OC43 (in 12 [2%] of 536, 1–4) and SARS-CoV-2 (in eight [2%] of 536, 1–3); appendix p 42. The prevalence of the other endemic coronaviruses was less than 1% (HKU1 in one of 536 [95% CI 0–1] and Cov229E in two of 536 [0–1]). The prevalence of influenza A and B was 1% (both in three of 536 [0·1–2]), RSVA and RSVB was 1% (in seven of 536 [0·5–3] and in five of 536 [0·3–2], respectively), PIV1 was 1% (in four of 536 [0·2–2]), and PIV3 was less than 1% (in one of 536 [0–1]; appendix p 42). The density for the viral targets ranged from 2·07 to 5·27 log_10_ genome equivalents per mL (appendix p 43).

## Discussion

Our study provides in-depth analyses of the colonisation prevalence and density of *S pneumoniae*, together with multiple other bacterial colonisers during the COVID-19 pandemic compared with the pre-pandemic period. We observed significant reductions in the colonisation prevalence of pneumococcal PCV13 serotypes, non-typeable *H influenzae, M catarrhalis, S oralis*, and *N lactamica*. Conversely, we observed a higher colonisation prevalence of *A baumannii, S aureus*, and non-typeable pneumococcus during the COVID-19 period*.*

Similar to other studies^10^, ^11^, ^12^ we report no difference in the overall colonisation prevalence of *S pneumoniae*; however, we did observe a 13% relative reduction in the prevalence of overall colonisation with PCV13 serotypes. Furthermore, there was a lower density of overall and non-typeable *S pneumoniae* colonisation during the COVID-19 compared with pre-COVID-19 periods. This finding is in contrast to an Israeli study which did not report any reductions in the pneumococcal carriage density in children younger than 5 years,^13^ although in that study semi-quantitative methods were used to measure density, which could have contributed to the difference between the studies. Nevertheless, it is plausible that the lower rates of invasive pneumococcal disease reported in our setting during the COVID-19 pandemic were a result of the net reduction in overall pneumococcal density, as higher density of colonisation is a risk factor for pneumococcal disease.^14^ The societal restrictions in South Africa, including school closures, might have disrupted the acquisition of PCV13 serotypes during the COVID-19 period since school-age children remaine the biggest drivers for the transmission of PCV13 serotypes. Also, the lower proportion of children concurrently colonised with multiple pneumococcal serotypes in the COVID-19 period could be associated with non-pharmaceutical interventions.

We also report higher colonisation prevalence (12%) of non-typeable *S pneumoniae* in the COVID-19 relative to pre-COVID-19 period (6%). The potential of non-typeable *S pneumoniae* to cause invasive pneumococcal disease is unclear; however, their ability to carry antimicrobial resistance genes that could be a source of DNA for capsular pneumococci remains a concern. Further studies are warranted to better understand the pathophysiological mechanisms and the basis upon why non-pharmaceutical interventions and their consequences could have had a differential effect on the colonisation by PCV13 serotypes compared with non-typeable *S pneumoniae*.

Deployment of non-pharmaceutical interventions during the COVID-19 pandemic has been associated with reductions in the prevalence of other respiratory pathogens seasonally.^15^, ^16^ As infections by respiratory viruses could increase pneumococcal colonising bacterial load,^17^ disruptions in the circulation of respiratory viruses during the COVID-19 period could partly explain the lower density of overall *S pneumoniae* reported in the COVID-19 period. The supposed effect of interruptions in viral transmission on pneumococcal load could explain the observed reduction in invasive pneumococcal disease,^17^ which is consistent with no increases in prevalence of pneumococcal colonisation observed in our study. This possible effect is further corroborated by findings in Israel which reported a temporary decline in invasive pneumococcal disease in children during the COVID-19 pandemic that was associated with the decline in the prevalence of respiratory virus infections.^13^

During the COVID-19 period compared with the pre-COVID-19 period we observed reductions in *N lactamica* (3·7-fold), *S oralis* (8·4-fold)*, M catarrhalis* (1·3-fold), and non-typeable *H influenzae* (1·6-fold). In 2018 (ie, the pre-COVID-19 period), 64% of invasive *H influenzae* disease in South Africa was attributed to non-typeable *H influenzae*, with non-typeable *H influenzae* invasive disease being highest in infants (2·9 cases per 100 000).^18^ Furthermore, non-type b *H influenzae* has been attributed as the aetiology of 11·81% of community-acquired pneumonia resulting in hospitalisation in low-to-middle-income countries, including South Africa.^19^ The reduced carriage of non-typeable *H influenzae* in our study could partly explain the 30% reduction in incidence of all-cause pneumonia hospitalisation in children younger than 60 months observed in 2020 in our setting,^19^, ^20^ with the prevalence of non-typeable *H influenzae* colonisation possibly already having declined in 2020 when societal restrictions were most stringent in South Africa.

Conversely, the prevalence of colonisation by *A baumannii* increased 3·2-fold from the pre-COVID-19 to the COVID-19 period. The increase in colonisation prevalence of *A baumannii,* regarded by WHO as a priority organism that poses a threat to public health, is of concern.^21^ It is plausible that the high force of hospitalisation due to COVID-19 resulted in an increased acquisition of hospital-acquired *A baumannii*, which spilled over into the community and to individuals who were not hospitalised. Speculatively, reductions in the colonisation prevalence of pneumococcus, *H Influenzae*, and *M catarrhalis* might have given *S aureus* and *A baumannii* the opportunity to colonise the vacant nasopharyngeal niche during the COVID-19 period. Further investigations are warranted to understand the clinical relevance of our observation*.*

Strengths of our study included the large number of respiratory organisms and *S pneumoniae* serotypes (123 targets in total) that were tested for. The method we used allowed for quantification and we were also able to report on the density of each organism. Limitations of our study included the fact that viruses were tested only in the COVID-19 period. It is plausible that a small percentage of non-typeable *S pneumoniae* were typeable pneumococci as our reaction set only detects 94 of the known 100 serotypes; however, the serotypes not covered by the assay (eg, 7D) are uncommon in carriage. Additionally, there was some variability in the sum of the density of serotypes compared with the average of the pneumococcal reference genes (average difference –0·42 colony-forming units per mL; 95% limit of agreement –5·28 to 4·45) and the Bland Altman plot method to detect non-typeable *S pneumoniae* co-colonisation might have thus underestimated or overestimated non-typeable *S pneumoniae* (appendix p 44). Colonisation by *S aureus* might have been underestimated as anterior nare samples are better suited to detect this bacterium. Our study was not powered to detect changes in less-prevalent individual serotypes and we did not adjust for multiple testing in this descriptive study. Lastly, differences in the study time periods when sampling was undertaken could have introduced potential confounders.

In conclusion, there is a paucity of data on the bacterial colonisation in the nasopharynx of young children (aged <5 years) during the COVID-19 pandemic, when deployment of non-pharmaceutical interventions could have altered the risk of acquisition and colonisation by respiratory organisms. Our study provides insights into the complex and dynamic relationships between co-circulating respiratory organisms during the COVID-19 pandemic. The lower prevalence of PCV13 serotypes together with other respiratory organisms including non-typeable *H influenzae* and *M catarrhalis* could have in part contributed to a decrease in all-cause lower respiratory tract infections observed in South Africa during the initial stage of the COVID-19 pandemic as reported from the study setting.^20^ Our study also identified an increase in colonisation by *A baumannii* and *S aureus* during the COVID-19 period, the pathophysiological mechanism and biological relevance of which warrant further investigation.

## Data sharing

Study data will be made available following publication to researchers who provide a methodologically sound proposal and after ethics approval is granted. Requests should be directed to the corresponding author.

## Declaration of interests

GlaxoSmithKline (GSK) awarded grant funding to the Wits-Vaccines and Infectious Diseases Analytics (VIDA) Research Unit (Chris Hani Baragwanath Hospital, Soweto, South Africa) for research related to PCVs, and GSK and Pfizer have funded Wits-VIDA Research Unit to undertake non-pneumococcal research; however, neither Pfizer or GSK contributed directly to the funding of this study. SAM's institution has received grants from Pfizer, Minervax, GSK, the Bill & Melinda Gates Foundation, and the South African Medical Research Council, and he has received honoraria and support to attend a meeting from GSK and MSD unrelated to this work. CPO has received payment from Sanofi-Aventis South Africa, and support from the Bill & Melinda Gates Foundation to attend a meeting unrelated to this work. MCN has received payment from Sanofi and is on the GAVI, the Vaccine Alliance board. AI has received support from the Wellcome Trust to attend a meeting. All other authors declare no competing interests.
