# Supplementary material for: Bacterial nasopharyngeal colonisation in children in South Africa before and during the COVID-19 pandemic: an observational study
Source: Lancet Microbe. 2024 Jan;5(1):e34–42. doi: 10.1016/S2666-5247(23)00260-4 (PMC10789613; doi:10.1016/S2666-5247(23)00260-4)
Supplement: Supplementary appendix [file mmc1.pdf]

THE LANCET
Microbe

Supplementary appendix

This appendix formed part of the original submission and has been peer reviewed. We post it as supplied by the authors.

Supplement to: Olwagen CP, Downs SL, Izu A, et al. Bacterial nasopharyngeal colonisation in children in South Africa before and during the COVID-19 pandemic: an observational study. *Lancet Microbe* 2023; published online Dec 1. https://doi.org/10.1016/S2666-5247(23)00260-4.

Appendix: Bacterial nasopharyngeal colonisation in South African children prior to and during the COVID-19 pandemic: A cross-sectional study

Courtney P. Olwagen, Sarah L. Downs, Alane Izu, Lebohang Tharasimbi, Lara Van Der Merwe, Marta C. Nunes, and Shabir A. Madhi

Table of content

1. SUPPLEMENTARY TEXT
   1.1. Sampling method — Page 2
   1.2. Childhood vaccinations — Page 2
   1.3. Demographic and risk factors for colonisation — Page 2
   1.4. Modifications to qPCR protocol for qPCR testing of COVID-19 period samples. — Page 2-3
   1.5. South Africa's societal restrictions during the COVID-19 pandemic — Page 3

List of Tables

Supplementary Table 1: Summary of phased 5-level non-pharmaceutical interventions (NPIs) to prevent COVID-19 transmission applied in South Africa *. — Pages 4-5
Supplementary Table 2: Pneumococcal serotypes and other pathogens detected by the nanofluidic qPCR assay — Page 6
Supplementary Table 3: Oligonucleotide sequences for the additional assays included in the updated Fluidigm reaction-set — Page 7
Supplementary Table 4: Assay-set pool allocation for specific target amplification (STA)/Pre-Amplification (Pre-Amp) — Pages 8
Supplementary Table 5: External synthetic calibrator (gBlock™) properties — Page 9
Supplementary Table 6: External synthetic calibrator (gBlock™) sequences — Page 10-12
Supplementary Table 7: Limit of detection (LOD) for each target included in the nanofluidic qPCR panel — Page 13-15
Supplementary Table 8: Pneumococcal conjugate vaccine coverage — Page 17
Supplementary Table 9: Prevalence of PCV13 vaccine serotypes — Pages 19-20
Supplementary Table 10: Prevalence of non-vaccine serotypes — Pages 22-27
Supplementary Table 11: Pneumococcal group (overall. PCV13 serotypes. non-PCV13 serotypes) and serotype-specific carriage density from Nasopharyngeal Swab samples collected from children 0-60 months of age in the pre-Covid period (2018, N=572) and Covid-19 period (2021, N=536) — Pages 28-31
Supplementary Table 12: Concurrent pneumococcal colonization — Page 32
Supplementary Table 13: Multiple concurrent pneumococcal colonisations detected in nasopharyngeal swab samples in the pre-Covid period (2018, N=572) and Covid-19 period (2021, N=536) — Page 33-36
Supplementary Table 14: Prevalence of bacterial colonisers — Page 37-38
Supplementary Table 15: Bacterial carriage density from nasopharyngeal swab samples collected from children 0-60 months of age in the Pre-Covid period (2018, N=572) and Covid-19 period (2021, N=536) — Page 39
Supplementary Table 16: Concurrent bacterial colonisation — Page 40
Supplementary Table 17: Multiple concurrent bacterial colonisation detected in nasopharyngeal swab samples collected from children 0-60 months of age in Pre-Covid period (2018, N=572) and Covid-19 period (2021, N=536) — Page 41
Supplementary Table 18: Viral density in Nasopharyngeal Swab samples collected from children 0-60 months of age in the Covid-19 era (2021. n=536). — Page 43

List of Figures

Supplementary Figure 1: Prevalence of *Streptococcus pneumoniae* colonisation in children 0-60 months of age across the two study periods — Page 17
Supplementary Figure 2: Prevalence of PCV13 vaccine serotypes in children 0-60 months of age — Page 18
Supplementary Figure 3: Prevalence of non-vaccine serotypes in children 0-60 months of age — Page 21
Supplementary Figure 4: Viral colonization in children 0-60 months of age in the Covid-19 era (2021; n=356) — Page 42
Supplementary Figure 5: Comparison between the density of serotypes and the pneumococcal reference genes — Page 44

SUPPLEMENTARY TEXT

1.1. Sampling method

The community structure of Soweto is diverse, ranging from high-density informal settlements to suburban neighbourhoods. The national population census data collected by Statistics South Africa (STATSSA) in 2011 was used to select households for inclusion. All enumeration areas (EAs) were categorised into four sociodemographic strata (informal settlements; high-density mixed-income housing; middle-class suburbs, and mixed high-density and informal settlements) and a random selection of ten dwelling units (DU; households) was included from 45 selected EAs. Each DU was approached once during traditional hours (Monday to Friday, 09:00-17:00) and if no one was home, they were re-approached outside of traditional hours (Saturday, 09:00-17:00). If the second visit was unsuccessful or they were not eligible for enrolment then the DU was replaced with the next nearest front door.

1.2. Childhood vaccinations

Since 2011, children receive PCV13 using a 2+1 schedule (6, 14, and 40 weeks of age) as part of the South African immunisation program. Of note, the vaccine schedule in South Africa also includes the Rotavirus vaccine (RV) and DTaP-IPV//Hib (Diptheria, Tetanus, acellular Pertussis, Inactivated Polio vaccine, and *Haemophilus influenzae* type b Combined) which are given concurrently with PCV13 at 6 and 14 weeks of age, in addition to which children would have received DTaP-IPV//Hib and Hep B (Hepatitis B vaccine) at 10 weeks of age. At 9 months of age, only PCV13 will have been administered, with a measles vaccine having been given at 6 months of age.

1.3. Demographic and risk factors for colonisation

At the time of NPS collection, information on demographics and risk factors for colonisation were also collected in both surveillance periods. This included the child's daytime social contact, day-care attendance, breastfeeding status, HIV and TB infection status, exposure, and treatment (including prophylaxis), indoor air pollution exposure, history of illness including medical conditions, current symptomatic illness, recent hospitalisation (≤ 3 months), and current antibiotic usage. The PCV immunisation status (date of vaccination and batch numbers) for each child was recorded from the individual health records (Road to Health Card, RTHC), if the RTHC was not available, the child's immunisation status was ascertained from the caregiver according to the child's age. When possible, the HIV infection status of the child was recorded from the RTHC, and if unavailable, self-reporting information was recorded from the parent/guardian.

1.4. Modifications to qPCR protocol for qPCR testing of COVID-19 period samples.

The qPCR protocol described by Downs et al was modified: i) To allow for reverse transcription (rt) of viral RNA using Agpath-ID one-step-rt-PCR (Thermofisher, USA) in place of PreAmp master mix (Standard BioTools, formerly known as Fluidigm, San Francisco, CA) during the Specific target amplification (STA) step; ii) The STA multiplex pools were updated to include the additional targets (**Supp Table 3);** iii) To accommodate for the additional assays (110 total) within the 96.96 IFC, 14 assays were duplexed; iv) Lastly, three additional synthetic double-standard DNA (dsDNA) template gene fragments (gBlocks™) were designed

using the methods described previously (1) for the additional viral and pneumococcal targets and included as external calibrators in each qPCR run (**Supp Tables 4 & 5**). The 2019-nCoV N Positive Control Kit 9 plasmid (IDT – IVD, USA) was used as an internal calibrator for SARS-CoV-2. Further, assays targeting *Escherichia coli*, *Pneumocystis jiroveci*, and *Streptococcus algalactiae* were not included in the expanded reaction set, and 16SRNA was replaced with RNP, which was used as an internal control to evaluate the efficiency of the extractions. All assays in the reaction set used to evaluate both the pre-COVID-19 and COVID-19 periods had a limit of detection (LOD) of $<10^2$ CFU/ml, efficiency of 90-110%, and linear dynamic range (5-fold) and linearity: R^2 <0.98. Further, there was no difference in the qPCR results of the same samples pre-amplified with the rt-PCR compared with Standard BioTools, formerly known as Fluidigm STA reagents, or for duplexed assays compared to their respective single-plex reactions [the cycle quantification (Cq) variation was <1].

1.5. South Africa's societal restrictions during the COVID-19 pandemic.

During the Covid-19 pandemic, the government imposed societal restrictions in a 5-tiered level approach in South Africa. Level 5 was the strictest lockdown with a stay-at-home order and both international and provincial travel not permitted. During Level 4 restrictions, limited movement during certain hours (i.e., for medical care and shopping) were permitted and some businesses were allowed to operate. Schools were opened during the Level 3 restrictions and more businesses were allowed to open. Almost all businesses were re-opened in Level 2 restrictions and inter-provincial and restricted international travel were permitted. International travel and opening of all business sectors were permitted in Level 1. South Africa moved through adjusted stages of the five alert levels from the 29th of December 2020 and enrolments during the COVID-19 period were conducted during adjusted restriction levels three, two, and one. During these restrictions wearing of face masks were mandatory in all public spaces. In adjusted Level 3, schools were again closed and gatherings were restricted to 50 people. Borders were partially opened in Level 2, but non-essential travel in and out of Gauteng was still prohibited and gathering were limited to 250 people in doors and 500 outdoors. Most restriction were lifted in Level 1; however, wearing of facemasks were still mandatory.

Supplementary Table 1: Summary of phased 5-level non-pharmaceutical interventions (NPIs) to prevent COVID-19 transmission applied in South Africa *.

Date	Level	Key NPIs/Events
5-Mar-20		First confirmed COVID-19 case
15-Mar-20	SOD	State of Disaster officially declared<br>Border restrictions: Airports, Sea ports, Land Borders closed.
18-Mar-20		School closures
27-Mar-20 to 30-Apr-20	5	Border restrictions: Airports, Sea ports, Land Borders closed<br>Funerals restricted to close family; night vigils, after-funeral, and cremation gatherings, prohibited<br>No internal travel<br>Prohibition of Alcohol sales, and gatherings<br>Public recreational spaces closed<br>Stay-at-home order with only essential services operating<br>School Closures<br>Only essential shopping allowed
1-May-20 to 31-May-20	4	Border restrictions: Airports, Seaports, Land Borders closed<br>Curfew in place (21:00 to 04:00)<br>Funeral restrictions. Night vigils, after-funeral, and cremation gatherings, prohibited<br>No internal travel<br>Operation of some non-essential sectors with health protocols<br>Prohibition of Alcohol sales and gatherings<br>Public recreational spaces closed<br>Restaurants Closed<br>School Closures<br>Work from home
1-Jun-20 to 17-Aug-20	3	Alcohol permitted with limitations but Alcohol prohibition re-instated 12-Jul-12<br>Curfew in place (22:00-04:00)<br>Face masks mandatory in public spaces<br>Funerals with restrictions. Night vigils, after-funeral, and cremation gatherings, prohibited<br>Gatherings limited of 50 people indoors and 100 outdoors.<br>Health protocols in work and public spaces<br>Interprovincial travel allowed<br>Public recreational spaces closed<br>Restaurants open with restrictions, alcohol prohibited<br>Schools opened with space limitations, health protocols in place and closed on 27-Jul-20<br>Work from home
18-Aug-20 to 20-Sep-20	2	Alcohol permitted with limitations<br>Curfew (23:00-04:00)<br>Face masks mandatory in public spaces<br>Funerals with restrictions. Night vigils, after-funeral, and cremation gatherings, prohibited<br>Gatherings limited to 100 people indoors, 250 outdoors<br>Health protocols in work and public spaces<br>Interprovincial travel allowed<br>Public recreational spaces closed<br>Restaurants open
21-Sep-20 to 28-Dec-20	1	Curfew (23:00-04:00)<br>Face masks mandatory in public spaces<br>Funerals with restrictions; night vigils, after-funeral, and cremation gatherings, prohibited<br>Health protocols in work and public spaces<br>Non-essential business opened, health protocols in place<br>Public spaces opened, health protocols in place<br>Schools reopened
29-Dec-20 to 28-Feb-21	3	Adjusted level<br>Alcohol Prohibited (29-Dec-20 to 1-Feb-21)<br>Curfew (21:00-06:00)<br>Face masks mandatory in public spaces<br>Funerals with restrictions. Night vigils, after-funeral, and cremation gatherings, prohibited<br>Health protocols in work and public spaces<br>Public recreational spaces closed<br>Schools closed
1-Mar-21 to 30-May-21	1	Adjusted<br>Alcohol Prohibited over Public Holidays<br>Curfew (00:00-04:00)<br>Face masks mandatory in public spaces<br>Funerals with restrictions; night vigils, after-funeral, and cremation gatherings, prohibited<br>Health protocols in work and public spaces
31-May-21 to 15-Jun-21	2	Adjusted<br>Curfew (23:00-04:00)<br>Face masks mandatory in public spaces<br>Funerals with restrictions; night vigils, after-funeral, and cremation gatherings, prohibited<br>Gatherings restricted<br>Health protocols in work and public spaces

16-Jun-21 to 27-Jun-21	3	Adjusted<br>Curfew (22:00-04:00)<br>Face masks mandatory in public spaces<br>Funerals with restrictions; night vigils, after-funeral, and cremation gatherings, prohibited<br>Gatherings limited to 50 indoors, 100 outdoors<br>Health protocols in work and public spaces
28-Jun-21 to 25-Jul-21	4	Adjusted<br>Alcohol prohibited<br>Curfew (21:00-04:00)<br>Face masks mandatory in public spaces<br>Funerals with restrictions; night vigils, after-funeral, and cremation gatherings, prohibited<br>Gatherings prohibited; funerals allowed<br>Health protocols in work and public spaces<br>Non-essential travel in and out of Gauteng prohibited<br>Schools closed
26-Jul-21 to 12-Sep-21	3	Adjusted<br>Alcohol consumption at licenced premises prohibited after 20:00.<br>Curfew (22:00-04:00)<br>Face masks mandatory in public spaces<br>Funerals with restrictions; night vigils, after-funeral, and cremation gatherings, prohibited<br>Gatherings limited to 50 indoors, 100 outdoors<br>Health protocols in work and public spaces<br>Schools closed
13-Sep-21 to 30-Sep-21	2	Adjusted<br>Alcohol consumption at licenced premises prohibited after 22:00<br>Attendance of sporting events prohibited<br>Borders partially opened<br>Curfew (23:00-04:00)<br>Face masks mandatory in public spaces<br>Funerals with restrictions; night vigils, after-funeral, and cremation gatherings, prohibited<br>Gatherings limited to 250 indoors, 500 outdoors<br>Health protocols in work and public spaces<br>Non-essential travel in and out of Gauteng prohibited
01-Oct-21 to 04-Apr-22	1	Adjusted<br>Curfew not in place<br>Face masks mandatory in public spaces<br>Health protocols in work and public spaces<br>No restrictions in education, masking and hand hygiene required<br>Funerals allowed with restrictions; night vigils, after-funeral, and cremation gatherings, prohibited
05-Apr-22		State of Disaster ended. Interim health Regulations include mandatory masking in public spaces

* https://www.gov.za/covid-19/resources/regulations-and-guidelines-coronavirus-covid-19, accessed 22-Sep-22

Supplementary Table 2: Pneumococcal serotypes and other pathogens detected by the nanofluidic qPCR assay

Individual pneumococcal serotypes		Pneumococcal serotypes within serogroups	Bacterial targets	*Hinfluenzae subtyping*	Viral targets
Serotype 1	Serotype 21	Serogroup 7A/F	*Streptococcus pneumoniae*	Haemophilus influenzae type B	Coronavirus: CoV-OC43
Serotype 2	Serotype 22A	Serogroup 9A/V	*Haemophilus influenzae*	Non-typeable Haemophilus influenzae	Coronavirus: CoV-229E
Serotype 3	Serotype 22F	Serogroup 9L/N	*Moraxella catarrhalis*		Coronavirus: CoV-NL63
Serotype 4	Serotype 23A	Serogroup 10 C/F	*Neisseria lactamica*		Coronavirus: CoV-HKU
Serotype 5	Serotype 23B	Serogroup 11A/D	*Neisseria meningitidis*		Coronavirus: SARS-CoV-2
Serotype 6A	Serotype 23F	Serogroup 11B/C	*Staphylococcus aureus*		Respiratory syncytial virus A
Serotype 6B	Serotype 24A	Serogroup 12AF/44	*Streptococcus pyogenes*		Respiratory syncytial virus B
Serotype 6C	Serotype 27	Serogroup 15A/F	*Bordetella pertussis*		Human Metapneumovirus
Serotype 6D	Serotype 29	Serogroup 15B/C	*Bordetella holmesii*		Influenza A Virus
Serotype 8	Serotype 31	Serogroup 18B/C	*Bordetella bronchiseptica*		Influenza B Virus
Serotype 10A	Serotype 33B	Serogroup 28A/F	*Bordetella parapertussis*		Human Parainfluenza Virus 1
Serotype 10B	Serotype 33C	Serogroup 24B/F	*Klebsiella pneumoniae*		Human Parainfluenza Virus 3
Serotype 11E	Serotype 33D	Serogroup 25A/F	*Acinetobacter baumanii*		Human Rhinovirus
Serotype 11F	Serotype 34	Serogroup 32A/F	*Streptococcus oralis*		
Serotype 12B	Serotype 35B	serogroup 33A/F			
Serotype 13	Serotype 35F	Serogroup 35AC/42			
Serotype 14	Serotype 36	Serogroup 7BC/40			
Serotype 16A	Serotype 37				
Serotype 16F	Serotype 38				
Serotype 17A	Serotype 39				
Serotype 18A	Serotype 41A				
Serotype 18F	Serotype 41F				
Serotype 19A	Serotype 43				
Serotype 19B	Serotype 45				
Serotype 19C	Serotype 46				
Serotype 19F	Serotype 47F				
Serotype 19F atypical	Serotype 47F				
Serotype 20	Serotype 48				

Supplementary Table 3: Oligonucleotide sequences for the additional assays included in the updated Fluidigm reaction-set

Target	Forward Primer 5' - 3'	Reverse Primer 5' - 3'	Probe 5' - 3'
S. pneumoniae serotype 19C†	AATGGTTTTCAGATTACTTGATAGCTC	CGTTCCTTATGAGAGTGGTCAAG	TGTTCCTGCCCCCACATAATGAACT
S. pneumoniae serotype 19F atypical†	GTCCTTAGTTCTGGTTATTCGGG	GGATGAGGAACCGAATCGAAG	CCAGTTATGAAGGTGAGCTAACAGTGCG
Coronavirus: CoV-OC43‡	CGATGAGGCTATTCCGACTAGGT	CCTTCCTGAGCCTTCAATATAGTAACC	TCCGCCTGGCACGGTACTCCCT
Coronavirus: CoV-229E‡	CAGTCAAATGGGCTGATGCA	AAAGGGCTATAAAGAGAATAAGGTATTCT	CCCTGACGACCACGTTGTGGTTCA
Coronavirus: CoV-NL63‡	ACCTAATAAGCCTCTTTCTCAACCC	GACCAAAGCACTGAATAACATTTTCC	AACACGCTTCCAACGAGGTTTCTTCAACTGAG
Coronavirus: CoV-HKU‡	CCTTGCGAATGAATGTGCT	TTGCATCACCACTGCTAGTACCAC	TGTGTGGCGGTTGCTATTATGTTAAGCCTG
Coronavirus: SARS-CoV-2 (N1 gene) ‡	GACCCCAAAATCAGCGAAAT	TCTGGTTACTGCCAGTTGAATCTG	ACCCCGCATTACGTTTGGTGGACC
Coronavirus: SARS-CoV-2 (N2 gene) ‡	TTACAAACATTGGCCGCAAA	GCGCGACATTCCGAAGAA	ACAATTTGCCCCCAGCGCTTCAG
RSVA: Respiratory syncytial virus A†	AGATCAACTTCTGTCATCCAGCAA	TTCTGCACATCATAATTAGGAGTATCAAT	CACCATCCAACGGAGCACAGGAGAT
RSVB: Respiratory syncytial virus B†	GATGGCTCTTAGCAAAGTCAAGTTAA	TGTCAATATTATCTCCTGTACTACGTTGAA	ATACATTAAATAAGGATCAGCTGCTGTCATCCA
HMPV: human Metapneumovirus†	GAAGARATAGACAAAGARGCAAG	TCCCACTTCTATRGTTGATGCTAG	TCAGCACCAGACACACC
FluA: Influenza A Virus†	GACCRATCCTGTCACCTCTGAC	AGGGCATTYTGGACAAAKCGTCTA	TGCAGTCCTCGCTCACTGGGCACG
FluB: Influenza B Virus†	AAATACGGTGGATTAAAYAAAAGCAA	CCAGCAATAGCTCCGAAGAAA	CACCCATATTGGGCAATTTCCTATGGC
PIV1: human Parainfluenza Virus 1†	GTTGTCAATGTCTTAATTCGTATCAATAATT	GTAGCCTMCCTTCGGCACCTAA	TAGGCCAAAGATTGTTGTCGAGACTATTCCAA
PIV3: human Parainfluenza Virus 3†	AGTCATGTTCTCTAGCACTCCTAAATACA	ATTGAGCCATCATAATTGACAATATCAA	ACCTCCCAAAGTTGATGAAAGATCAGATTATGCA
hRV: Human Rhinovirus†	GGTGTGAAGAGCCSCRTGTGCT<br>GGTGTGAAGACTCGCATGTGCT<br>GGGTGYGAAGAGYCTANTGTGCT	GGACACCCAAAGTAGTYGGTY	CCGGCCCCTGAATGYGGCTAAYC

† Fam labelled probe with NFQ quencher; ‡VIC labelled probe with NFQ quencher

Supplementary Table 4: Assay-set pool allocation for specific target amplification (STA)/Pre-Amplification (Pre-Amp)

Pool A	Pool B	Pool C
1	3	2
4	6C/D	6A/C
5	7B/7C/40	7A/F
6A/B/C/D	9A/V	9L/N
8	10B	10A/B
9A/L/N/V	11A/B/C/D/E/F	11B/C
10C/F	12B	15A/F
11A/D	13	16F/18F/28A/28F
11F	15A/B/C/F	17F
12A/B/F/44/46	16A	19A
14	34/37/17A	21
16F	18B/C/F	23B
15B/C	19F	24B/F
18A/B/C/F	22A/F	25A/25F/38
19B/F	23F	27
20	25A/F	29
22F	33B/C	31
23A/B/F	35B	32A/32F
24A	46	33A/33F/37
28A/F	47A/F	33B
33D	PiaB: *Streptococcus pneumoniae*	34
17A	BexA: *Haemophilus influenzae*	35A/35C/42
35F/47F	IS481: *Bordetella pertussis/holmesii*	36
41F	Ptx: *Bordetella pertussis/bronchiseptica/parapertussis*	39
19F Atypical	pIS: *Bordetella parapertussis*	41A
LytA: *Streptococcus pneumoniae*	Mcat: *Morexella catarrhalis*	43
Hib: *Haemophilus influenzae*-b	Nlac: *Neiserria lactamica*	45
hIS: *Bordetella holmesii*	Sau: *Staphylooccus aureus*	48
Nme: *Neiserria meningitidis SodC*	Spy: *Streptococcus pyogenes*	18CF
RSVA: Respiratory syncytial virus A	Sor: *Streptococcus oralis*	19C
HMPV: human Metapneumovirus	RNP	IGA: *Haemophilus influenzae*
PIV1: human Parainfluenza Virus 1	RSVB: Respiratory syncytial virus B	BexB: *Haemophilus influenzae*
FluA: Influenza A Virus	PIV3: human Parainfluenza Virus 3	Kpn: *Kleibsiella pneumonia*
hRV: Hum an Rhinovirus	FluB: Influenza B Virus	Coronavirus: SARS-CoV-2 (N1 gene)
Aba: *Acinetobacter baumanii*	16S	Coronavirus: SARS-CoV-2 (N2 gene)
Coronavirus: CoV-OC43	Coronavirus: CoV-NL63	
Coronavirus: CoV-229E	Coronavirus: CoV-HKU	

Supplementary Table 5: External synthetic calibrator (gBlock™) properties.

gBlock™ name	Assay-Set Targets †	Length (bp)	Amount (ng/µl) ‡	Gene equivalents (Copy number)
A1: Pool A gBlock™. no. 1	1. 4. 5. 6A/B/C/D. 33D. LytA. 19F (Atypical). 7A/F. 8. 9A/L/N/V	1088	8.09	1.36×10^{10}
A2: Pool A gBlock™. no. 2	10C/F. 11A/D. 14. 20. Hib. 16F. 22F. 15B/C. 18A/B/C. 19B/F	1147	6.73	1.07×10^{10}
A3: Pool A gBlock™. no. 3	11F. 24A. 28A/F. 17A. 35F/47F. 41F. 12A/B/F/44/46. 23A/B/F. Eco. Sag. hIS1001. Nme	1225	8.53	1.27×10^{10}
B1: Pool B gBlock™. no. 1	3. 6A/C. 7B/C/40. 9A/V. 10B. 11A/B/C/E/F. 12B. 13. 15A/B/C/F. 16A. 34/37/17A. 18B/C/F. 19C	1474	3.82	4.73×10^{9}
B2: Pool B gBlock™. no. 2	19F. 22A/F. 23F. 25A/F. 33C. 35B. 46. 47A/F. Xis	1023	7.71	1.38×10^{10}
B3: Pool B gBlock™. no. 3	BexA. Nla. Mca. Spy. Sau. 16S. PiaB. PtxS1. plS1001. IS481. Sor	1122	5.13	8.34×10^{9}
C1: Pool C gBlock™. no. 1	15A/F. Ply. 11B/C. 16F/18F/28A/F. 17F. 19A. 21. 23B. 24B/F. 25A/F/38. 27. 29	1284	7.04	1.00×10^{10}
C2: Pool C gBlock™ no. 2	31. 32A/F. 33A/F/37. 33B. 34. 35A/C/42. 45. 48. 2. 10A. 9L/N	1252	5.28	7.70×10^{9}
C3: Pool C gBlock™ no 3	Aba. Kpn. 23A. BexB. IgA1. Pji. 36. 39. 41A. 43. 6C/D.	1160	4.11	6.47×10^{9}
D1: Viral gBlock™ no. 1	18CF. PIV1. PIV2. HMV	625	3.2	9.43×10^{9}
E1: Viral gBlock™ no. 2	IS481. RSVA. RSVB. FluA. FluB. HMPV. GAPDH	928	1	1.97×10^{9}
F1: Seasonal Corona gBlock™	CoVOC43. CoV229E. CoVNL63. CoVHKU1	548	2.45	1.18×10^{9}
G1: COVID-19 Plasmid	2019-nCoV-N1. 2019-nCoV-N2	Unknown	1	2.0×10^{5}

† Listed in order of position on gBlock™; ‡ Average value of 3 successive Q-bit measurements

Supplementary Table 6: External synthetic calibrator (gBlock™) sequences

gBlock™ name	gBlock™ sequence
A1	TACCAGACGATGCTTATCAATGACCCCTATATGTGTGATATCGTGCGGTAATTGAAGCTATGAGTTGTGCTTGCCCTTGTATAGGGTCTCCAGAGTTGCTGGGGCCACATCACCGCAGGTATCCCGTCGCCGGTTGGGATGACATTTCTACGCACTATCTTTCCTATTGGATGGTTAGTTGGTGATAGTTGATAAAGCATCAGCGACGGGGGTTGCTCTGGGTGCCATGAACTATTACGGGAGTATCTTATGTCTTTAATGGTTTGTCTCAGCAACTCTATTTGGCTGTGGGAATTCTAGTTTTAGGCTACTGGAATGCTGGCAGCGACGACCACCACACACACATGCAAGTTTGCACTAGAGTATGGGAAGGTGTTGTTCTGCCCTGAGCAACTGGTGCTACTTGTATCGAAGACATGGACATAATGTTAACCTATGAACTCGATCAGAGAACGTATAGTCTTGCGACATTTCATCCACAACTAGTTTTTTATCAAAAAGACCTTGGCGAGGTGAGGTAACGCATGTGGAACATACCAAGCATCTCCATCTAAGTTCTTACGCAATCTAGCAGATGAAGCTATTTGCCGAAAACGCTTGATACAGGGTATGCACGAATAACCAACCAAACAACTATTAATATGGTATATTTATGTCGCGTCCTTAGTTCTGGTTATTCGGGAATCCAGTTATGAAGGTGAGCTAACAGTGCGACACTTCGATTCGGTTCCTCATCCGCCCTACCGACAGTGGTACTAGAGGCGATGGCATGTGGCAAACCATAGTTGGCTATCGGCATGGTGGTGTTGTGAAATGATTAAGGAGGGCAAAAACGGTCTCCTAGCCACTCCAGCTATTCCCACTCATCAGTTTCCCATATGTTTCATTCTGATGGCAGATGGGTTGGGACGAGTAACGTTCGCTTCTTCAATTATTGATTGCCCACGCAATGGGGATGCTGGAATGGGCAAAGGGTAGTAAGTTAATCATGCTAACGGCTCATCGACGAGAAAATCTTGGGGAACCGATGGAGAGCCCGAGTTAAGGTGGAGCTCAAGTAAATGGTCTACCGACACTACTCTCAGAAG
A2	TTCCACGGCTCAAGTAAATGGTCTTGATTAGTAAGGCAGGATGCATAAACCAGGAGGTACCGAGTTATGGATGTTCTTATTGGCTTGTGGAACAATATTCACAGTCTTGCCCTGTTCACAATACAGAGTGGGGTTGGGAGGGTAGATGTTATCATACACATGGCGGCCCAGCTACATTTATGGCAGTTATTGCTAAAGGAAAAATACCAATAGTTGTTCCGAGATTAAAGAAGTATTGGTGAGCATGTGAATGATCATCAGATGGATTGATACAACCCATGGCGACTGAAATGTCACTAGGAGAAGATATGTCATTCGTTTGCCAATACTTGATGGTCTCAGAGTATTGCAGTAATTGATGGACTGTATTATACCATGTGCCATCCACAGTTCGTAAAAGATACTGGCTGAGGAGCTATCTATTCAGGATAAGGTCTACTTTGTGGGAGTTCAGTCGAATATATCAGAATGGTTGAGTACTTTTGACTCCGTCTGCTGATGATTGCGCATGGGTACATCTGTTCGCCATAACTTCATCTTAGCACCACAAAACTTCTCATTCTTCGAGCCTAATTATTAATCACCGAGATAGAAGCGTAAGGGGGTACTAGAGTAGATCGACAATGGACATTTTCCAAATGAAGATGTCCACCTTATATCCAACAGAACGGTAGATATTTTGATTGTGCCGAAAGGTTTAGGAATAGCTGCATTAGAGCAACTGGTATTTTTGATATTGGAGAACAAAATGCTAACTTCGTTGGAGGTGTATGGCATGGCATTCCTTTGGGATTCTTGTTACCGTTAATTAGTTTGCTACAGGTTTTAGTATTGAGAAAGCGGATGATTGTAGCGATCCAACTATACCTCTTATATTGCTTTATGAATACTATTACTGGAACGCCTGTTGTTATTCACGCCTTACGTAACCGTTGGCCCTTGTGGTGGATGGAGTAAGGCTATTCGAGAAGTGCAACTTGAAGATGAAGCCTGTGGTCATGCGAGATACGACAGAAAGACCTGAAGGAGTAGCTGCTGGAACGTTGAAATTGGTTGGGACTGATGAGGAGACTAAATCACTCACAAAGAAAATTTAACGCTTGATGTGATGACCCCATAGTGTTCGAGGTCATTCCGAGTTATCCAG
A3	GATGACCTTCGTGGCGTGCTAGTATGCATAGTGTTCGAGATGACCTGCTTCATGCCATGTGGTCCAGCTACTTTTATGGCTACTCCAATAGTTGTTCCGAGGCAAAAGAATGGGGAGCATGTGAATGATCATCTTGGAGTTGCTAATTATGGGAAGTCTGCGGGTATTTTACGATATGCTGTGCAGAGTGTGCACGTGTAAGAGATGATGAGGCATGCATATTAGTCAATGCAACTACAGGTATTTTTGATATCGGAGTAGAAAATAGTAGGTTGATTGGCGGTGCTCAGCGCTTCACAAACGTAGTAAACTAGTGAGTACGGGGTCTAGGCATCTGATTATGTCATTCGATTGCTTGGAACCTACGGTAACCCACGCCCATAATGAGCTTTTCAAACAGGAATTTTAGGATTTGCATATTGTTGGATATAGGTGGTCGTATATACTTGATGAATAAATCGGATTATTCGGACGACCAGTTGAACAGACCTATCTGTATGTTGATAATTTGTATGTCTCGCTCTAGGAATAATGCTCCTAGTTTTTGGGAGGAAGTGCTTTTACTTCTGTGCTAACAGTGGAGATATAGTTATGAATCATGAGAAAGCGGTTGCATTGATACGAAGAGAAGCATCGAAGATTATTCGCTTGCCTCTTCATGGATTTGTAAGCGGACGTGCGATTGTTCTGGAAAGCTTATTTCGGCTATTAAGGGTCTCCAGAGGTATTGTAGGTGGACTTTCCGATGCAAGAAATGTCGGTATAGATAAAGCGGAGATTACCTCATTTTTGTTGACAGTGTCATGAACGGTTGGTTATGTCCAAAGCGGCGATTTGGACGGCAGAGAAGGTACTGGAAAAAGAACTTCTGGCCTGGCAGGAGACTGCATCAGCTATCCTGGAACTCTAGTGGCTGGTGCATTGTATTTTCACCAGCTGTATTAGGTACATGCTGATCAAGTGACAACTCCAGGGCCCTCGATGCTGGGAGCCGGTGGCGACAGCGAGACAGAATCCCGTGCAGATAGGCTTTTAGCTTGAGCGCGAAGTGAGCCAAGGCGGCCACATGGTTACCCATGGCGATGATGCACACTTAGGTGATTTACCTGCATTCATGATGGCACAGCAACAAATCCTGTTTTCTCTATTATGATCCACACGGGTGGCACGATTATGCTAGTCATCACCGAATACGGTGTGGATACGTTAGCGTATGCGGCCTGCA
B1	TTAACTTTAAGCTTGATGTGATGACCTTTTGCCATAGTGTTCGAGGTCATTGGTCAGCAGAAAGTATGCATTGGTTATTGGATGTGGTTTATCGTGAAGACCATCATCAGACCCTGGATAAACGAGCTGCACCTTAATCTTATCCGGTGCATTGCTAGAGATGGTTCCTTCAGTTGATATTGATAAAGATTATTTATATATAGAAAAACTGGCAGTTTGCACTAGAGTATGGGAAGGTGTTGTTGAGCAACTGGTCTTGTATCGAAGACATGGACATAATGTAACAACTAGTCATCATCCAGATATAGTCATTCCCAATCAGGGGTTGGTGGGTCAGTAATCGATAATGAGGGAAAAATTCATCATGGGATTTACCTTCTTTTAAGAGCGTTGTACTTGCAGAGGTTGAAGGTATCCTATATACTGCTTTAGGTATGTAGCGGTTGTCAATGTGTTTCTTTCAGATATTGGCAGATTCGGGAATAGGACCTGCAATAAAATATGAGATTGGTAAGGAATATTCTGGTCAACGGATTCCAATGCACTCGGTAACTTATGAATGGAATTCGTTTAAGTGAAAAGACATGTTGACATTCAGTAGTATGGAGATACCGCATTTCTTATCGCACTATATTTTGAATCAGTCTGACCGTTTGATGATTAACATGTTTGATGGTAAGGAGAAATTAGCTATATATAGTCTAGCTGGTTGCTGATCAAAAGGTCTATGTTTCACTTTGATGATTTGGAAAGATTTTTATCTAATTGCTAAAAATCTTACTTTGAACCTAGGATATGCTGAACGATATGGATCGGATTTAGTAGTAACCCCATTGAGAGTAGTAAGAGATCATATTCAAGGTGGAAATGCATCCTCAATCAAGAATGCCATTATAGGGGAGAGATCTTGAATCAGGTAGATTGATTTCTGCTATGTCTCCGGCTTTTGTCTTCTCTGTATTAGGAAATATCATTAGGACTCAGTATTTGATTCCTAGAGGATGTTAGAACTATGTGGGATGCTAGCAGGAACTTTTCTAGGGGCTATACGCCATAATGGATTCATCCCGTGGGACGTTTCGGATTTGGACAGGGAGACAGATCGAGATGATTATCGTGGCTTCTGGATACTATGTACGAACAGATGGACTTGACCGACTATACTCCATTTGAGACGTTTATTCGGGCGAGTTAGTGAGTTGTTGTACGCCTTATGTACATGGCATTTGCAGGATTTCTAACTCTGATTGAATACTTGTATGCTTATGGTCTTTTCGATTAACAACTCTGGACGTTAGATTTTGCTGTCAGCGACGTA

	TTTACTATGAGTAAATGGTTTTCAGATTACTTGATAGCTCTGAGTTCATTATGTGGGGGCAGGAACAAATCTTGACCACTCTCATAAGGAACGTAATGAGACATGGACTTACTATAAGA<br>CTTGACAGTAGAAGGAAAAGTCAAATCTGTCTCTTAG
B2	ATCTTCCTACAGGAAGGACGAAGCCAATATACAACCTTGTACGTCTTAGTAATGGATATACAGGTTCGGGAAATTGCAACAAGAAGTGGCAGATGATTTATCATTGCTTATGGCGCAGA<br>GATGACGTATCTGGGGTGGATTGTATAGTCTATTAAATAACCCATTGGAATTGAAACGGCTTGGAGAATGTGCCCATAAGCGAATTACGGAACAGTTTATGTGGTCTTCAATTGCGAGG<br>AGATTTGATTGAGGCAGACATTGCTATTTGCGATCCTGTTCATTCTTTCTCCGGCATCAAACGTTAAGGTTTACGAAACAACGGAGGCTCTATGTGAGATGTTTTATCACCGCTTATTTAT<br>GATACCAACTAGAATCAGCAGGACACCCGCTGGACTTACTGCAATACTGCGAGTAAATTATCAAAAGATATTCCATTTTTGAGGTGCGCTATTCCCTGTCACAGTCAGAGACAGTTTCA<br>GCAAATCTTAGATGGAAGACAAGTTTGTGGATAGGACACGGTCCAACGTTTATAAGAGGTGTAGGCTATGCTCAGGGAGGATTATTTCTATTTCTAATCAAGGCATGGAGGTGGAGCAT<br>ACAAGACAATTTAAACAATATTAGTAAAGCGCAGGTCAAGCAAATATTTTATATCGAGTTGTGCAGTCTTTACAAAGGTGCCTGGTTTGGCAAGTCGAAGTTTTTATATCTCTATTGGTT<br>TGTAATCATTCTTTCTTCAATTCCTTTCTGAGCTTCGTCCAGTTCCTGGGATATTAGTAGCGATGCTGTGAATTATAGTAAAGAGGAATTGGTAGAGAGTTTGTGGATCCACTTGATGGA<br>ATGCCTGCTGCCCCGACGGATGATGGTTCAACTTTCTCAGTTTAATAGTTTAGTAAAGCAGGTGACTGGTAGGTAACACAGCAGTTGCTTGCGGTGCACTTGTAGGAGCAGCATTAGGA<br>GCCACTGGTCTCTTAGGAACTGTAACTTTGACAAATATGGAGCAAATGATATTTTACCTGGTTTAAC
B3	ACTGTGAAGAAATACTCTTCGTATGATGTTGATCCAGACTATGTGACTCGACAGAATTTTCTGAATTAGGTGATTATCTTTATGAACAGGGATGAAAGCTCGGCTTGCATTTCTCTTTCT<br>GTTGAATTTGATTGTCGCCAACAAACTATTGACAACTTCTGTTATTTTGCCCGAGAACCATTGTATCGTATTGGAGCGGACTAAAGCAAATATAGAACGTGATAAGAACCGCCAAGCCT<br>CATGAGGCGCTGACGGCGGTGAGTGCCGCTTTTACAACCACTGCTTTTGCAGCTGTTAGCCAGCCTGTTGTCTTGGCAGGCGATACAGTGGTCAGTGATCGCCGCACTCGCTACTATTTC<br>TTACCTCAAATCCGCAACTCATCAAGGATTTCTGTTACCAACTATTACTGGTTTCCAAGACATTGTGACCGGGCAATACGCAGCTACTAGTAGCTCAGCAAATGCATCACAAACAGATA<br>ATGGCGTAAATAGGTGGTTCTGAAGATCCAACAGTATATAGTGCTGAGAGGATGGGACACGGTCCCTCCTACGGGAGGCAGCAGTTGTGCCAGCAGCCGCGGTAATACGGCAAACAGT<br>AGATACCCTGGTAGTCCCATCAAGCATAAGCCATATAAGTTTTGCAATCATTGGTGGCTTAGTAAGTGCAAGCTGTAAGCGGAAAAGCACTTGCCTTATCAGGAACTTGTGTTAGTATG<br>TAAGGTACCTCATAACTGCCGCCAGCTCGTACTTCGACAATACGTCGACACTTATGGCGATAAATGCCGGCCGTATCGGATCTTTACCTTGCGATGCGTAACTGCCGTTTCGAACGCGTG<br>GAATGGCGAAGACCCAGGGCGCACGCTGTCCGTTCTATTTGAAGCCAACGGCCTGCAATCATCCGATGTACGTGGCCAAGGCCGAACGCTTCATAGTTACGCTCACACCTACCAGAACT<br>CAACAGTCGGCCTTGCGTGAGTGGGATGCTCAAGAGCCTGCTAAACCAGCAGATACGAAAGAAGCATCAAAGGCTGCTGTTGCTAGAAAGATCGCTTGGAACCTTTTAAGCACGCCGT<br>AAACGACAATCAATACCATATTCCTTATTTAGAAGCCAGTATTGAGGCTC
C1	GATATGATAGTTGATGGAGACAGGTGACATATGACTACACTGTATCAAGTTGTTCATATATTCGTTATTTAGTGAATTGCTATACTCCGATGTTGGCAGCAGCAGTAATGTAGATTCTTA<br>TTCTGCAGGGAGAGCTCACATTAGTTTAATGCTCAGCGATAGCTTTCTCCAAGTGGAAGACCCCAGCAATTCAAGTGTTCGCGGAGCGGTAAACGATTTGTTGGCTAAGTGGCATCAAG<br>AAATGGCCGCTATCAAATTTGGCGTATTGCTTATCACTTCCGTGGCAAGATTCTGGTGCTAAGATGGGGATCAACTATGCTCATAATCAGCATGCCAGATGTACCTTTGTCGTATTGGTT<br>TCGGACTCTTTCGTGGATGGTTGTACGTGGAATCGGATTTGGTCCGCATTGGACAAGGAGTTTCTATCTTAGCAAAGCGTAAATTATTTGTTGGCAACTAGTAAAGATTTCATGTCCTAT<br>AAGGGAGAAGTTTGTACATGGTCTGGATTTATGATTGCTTCTCATAAACAGGGACGCCTAGTTAGTATCATCACCAGATATCTTTCGACGACGTATCAGCTTCACCACCCAAAACGGTT<br>GACGCATTATACTTATGAAGAGGTTAAGGCTCTCAATGACAATTAGCAATAGATAATGGAGTTGATATGCCATTTGAAGGACCAGTTGTTGAAATGGCATTGCTTCGTAAAGTTGGTAT<br>TCCTGATAGTGGCTTTTTTATTGTAATTGGTATCGTCTCTTTTAGTGTTGAAGAAATTGCTCCAGAAACATATAGAGCTATTTATCTTTCGTGGTTTTTGTTAGTGGTTGAGGCTAGTCTTT<br>TGGTTTATATTAATTCCATTTCTGAAAGTAATTAGTAAGATTAACGGAAGATTGGTTGCTGACAATTTTGGGACTGTGGATGTTGGAGCTATTTTAAAAGTAGATGGAAGCAAGTCTTAC<br>GTAGAACCTCTCTGGATGAATTGCCACAGATTTGGAATATTTTGGTCGGTCACATGTCGCTTGTAGGACCAATAATAGGTAGAGGAAGTGATTCATTAGCGATTTAGCGACTGATATCCT<br>ATGTGGAAGGCGTTTGAAGGTGACTATCACGCGAGATCGATTTTGAGAGACTATTCGAGCAATTGGACGACTTCGAGTTGTGCCGTTTTTACAAACGCAGAGAAAAGACTAGGATTCA<br>GAAAAATTTTAGAGGTGGGTACGCCTTGGAAGATTCGCATGAGAAATGACGTCCTCATCAGAGAGGGATAGCAAGTTATTGTCGGCG
C2	TGATTCAACTGTTCTAATTACCGCTCCAAGAGCATTTACAAACATAGGTCGGTCATTTTATTAGCAGAAGTTTTAAGTCACGGACATCCTTGCGGTTTTACGTGGGGGGACCCTTAGTGA<br>CATCTGTAATGCTGAGAAGGCTCTCGGAGGATTGTGTACTTCCTGTTCTAGGCTTGGTGGAGCAGTGGTTGGGTTTGAACAAGAGAGACGCTATTTTCCTCTGGGAAGTGGTTTTGCTAC<br>ATCTGGAACTGGTTCAGCAACTATACGCTGGCATGGCAGAAAAGTCCTAAGGTATTTCAGACGGTCTTAGAACCTGTCCAATGAAGAGCAAGACTTGACAGTCCTGTTAGTGCACCTGT<br>ATTTAACGATAAATGGCGTGAACAACGAATAGGAGATGAAGGAGTTTTGAATGCTGTTCTGGAAGGACATGTCTTGATTGTCCGGTGGAGTAGGTCAAGATGGGAACTATTCAAGTAC<br>ATTGGCGCTCCGTTACTCAGTATATTGGGGAGAACAGACACCCTAGCTATGGGATGAACCTGTGTTTCAAGCTTCCCCTTTAGACTTTCAAAATACCCAGGACACCCGTTCAACGATAC<br>GTGATACTTTGATTTCATTTTTTGTTATGGGTGGGGAGATGAGATATTGATCTAGCTACTTGACTAAAATATTTGAACTGCTAGGGAGCGAGGTCACTAAAAGTCATACAGCGAAATCG<br>ACTCGTCTACCAAGATAGGGAGAGCAGCATTAGGGGCAGGTTTTGCTTCATATGGGAGCAACGCTGCTTATGTGTATTACTCTCCCCTGTAGCTAATGATAACTTTTGGCCGATTTTTGA<br>GCAGTTGCTCCTCTATTTGTACCAACGGGGCGTGCGTTATGGACTGGCTGATGGTTCTCTAGGTCAACGTATTGGAACTCTTAGAAATTGGGAAAATAAAGGCTATTATTGGGTCAGGAT<br>TTATGCACAGTTTGGTGGTTCTATATCCTCTCCTATCAACTATTACTCATTATACTACCTATCATTACAACTCCCTATGTGACACGGGTCTTTTCTTTGAATGATCTAGGGACTTATGGTTA<br>TTCTGATGTTCATGGCTATTATGAAGCGTGGAATTTTCTATACTGCAATAGGGCAATTCTTAGCCGGATTCTCTCACCTGTAGAATATGGTATCGTAGCAGTAGGATTCAATGTACAAGT<br>GGTATCATTATAGTGGGGGTGTTATACGGCGAAACTATTTATGTTAGTTTTCCGC
C3	GAGCTCATGTCTAAGTGAAGTGGTCGATGGTGGGTCCTGTTCCGCTATTCCATCAAGATTTAGCTCGTCGTATTGGACTTTGGCAATGCAGATATCGGTACCCAGAAAATTACTCCTCAG<br>CAAGAGGCACAGTTTGCTCCATTACAACCCAGGCCGAATATGACGAATTAACTACCGTCACCCGCCACAGATTCATGAGCAGATCACCCGTCTGTTCCACGCGTTAGTGCGTTAAGTAC<br>TCCCCTCCATTACCCATTTGGTATGGGAGTAGGGAGTGTGGGAGAGGTTCACAAACAGCACTTTCTTCAGAGGAGGAATTGGAATGATAATTGCAACACTCAGTTTCGTTTTATTACCA<br>AGCGCATTCTTCTTCGTCCATAACCTTCTCTAGCCTTGTTGTTACTGGAAACTGATCATAATGCGAAATTTGCTCGCCATACTGCACAATTGGTTTAACCGCAGGCAAAGCATTTAATCTT<br>GGCAATTTTGGGGTTACACAGACATCGACACACACGAGTACATCTACCATCACATCTACGATTACATACTCACATCAACGAGGCGGTGTAGCAATGGTTATTTCGTTCTTTGCTAATCAG

	GCAGACTGGCCTTGTCTATTCAGCCCTTCTGGTCTCATTGTAGCGGGCATTCTTGAGTTCCCAATTTACAATATAATCGCGAAGGAGAACATAACTATGGAGGACAAAAAAATGAACTA<br>ACTCAAATAGTAACGTAAGTCAGGCGTATTCTTCACAAGGGAAATACCGCAAATAAACAAGAAAATTACAGTATGTATCTCTTAAGGTTGAATAGAAAGTTTTGAGCAAATAGATGTA<br>TCCCAGTTAACACTACCTTTGAAGCTAGACTATTCGGTCGTGGGACATTAAACCAAAAGAGCTACCGAAGTTGAACTGCGAATATTTAAAGTTGAAAGAGGCTACATCAAATAGTTGGC<br>GAGCTCGGTAGGGGTGAGTACTATTGGAGTGCTTTGGAAGTTACGGTGTGATTCAGTAGTAATTAGTATGGGACAGTTGGGATGATTGGTCGTATTAGTAGATGGCGAATTGCGTGGAT<br>CGAACTGAAGAACTAATTGAAGAGGTACATTACGCCAGACCGCCATGGCATACAGTGGTTACTTACGAAAGTATAGGCTTTTTGGT
D1	TACCAGACGATGCTTATCAATGACCCCTATATGTGTGATATGGTGTGAAGAGCCCCGTGTGCTTGATCCGGCCCCTGAATGCGGCTAACCTCCCGGGACCGACTACTTTGGGTGTCCGTC<br>ACCGCAGGTATCCCGTCGCCGGTAGTTGTCAATGTCTTAATTCGTATCAATAATTATGATAGGCCAAAGATTGTTGTCGAGACTATTCCAATAATTATTTAGGTGCCGAAGGGAGGCTAC<br>TTAAATTAGGAAAAAAGATCTACATATAATGACAATAGAAAGTCATGTTCTCTAGCACTCCTAAATACAGATTTCAACTCCCAAAGTTGATGAAAGATCAGATTATGCATCATCAGGCA<br>TAGAAGATATTGTACTTGATATTGTCAATTATGATGGCTCAATCTCAACTTTGGTTTTTTGGGCCAAATTGGAGTGTTTTACAAAGTATTAGCTCGATTTGCTGTACCTTTATTTTTCATGA<br>TTTCAGGATTTTATTAAAAATCTGAGTTTATTGATAATTTCCAGTAAATTAACACAAGGAAATCTAAGAGTTGTATTTGAAAGATGGAGAGCCCGAGTTAAGGTGGAGCTCAAGTAAAT<br>GGTCTACCGACACTACTCTCAGAAG
E1	ACCGAGTTGCCCGTTAAAGTCAAGGCCGAACGCTTCATAAGTCACAGTCGGCCTTGCGTGAGTGGGAACTCTCCTGAGTGGTTACGCTCACACCTACCAGAACTCAGATCCGATGCGTA<br>ACCGTTAGATCAACTTCTGTCATCCAGCAACCTGGACACCATCCAACGGAGCACAGGAGATGTTAGGTACCGCATCATTGATACTCCTAATTATGATGTGCAGAAGTGTAAGGCCTCAT<br>AACTGCGATGGCTCTTAGCAAAGTCAAGTTAAGAGAGCATACATTAAATAAGGATCAGCTGCTGTCATCCATCGATCAACCGTTGGTTCAACGTAGTACAGGAGATAATATTGACACAT<br>ACCCATGTAGAGTTCGGAGATTTGGACCTGCGAGCGAAGTCATTCTGACCTGAAGGCTCTGCGCGCTCGGAGTTATCGACACTGGTGGAGACAGCCGCTCCAATTTGAGCACTTGCGGA<br>CTACTAATGCCACGGTCTAGGGCTCCTCCTGTTCGACAGTCATTTACCCGTCGCCAGCCGAGCCACTGTCAGTCCAAGCTGTCAGACACCATGGGGAAGGTTACCGCCGGTGAAATTTC<br>GAGAAGAAATAGACAAAGAAGCAAGCCTGGATCAGCACCAGACACACCCAGGTCGCTTAACGTCTAGCATCAACTATAGAAGTGGGAAATGGCGTCCAGTTGTCACAGACCAATCCT<br>GTCACCTCTGACGAGAGCTGCAGTCCTCGCTCACTGGGCACGACTGTGCGGACTCTATAGACGCTTTGTCCAGAATGCCCTGGACTCGTTAGCTACCTGAAAAATACGGTGGATTAAAT<br>AAAAGCAATTTACCCACCCATATTGGGCAATTTCCTATGGCTGCAGGCATACGTTCTTTCTTCGGAGCTATTGCTGGTTCGAATACCGATCGTGCAG
F1	GAGCTCATGTCTAAGTGAAGTGGTCGATGGTGGGTCCTGTTCCGCTATTCCATCAAGACGATGAGGCTATTCCGACTAGGTTTCCGCCTGGCACGGTACTCCCTCAGGGTTACTATATTG<br>AAGGCTCAGGAAGGTCTGCTCCTAATTCCAATGGCTACAGTCAAATGGGCTGATGCATCTGAACCACAACGTGGTCGTCAGGGTAGAATACCTTATTCTCTTTATAGCCCTTTGCTTGTT<br>GATAGGAAACCTAATAAGCCTCTTTCTCAACCCAGGGTTGATAAGCCTTCTCAGTTGAAGAAACCTCGTTGGAAGCGTGTTCCTACCAGAGAGGAAAATGTTATTCAGTGCTTTGGTCC<br>TCGTGATTTTAATCACAACATGAATCACATGGTGATAGATTTTATCGCCTTGCAATAGTTATGTGTGGCGGTTGCTATTATGTTAAGCCTGAGTGGTACTAGCAGTGGTGATGCAAATTG<br>GCATGGAAGTCACACCTTCGGGAACGTGGTTGACCTACACAGGTGCCAT
G1	2019-nCoV_N Positive Control (IDT)

Supp Table 7: Limit of detection (LOD) for each target included in the nanofluidic qPCR panel

Target	Limit of detection (LOD)
1	10
2	10
3	10
4	10
5	10
6A/B/C/D	10
6A/C	100
6C/D	10
7A/F	10
7B/7C/40	10
8	100
9A/L/N/V	10
9A/V	10
9L/N	10
10A/B	10
10B	10
10C/F	100
11A/B/C/D/E/F	10
11A/D	100
11B/C	100
11F	100
12A/B/F/44/46	100
12B	10
13	10
14	100
15A/B/C/F	10
15A/F	100
15B/C	100
16A	10
16F	100
16F/18F/28A/28F	10
17A	10
17F	10
18A/B/C/F	100
18B/C/F	10
18CF	100
19A	100
19B/F	100
19C	100
19F	10
19F Atypical	100
20	100
21	100

22A/F	100
22F	100
23A/B/F	100
23B	100
23F	10
24A	10
24B/F	100
25A/25F/38	100
25A/F	10
27	100
28A/F	100
29	100
31	10
32A/32F	10
33A/33F/37	10
33B	10
33B/C	10
33D	10
34/37/17A	10
34	10
35A/35C/42	10
35B	10
35F/47F	100
36	10
39	10
41A	10
41F	100
47A/F	10
43	10
45	10
46	10
48	10
Aba: *Acinetobacter baumannii*	10
IGA: *Haemophilus influenzae*	10
BexA: *Haemophilus influenzae*	10
BexB: *Haemophilus influenzae*	10
Hib: *Haemophilus influenzae*-b	100
Coronavirus: CoV-229E	10
Coronavirus: CoV-HKU	10
Coronavirus: CoV-NL63	10
Coronavirus: CoV-OC43	10
Coronavirus: SARS-CoV-2	100
FluA: Influenza A Virus	100
FluB: Influenza B Virus	100
hIS: *Bordetella holmesii*	100
HMPV: human Metapneumovirus	100

hRV: Hum an Rhinovirus	100
IS481: *Bordetella pertussis*/*holmesii*	100
Kpn: *Klebsiella pneumonia*	10
LytA: *Streptococcus pneumoniae*	10
Mcat: *Moraxella catarrhalis*	10
Nlac: *Neisseria lactamica*	10
Nme: *Neisseria meningitidis SodC*	100
PiaB: *Streptococcus pneumoniae*	10
pIS: *Bordetella parapertussis*	100
PIV1: human Parainfluenza Virus 1	100
PIV3: human Parainfluenza Virus 3	100
Ptx: *Bordetella pertussis*/*bronchiseptica*/*parapertussis*	100
RSVA: Respiratory syncytial virus A	100
RSVB: Respiratory syncytial virus B	100
Sau: *Staphylococcus aureus*	10
Sor: *Streptococcus oralis*	10
Spy: *Streptococcus pyogenes*	10

Supplementary table 8:Pneumococcal conjugate vaccine coverage

	Pre-COVID-19 era (2018)			Covid-19 era (2021)		
**Age**	**1 dose**	**2 doses**	**3 doses**	**1 dose**	**2 doses**	**3 doses**
≥ 6 - <14 weeks	22/22 (100%; 85%-100%)	-	-	16/18 (89%; 67%-97%)	-	-
≥14 - <40 weeks	68/69 (99%; 92%-100%)	68/69 (99%; 92%-100%)	-	60/62 (97%; 89%-99%)	54/62 (87%;77%-93%)	-
>40 weeks	465/469 (99%; 98%-100%)	461/469 (98%; 97%-99%)	437/469 (93%; 91%-95%)	438/446 (98%; 97-99%)	435/446 (98%; 96%-99%)	415/446 (93%; 90%-95%)

Values are number of children (%; 95% Confidence intervals)

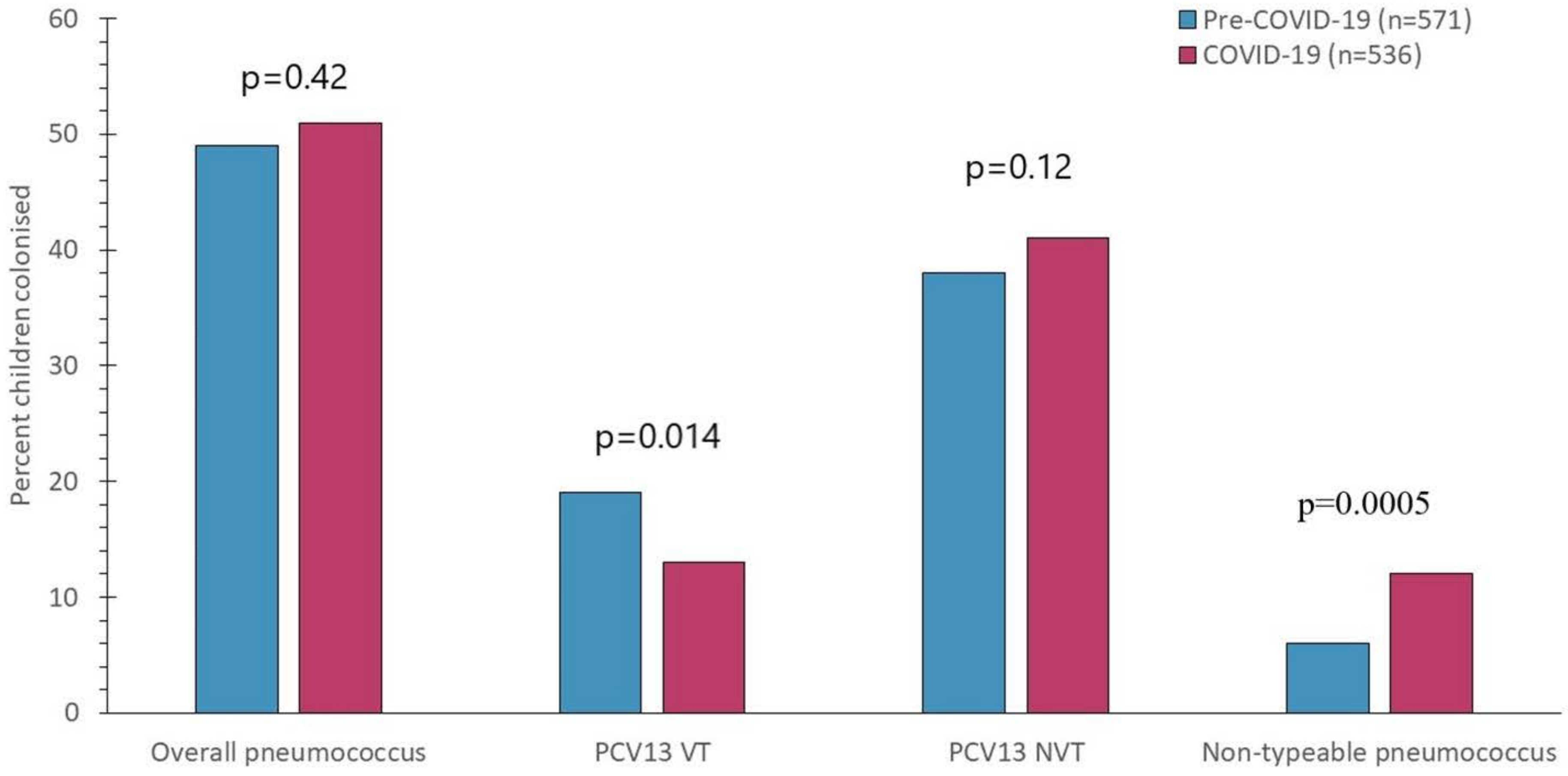

Supplementary Figure 1: Prevalence of *Streptococcus pneumoniae* colonisation in children 0-60 months of age across the two study periods

VT, vaccine serotypes including serotypes/serogroups (1, 3, 4, 5,6A, 6B, 7A/F, 9A/V, 14, 18C, 19A, 19F and 23F). PCV13-NVT, non-vaccine serotypes/serogroups not included in PCV13. non-typeable SP, non-typeable S. pneumoniae.

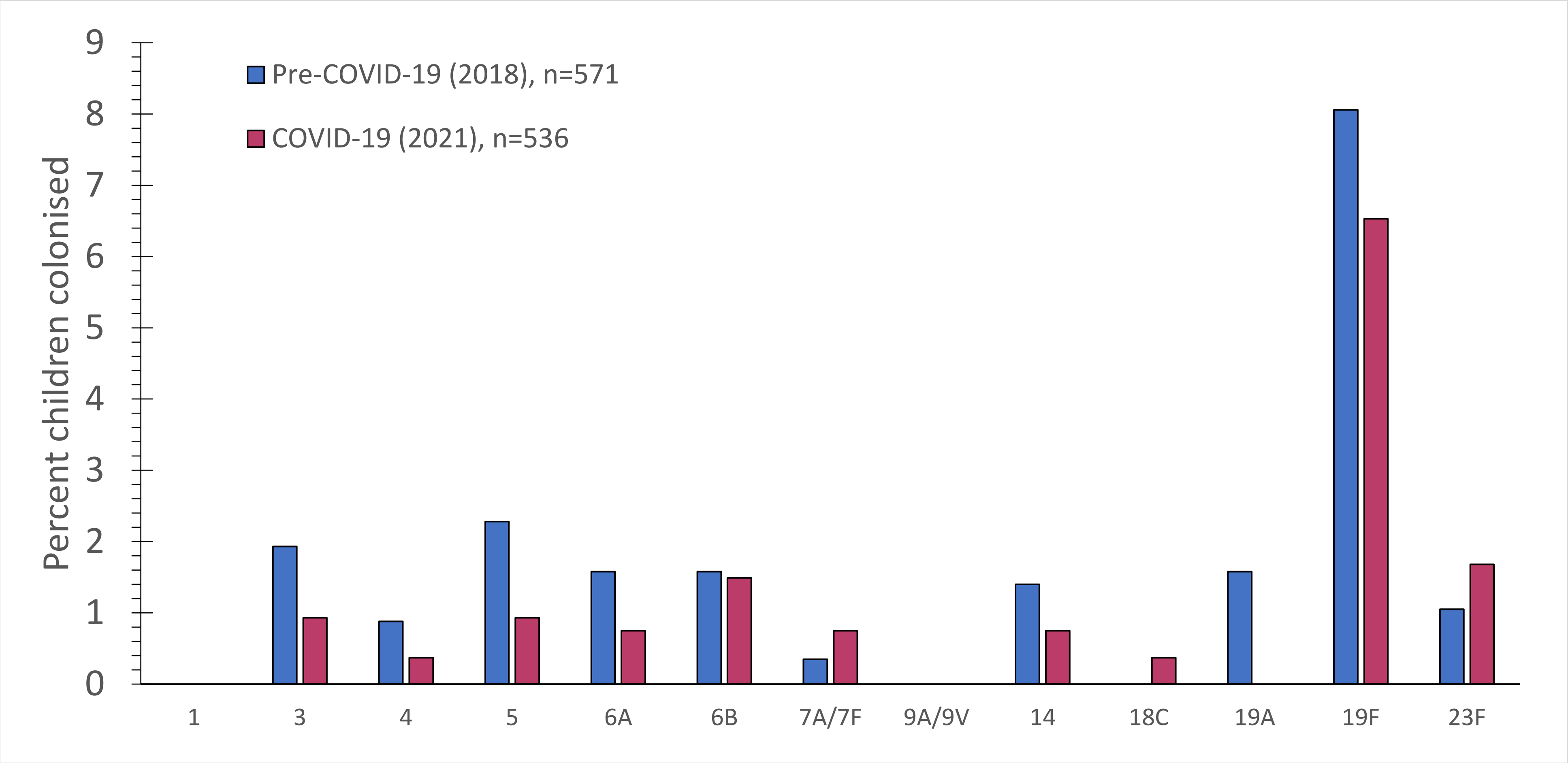

Supplementary Figure 2: Prevalence of PCV13 vaccine serotypes in children 0-60 months of age

Supplementary Table 9: Prevalence of PCV13 vaccine serotypes

	Pre-Covid-19 era (2018), N=571 n (%; 95% CI)	Covid-19 era (2021), N=536 n (%; 95% CI)	OR (95% CI); p-value	AOR (95% CI); P-value
**All age groups combined**				
**1**	0	0	-	-
**3**	11 (1.93; 1.08-3.42)	5 (0.93; 0.40-2.16)	0.48 (0.13-1.51); p=0.21	0.78 (0.55-1.12); p=0.18
**4**	5 (0.88; 0.37-2.03)	2 (0.37; 0.10-1.35)	0.42 (0.04-2.60); p=0.45	0.76 (0.44-1.32); p=0.33
**5**	13 (2.28; 1.34-3.86)	5 (0.93; 0.40-2.16)	0.4 (0.11-1.22); p=0.097	0.68 (0.47-0.99); p=0.043
**6A**	9 (1.58; 0.83-2.97)	4 (0.75; 0.29-1.90)	0.47 (0.11-1.70); p=0.27	0.8 (0.54-1.18); p=0.26
**6B**	9 (1.58; 0.83-2.97)	8 (1.49; 0.76-2.92)	0.95 (0.32-2.79); p=>0.99	0.98 (0.71-1.35); p=0.90
**7A/7F**	2 (0.35; 0.10-1.27)	4 (0.75; 0.29-1.9)	2.14 (0.30-23.72); p=0.44	1.26 (0.72-2.24); p=0.42
**9A/9V**	0	0	-	-
**14**	8 (1.4; 0.71-2.74)	4 (0.75; 0.29-1.9)	0.53 (0.12-1.99); p=0.39	0.81 (0.54-1.22); p=0.32
**18C**	0 (0; 0-0.67)	2 (0.37; 0.10-1.35)	p=0.23	-
**19A**	9 (1.58; 0.83-2.97)	0	P=0.0039	-
**19F**	46 (8.06; 6.09-10.58)	35 (6.53; 4.73-8.95)	0.8 (0.49-1.29); p=0.36	0.91 (0.78-1.07); p=0.25
**23F**	6 (1.05; 0.48-2.27)	9 (1.68; 0.89-3.16)	1.61 (0.51-5.53); p=0.44	1.15 (0.81-1.63); p= .45
**≤24 months of age**				
	**Pre-Covid-19 era (2018), N=289 n (%; 95% CI)**	**Covid-19 era (2021), N=267 n (%; 95% CI)**	**OR (95% CI); p-value (c)**	**AOR (95% CI); P-value (c)**
**1**	0	0	-	-
**3**	4 (1.38; 0.54-3.5)	1 (0.37; 0.07-2.09)	0.27 (0.01-2.73); p=0.38	0.65 (0.31-1.36); p=0.26
**4**	2 (0.69; 0.19-2.49)	0 (0; 0-1.42)	P=0.5	-
**5**	7 (2.42; 1.18-4.91)	4 (1.5; 0.58-3.79)	0.61 (0.13-2.44); p=0.55	0.74 (0.47-1.18); p=0.21
**6A**	7 (2.42; 1.18-4.91)	3 (1.12; 0.38-3.25)	0.46 (0.08-2.03); p=0.34	0.79 (0.50-1.25); p=0.32
**6B**	4 (1.38; 0.54-3.5)	4 (1.5; 0.58-3.79)	1.08 (0.20-5.88); p=>0.99	1.04 (0.65-1.66); p=0.86
**7A/F**	1 (0.35; 0.06-1.93)	1 (0.37; 0.07-2.09)	1.08 (0.01-85.23); p=>0.99	1.06 (0.42-2.68); p=0.90
**9A/V**	0	0	-	-
**14**	5 (1.73; 0.74-3.99)	1 (0.37; 0.07-2.09)	0.21 (0-1.93); p=0.22	0.62 (0.30-1.28); p=0.19
**18C**	0	1 (0.37; 0.07-2.09)	P=0.48	-
**19A**	8 (2.77; 1.41-5.37)	0	P=0.0078	-
**19F**	20 (6.92; 4.52-10.45)	21 (7.87; 5.2-11.72)	1.15 (0.58-2.29); p=0.75	1.01 (0.81-1.26); p=0.92
**23F**	5 (1.73; 0.74-3.99)	5 (1.87; 0.8-4.31)	1.08 (0.25-4.77); p=>0.99	0.99 (0.64-1.52); p=0.97
**>24 months of age**				
	**Pre-Covid-19 era (2018), N=282 n (%; 95% CI)**	**Covid-19 era (2021), N=269 n (%; 95% CI)**	**OR (95% CI); p-value (c)**	**AOR (95% CI); P-value (c)**
**1**	0	0	-	-
**3**	7 (2.48; 1.21-5.03)	4 (1.49; 0.58-3.76)	0.59 (0.13-2.37); p=0.55	0.84 (0.56-1.28); p=0.42
**4**	3 (1.06; 0.36-3.08)	2 (0.74; 0.20-2.67)	0.7 (0.06-6.13); p=>0.99	0.9 (0.49-1.65); p=0.7
**5**	6 (2.13; 0.98-4.56)	1 (0.37; 0.07-2.08)	0.17 (0-1.43); p=0.12	0.56 (0.28-1.14); p=0.11

**6A**	2 (0.71; 0.19-2.55)	1 (0.37; 0.07-2.08)	0.52 (0.01-10.10); p=>0.99	0.82 (0.37-1.83); p=0.62
**6B**	5 (1.77; 0.76-4.08)	4 (1.49; 0.58-3.76)	0.84 (0.16-3.93); p=>0.99	0.93 (0.6-1.45); p=0.76
**7A/F**	1 (0.35; 0.06-1.98)	3 (1.12; 0.38-3.23)	3.16 (0.25-166.81); p=0.36	1.44 (0.67-3.10); p=0.35
**9A/V**	0	0	-	-
**14**	5 (1.77; 0.76-4.08)	1 (0.37; 0.07-2.08)	1.05 (0.14-7.9); p=>0.99	1 (0.58-1.72); p=0.996
**18C**	0	1 (0.37; 0.07-2.08)	p=0.488	-
**19A**	1 (0.35; 0.06-1.98)	0	p=.0.99	-
**19F**	26 (9.22; 6.37-13.17)	14 (5.2; 3.13-8.55)	0.54 (0.25-1.10); p=0.073	0.82 (0.66-1.03); p=0.087
**23F**	1 (0.35; 0.06-1.98)	4 (1.49; 0.58-3.76)	4.23 (0.42-209.4); p=0.21	1.62 (0.78-3.38); p=0.20

OR. adjusted odd ratio and 95% CI calculated using logistic regression analyses. -, too few variables to calculate

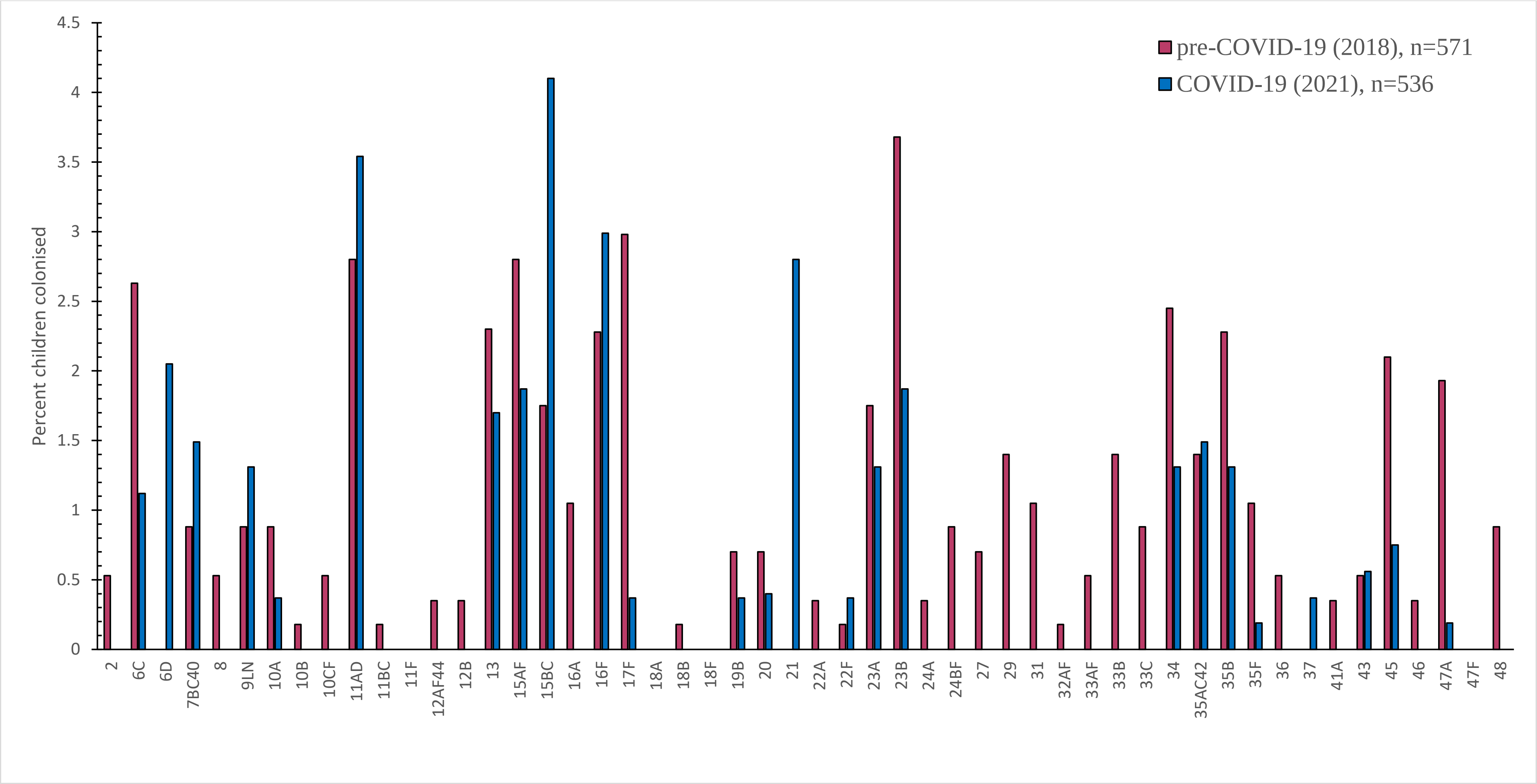

Supplementary Figure 3: Prevalence of non-vaccine serotypes in children 0-60 months of age

Supplementary Table 10: Prevalence of non-vaccine serotypes

All age groups combined					
	**Pre-Covid-19 era (2018), N=571 n (%; 95% CI)**	**Covid-19 era (2021), N=536 n (%; 95% CI)**	**p-value ***	**OR (95% CI); p-value**	**AOR (95% CI); p-value**
2	3 (0.53; 0.18-1.53)	0 (0; 0-0.71)	0.25	-	-
6C	15 (2.63; 1.60-4.29)	6 (1.12; 0.51-2.42)	0.078	0.42 (0.13-1.16); p=0.079	0.76 (0.55-1.04); p=0.087
6D	0 (0; 0-0.67)	11 (2.05; 1.15-3.64)	0.00033	-	-
7BC40	5 (0.88; 0.37-2.03)	8 (1.49; 0.76-2.92)	0.41	1.71 (0.49-6.7); p=0.41	1.22 (0.83-1.78); p=0.31
8	3 (0.53; 0.18-1.53)	3 (0.56; 0.19-1.63)	>0.99	1.07 (0.14-7.99); p=>0.99	1.02 (0.59-1.74); p=0.95
9LN	5 (0.88; 0.37-2.03)	7 (1.31; 0.63-2.67)	0.57	1.5 (0.41-6.02); p=0.57	1.15 (0.78-1.70); p=0.47
10A	5 (0.88; 0.37-2.03)	2 (0.37; 0.10-1.35)	0.45	0.42 (0.04-2.6); p=0.45	0.74 (0.43-1.28); p=0.28
10B	1 (0.18; 0.03-0.99)	0 (0; 0-0.71)	>0.99	-	-
10CF	3 (0.53; 0.18-1.53)	0 (0; 0-0.71)	0.25	-	-
11AD	16 (2.8; 1.73-4.50)	19 (3.54; 2.28-5.47)	0.50	1.27 (0.61-2.68); p=0.50	1.08 (0.86-1.35); p=0.52
11BC	1 (0.18; 0.03-0.99)	0 (0; 0-0.71)	>0.99	-	-
11F	-	-	-	-	-
12AF44	2 (0.35; 0.10-1.27)	0 (0; 0-0.71)	0.50	-	-
12B	2 (0.35; 0.10-1.27)	0 (0; 0-0.71)	0.50	-	-
13	13 (2.28; 1.34-3.86)	9 (1.68; 0.89-3.16)	0.52	0.73 (0.27-1.87); p=0.52	0.91 (0.69-1.22); p=0.54
15AF	16 (2.8; 1.73-4.50)	10 (1.87; 1.02-3.4)	0.33	0.66 (0.27-1.56); p=0.33	0.88 (0.67-1.15); p=0.34
15BC	10 (1.75; 0.95-3.19)	22 (4.1; 2.73-6.14)	0.030	2.4 (1.08-5.73); p=0.030	1.35 (1.05-1.74); p=0.020
16A	6 (1.05; 0.48-2.27)	0 (0; 0-0.71)	0.031	-	-
16F	13 (2.28; 1.34-3.86)	16 (2.99; 1.85-4.79)	0.57	1.32 (0.59-3.02); p=0.57	1.12 (0.87-1.44); p=0.36
17A	-	-	-	-	-
17F	17 (2.98; 1.87-4.72)	2 (0.37; 0.1-1.35)	0.00073	0.12 (0.01-0.52); p=0.00073	0.5 (0.31-0.81); p=0.0054
18A	-	-	-	-	-
18B	1 (0.18; 0.03-0.99)	0 (0; 0-0.71)	>0.99	-	-
18F	-	-	-	-	-
19B	4 (0.7; 0.27-1.79)	2 (0.37; 0.1-1.35)	0.69	0.53 (0.05-3.72); p=0.69	0.81 (0.46-1.44); p=0.48
20	4 (0.7; 0.27-1.79)	2 (0.37; 0.1-1.35)	0.69	0.53 (0.05-3.72); p=0.69	0.82 (0.46-1.44); p=0.49
21	13 (2.28; 1.34-3.86)	15 (2.8; 1.70-4.57)	0.70	1.24 (0.54-2.85); p=0.70	1.08 (0.84-1.39); p=0.53
22A	2 (0.35; 0.10-1.27)	0 (0; 0-0.71)	0.50	-	-
22F	1 (0.18; 0.03-0.99)	2 (0.37; 0.10-1.35)	0.61	2.13 (0.11-126.07); p=0.61	1.34 (0.6-3.0); p=0.47
23A	10 (1.75; 0.95-3.19)	7 (1.31; 0.63-2.67)	0.63	0.74 (0.24-2.18); p=0.63	0.91 (0.66-1.26); p=0.58
23B	21 (3.68; 2.42-5.56)	10 (1.87; 1.02-3.4)	0.071	0.5 (0.21-1.12); p=0.071	0.76 (0.59-1.0); p=0.047

24A	2 (0.35; 0.10-1.27)	0 (0; 0-0.71)	0.50	-	-
24BF	5 (0.88; 0.37-2.03)	0 (0; 0-0.71)	0.063	-	-
25AF	-	-	-	-	-
27	4 (0.7; 0.27-1.79)	0 (0; 0-0.71)	0.13	-	-
28AF	-	-	-	-	-
29	8 (1.4; 0.71-2.74)	0 (0; 0-0.71)	0.0078	-	-
31	6 (1.05; 0.48-2.27)	0 (0; 0-0.71)	0.031	-	-
32AF	1 (0.18; 0.03-0.99)	0 (0; 0-0.71)	>0.99	-	-
33AF	3 (0.53; 0.18-1.53)	0 (0; 0-0.71)	0.25	-	-
33B	8 (1.4; 0.71-2.74)	0 (0; 0-0.71)	0.0078	-	-
33C	5 (0.88; 0.37-2.03)	0 (0; 0-0.71)	0.063	-	-
33D	-	-	-	-	-
34	14 (2.45; 1.47-4.07)	7 (1.31; 0.63-2.67)	0.19	0.53 (0.18-1.41); p=0.19	0.84 (0.62-1.14); p=0.26
35AC42	8 (1.4; 0.71-2.74)	8 (1.49; 0.76-2.92)	>0.99	1.07 (0.35-3.29); p=>0.99	1 (0.71-1.39); p=0.98
35B	13 (2.28; 1.34-3.86)	7 (1.31; 0.63-2.67)	0.26	0.57 (0.19-1.55); p=0.26	0.84 (0.62-1.14); p=0.26
35F	6 (1.05; 0.48-2.27)	1 (0.19; 0.03-1.05)	0.13	0.18 (0-1.46); p=0.13	0.57 (0.28-1.16); p=0.12
36	3 (0.53; 0.18-1.53)	0 (0; 0-0.71)	0.25	-	-
37	0 (0; 0-0.67)	2 (0.37; 0.1-1.35)	0.23	-	-
38	-	-	-	-	-
39	-	-	-	-	-
41A	2 (0.35; 0.10-1.27)	0 (0; 0-0.71)	0.50	-	-
41F	-	-	-	-	-
43	3 (0.53; 0.18-1.53)	3 (0.56; 0.19-1.63)	>0.99	1.07 (0.14-7.99); p=>0.99	0.94 (0.53-1.66); p=0.83
45	12 (2.1; 1.21-3.64)	4 (0.75; 0.29-1.9)	0.077	0.35 (0.08-1.17); p=0.077	0.71 (0.48-1.04); p=0.076
46	2 (0.35; 0.10-1.27)	0 (0; 0-0.71)	0.5	-	-
47A	11 (1.93; 1.08-3.42)	1 (0.19; 0.03-1.05)	0.0064	0.1 (0-0.66); p=0.0064	0.46 (0.23-0.91); p=0.025
47F	-	-	-	-	-
48	5 (0.88; 0.37-2.03)	0 (0; 0-0.71)	0.062	-	-
**≤24 months of age**					
	**Pre-Covid-19 era (2018), N=289 n (%; 95% CI)**	**Covid-19 era (2021), N=267 n (%; 95% CI)**	**p-value ***	**OR (95% CI); p-value**	**AOR (95% CI); p-value**
2	1 (0.35; 0.06-1.93)	0 (0; 0-1.42)	>0.99	-	-
6C	9 (3.11; 1.65-5.81)	2 (0.75; 0.21-2.69)	0.065	0.24 (0.02-1.15); p=0.065	0.62 (0.37-1.04); p=0.07
6D	0 (0; 0-1.31)	3 (1.12; 0.38-3.25)	0.11	-	-
7BC40	3 (1.04; 0.35-3.01)	3 (1.12; 0.38-3.25)	>0.99	1.08 (0.14-8.16); p=>0.99	1.04 (0.61-1.77); p=0.90
8	3 (1.04; 0.35-3.01)	2 (0.75; 0.21-2.69)	>0.99	0.72 (0.06-6.34); p=>0.99	0.89 (0.48-1.62); p=0.69
9LN	4 (1.38; 0.54-3.50)	5 (1.87; 0.8-4.31)	0.74	1.36 (0.29-6.92); p=0.74	1.11 (0.71-1.73); p=0.65

10A	3 (1.04; 0.35-3.01)	0 (0; 0-1.42)	0.25	-	-
10B	-	-	-	-	-
10CF	2 (0.69; 0.19-2.49)	0 (0; 0-1.42)	0.5	-	-
11AD	7 (2.42; 1.18-4.91)	6 (2.25; 1.03-4.82)	>0.99	0.93 (0.25-3.27); p=>0.99	0.93 (0.64-1.37); p=0.73
11BC	1 (0.35; 0.06-1.93)	0 (0; 0-1.42)	>0.99	-	-
11F	-	-	-	-	-
12AF44	1 (0.35; 0.06-1.93)	0 (0; 0-1.42)	>0.99	-	-
12B	1 (0.35; 0.06-1.93)	0 (0; 0-1.42)	>0.99	-	-
13	7 (2.42; 1.18-4.91)	3 (1.12; 0.38-3.25)	0.34	0.46 (0.08-2.03); p=0.34	0.78 (0.5-1.24); p=0.29
15AF	10 (3.46; 1.89-6.25)	5 (1.87; 0.80-4.31)	0.30	0.53 (0.14-1.74); p=0.30	0.83 (0.58-1.19); p=0.32
15BC	4 (1.38; 0.54-3.50)	13 (4.87; 2.87-8.15)	0.024	3.64 (1.11-15.51); p=0.024	1.56 (1.07-2.27); p=0.022
16A	3 (1.04; 0.35-3.01)	0 (0; 0-1.42)	0.25	-	-
16F	5 (1.73; 0.74-3.99)	5 (1.87; 0.80-4.31)	>0.99	1.08 (0.25-4.77); p=>0.99	1.07 (0.7-1.64); p=0.76
17A	-	-	-	-	-
17F	10 (3.46; 1.89-6.25)	0 (0; 0-1.42)	0.0019	-	-
18A	-	-	-	-	-
18B	-	-	-	-	-
18F	-	-	-	-	-
19B	2 (0.69; 0.19-2.49)	0 (0; 0-1.42)	0.50	-	-
20	3 (1.04; 0.35-3.01)	1 (0.37; 0.07-2.09)	0.63	0.36 (0.01-4.5); p=0.63	0.72 (0.34-1.54); p=0.40
21	7 (2.42; 1.18-4.91)	8 (3; 1.53-5.8)	0.80	1.24 (0.39-4.09); p=0.80	1.1 (0.78-1.55); p=0.60
22A	-	-	-	-	-
22F	0 (0; 0-1.31)	2 (0.75; 0.21-2.69)	0.23	-	-
23A	8 (2.77; 1.41-5.37)	4 (1.5; 0.58-3.79)	0.39	0.53 (0.12-2.02); p=0.39	0.81 (0.54-1.21); p=0.30
23B	7 (2.42; 1.18-4.91)	4 (1.5; 0.58-3.79)	0.55	0.61 (0.13-2.44); p=0.55	0.77 (0.49-1.21); p=0.25
24A	1 (0.35; 0.06-1.93)	0 (0; 0-1.42)	>0.99	-	-
24BF	4 (1.38; 0.54-3.50)	0 (0; 0-1.42)	0.13	-	-
25AF	-	-	-	-	-
27	3 (1.04; 0.35-3.01)	0 (0; 0-1.42)	0.25	-	-
28AF	-	-	-	-	-
29	7 (2.42; 1.18-4.91)	0 (0; 0-1.42)	0.016	-	-
31	4 (1.38; 0.54-3.50)	0 (0; 0-1.42)	0.13	-	-
32AF	-	-	-	-	-
33AF	2 (0.69; 0.19-2.49)	0 (0; 0-1.42)	0.5	-	-
33B	3 (1.04; 0.35-3.01)	0 (0; 0-1.42)	0.25	-	-
33C	1 (0.35; 0.06-1.93)	0 (0; 0-1.42)	>0.99	-	-

33D	-	-	-	-	-
34	5 (1.73; 0.74-3.99)	5 (1.87; 0.80-4.31)	>0.99	1.08 (0.25-4.77); p=>0.99	1.12 (0.72-1.74); p=0.62
35AC42	4 (1.38; 0.54-3.50)	6 (2.25; 1.03-4.82)	0.53	1.64 (0.38-7.98); p=0.53	1.14 (0.74-1.76); p=0.56
35B	7 (2.42; 1.18-4.91)	4 (1.5; 0.58-3.79)	0.55	0.61 (0.13-2.44); p=0.55	0.87 (0.57-1.31); p=0.50
35F	3 (1.04; 0.35-3.01)	0 (0; 0-1.42)	0.25	-	-
36	-	-	-	-	-
37	0 (0; 0-1.31)	1 (0.37; 0.07-2.09)	0.48	-	-
38	-	-	-	-	-
39	-	-	-	-	-
41A	-	-	-	-	-
41F	-	-	-	-	-
43	1 (0.35; 0.06-1.93)	2 (0.75; 0.21-2.69)	0.61	2.17 (0.11-128.58); p=0.61	1.11 (0.47-2.64); p=0.81
45	3 (1.04; 0.35-3.01)	1 (0.37; 0.07-2.09)	0.63	0.36 (0.01-4.5); p=0.63	0.71 (0.33-1.51); p=0.37
46	1 (0.35; 0.06-1.93)	0 (0; 0-1.42)	>0.99	-	-
47A	4 (1.38; 0.54-3.50)	1 (0.37; 0.07-2.09)	0.38	0.27 (0.01-2.73); p=0.38	0.65 (0.31-1.34); p=0.24
47F	-	-	-	-	-
48	3 (1.04; 0.35-3.01)	0 (0; 0-1.42)	0.25	-	-
**>24 months of age**					
	**Pre-Covid-19 era (2018), N=282 n (%; 95% CI)**	**Covid-19 era (2021), N=269 n (%; 95% CI)**	**p-value ***	**OR (95% CI); p-value**	**AOR (95% CI); p-value**
2	2 (0.71; 0.19-2.55)	0 (0; 0-1.41)	0.50	-	-
6C	6 (2.13; 0.98-4.56)	4 (1.49; 0.58-3.76)	0.75	0.69 (0.14-2.97); p=0.75	0.89 (0.58-1.36); p=0.60
6D	0 (0; 0-1.34)	8 (2.97; 1.51-5.76)	0.0031	-	-
7BC40	2 (0.71; 0.19-2.55)	5 (1.86; 0.8-4.28)	0.28	2.65 (0.43-28.03); p=0.28	1.45 (0.81-2.59); p=0.21
8	0 (0; 0-1.34)	1 (0.37; 0.07-2.08)	0.49	-	-
9LN	1 (0.35; 0.06-1.98)	2 (0.74; 0.20-2.67)	0.62	2.1 (0.11-124.53); p=0.62	1.34 (0.60-3.01); p=0.48
10A	2 (0.71; 0.19-2.55)	2 (0.74; 0.20-2.67)	>0.99	1.05 (0.08-14.56); p=>0.99	1.02 (0.53-1.97); p=0.96
10B	1 (0.35; 0.06-1.98)	0 (0; 0-1.41)	>0.99	-	-
10CF	1 (0.35; 0.06-1.98)	0 (0; 0-1.41)	>0.99	-	-
11AD	9 (3.19; 1.69-5.95)	13 (4.83; 2.85-8.09)	0.39	1.54 (0.60-4.16); p=0.39	1.17 (0.87-1.56); p=0.29
11BC	-	-	-	-	-
11F	-	-	-	-	-
12AF44	1 (0.35; 0.06-1.98)	0 (0; 0-1.41)	>0.99	-	-
12B	1 (0.35; 0.06-1.98)	0 (0; 0-1.41)	>0.99	-	-
13	6 (2.13; 0.98-4.56)	6 (2.23; 1.03-4.78)	>0.99	1.05 (0.28-3.98); p=>0.99	1.02 (0.70-1.5); p=0.91
15AF	6 (2.13; 0.98-4.56)	5 (1.86; 0.80-4.28)	>0.99	0.87 (0.21-3.47); p=>0.99	0.96 (0.64-1.44); p=0.84
15BC	6 (2.13; 0.98-4.56)	9 (3.35; 1.77-6.24)	0.44	1.59 (0.50-5.51); p=0.44	1.17 (0.82-1.66); p=0.38

16A	3 (1.06; 0.36-3.08)	0 (0; 0-1.41)	0.25	-	-
16F	8 (2.84; 1.44-5.50)	11 (4.09; 2.30-7.17)	0.49	1.46 (0.53-4.25); p=0.49	1.15 (0.85-1.58); p=0.37
17A	-	-	-	-	-
17F	7 (2.48; 1.21-5.03)	2 (0.74; 0.20-2.67)	0.177	0.29 (0.03-1.57); p=0.18	0.66 (0.39-1.13); p=0.13
18A	-	-	-	-	-
18B	1 (0.35; 0.06-1.98)	0 (0; 0-1.41)	>0.99	-	-
18F	-	-	-	-	-
19B	2 (0.71; 0.19-2.55)	2 (0.74; 0.20-2.67)	>0.99	1.05 (0.08-14.56); p=>0.99	1.02 (0.53-1.97); p=0.95
20	1 (0.35; 0.06-1.98)	1 (0.37; 0.07-2.08)	>0.99	1.05 (0.01-82.54); p=>0.99	1.01 (0.40-2.56); p=0.98
21	6 (2.13; 0.98-4.56)	7 (2.6; 1.27-5.27)	0.78	1.23 (0.35-4.49); p=0.78	1.07 (0.74-1.55); p=0.72
22A	2 (0.71; 0.19-2.55)	0 (0; 0-1.41)	0.50	-	-
22F	1 (0.35; 0.06-1.98)	0 (0; 0-1.41)	>0.99	-	-
23A	2 (0.71; 0.19-2.55)	3 (1.12; 0.38-3.23)	0.68	1.58 (0.18-19.02); p=0.70	1.18 (0.65-2.15); p=0.59
23B	14 (4.96; 2.98-8.16)	6 (2.23; 1.03-4.78)	0.11	0.44 (0.14-1.23); p=0.11	0.77 (0.55-1.06); p=0.11
24A	1 (0.35; 0.06-1.98)	0 (0; 0-1.41)	>0.99	-	-
24BF	1 (0.35; 0.06-1.98)	0 (0; 0-1.41)	>0.99	-	-
25AF	-	-	-	-	-
27	1 (0.35; 0.06-1.98)	0 (0; 0-1.41)	>0.99	-	-
28AF	-	-	-	-	-
29	1 (0.35; 0.06-1.98)	0 (0; 0-1.41)	>0.99	-	-
31	2 (0.71; 0.19-2.55)	0 (0; 0-1.41)	0.499	-	-
32AF	1 (0.35; 0.06-1.98)	0 (0; 0-1.41)	>0.99	-	-
33AF	1 (0.35; 0.06-1.98)	0 (0; 0-1.41)	>0.99	-	-
33B	5 (1.77; 0.76-4.08)	0 (0; 0-1.41)	0.062	-	-
33C	4 (1.42; 0.55-3.59)	0 (0; 0-1.41)	0.12	-	-
33D	-	-	-	-	-
34	9 (3.19; 1.69-5.95)	2 (0.74; 0.20-2.67)	0.064	0.23 (0.02-1.11); p=0.064	0.62 (0.37-1.04); p=0.068
35AC42	4 (1.42; 0.55-3.59)	2 (0.74; 0.20-2.67)	0.69	0.52 (0.05-3.67); p=0.69	0.81 (0.46-1.43); p=0.46
35B	6 (2.13; 0.98-4.56)	3 (1.12; 0.38-3.23)	0.51	0.52 (0.08-2.46); p=0.51	0.8 (0.50-1.28); p=0.35
35F	3 (1.06; 0.36-3.08)	1 (0.37; 0.07-2.08)	0.62	0.35 (0.01-4.36); p=0.62	0.71 (0.33-1.52); p=0.38
36	3 (1.06; 0.36-3.08)	0 (0; 0-1.41)	0.25	-	-
37	0 (0; 0-1.34)	1 (0.37; 0.07-2.08)	0.49	-	-
38	-	-	-	-	-
39	-	-	-	-	-
41A	2 (0.71; 0.19-2.55)	0 (0; 0-1.41)	0.499	-	-
41F	-	-	-	-	-

43	2 (0.71; 0.19-2.55)	1 (0.37; 0.07-2.08)	>0.99	0.52 (0.01-10.1); p=>0.99	0.82 (0.37-1.84); p=0.64
45	9 (3.19; 1.69-5.95)	3 (1.12; 0.38-3.23)	0.14	0.34 (0.06-1.39); p=0.14	0.71 (0.46-1.1); p=0.12
46	1 (0.35; 0.06-1.98)	0 (0; 0-1.41)	>0.99	-	-
47A	7 (2.48; 1.21-5.03)	0 (0; 0-1.41)	0.015	-	-
47F	-	-	-	-	-
48	2 (0.71; 0.19-2.55)	0 (0; 0-1.41)	0.50	-	-

*aOR. adjusted odd ratio and 95% CI calculated using logistic regression analyses. * When odd ratios (OR) could not be calculated, Fishers exact test was used to determine significance.*

Supplementary Table 11: Pneumococcal group (overall. PCV13 serotypes. non-PCV13 serotypes) and serotype-specific carriage density from nasopharyngeal swab samples collected from children 0-60 months of age in the pre-COVID-19 period (2018, N=572) and COVID-19 period (2021, N=536)

	All ages				≤24 months of age			>24 months of age		
Serotype/Serogroup	Study period	n*	Mean Log_10 GE/ml (95%CI)	p-value	n*	Mean Log_10 GE/ml (95%CI)	p-value	n*	Mean Log_10 GE/ml (95%CI)	p-value
**Overall SP**	Pre-covid-19	282	4.72 (4.80-4.63)		147	4.84 (4.96-4.72)		135	4.58 (4.7-4.46)	
**Overall SP**	COVID-19	274	3.96 (4.07-3.85)	<0.0001	135	4.06 (4.22-3.9)	<0.0001	139	3.87 (4.01-3.72)	<0.0001
**NVT**	Pre-covid-19	216	4.08 (4.22-3.95)		111	4.3 (4.48-4.11)		105	3.85 (4.04-3.66)	
**NVT**	COVID-19	222	3.63 (3.74-3.51)	<0.0001	105	3.74 (3.91-3.56)	0.00015	117	3.53 (3.68-3.37)	0.056
**PCV13 VT**	Pre-covid-19	106	3.98 (4.18-3.78)		57	3.97 (4.24-3.71)		49	3.99 (4.32-3.67)	
**PCV13 VT**	COVID-19	72	4.08 (4.29-3.88)	0.56	38	4.16 (4.39-3.92)	0.41	34	4 (4.36-3.65)	0.94
**Non-typeable SP**	Pre-covid-19	34	4.41 (4.75-4.06)		22	4.87 (5.30-4.44)		12	3.56 (3.9-3.22)	
**Non-typeable SP**	COVID-19	63	3.11 (3.29-2.94)	<0.0001	31	2.99 (3.24-2.75)	<0.0001	32	3.23 (3.49-2.97)	0.33
**1**	Pre-covid-19	0	-		0	-		0	-	
**1**	COVID-19	0	-	-	0	-	-	0	-	-
**3**	Pre-covid-19	11	2.44 (2.96-1.92)		4	2.55 (3.88-1.22)		7	2.38 (3-1.76)	
**3**	COVID-19	5	2.16 (3.03-1.28)	0.69	1	3.67	-	4	1.78 (2.35-1.2)	0.45
**4**	Pre-covid-19	5	2.08 (2.38-1.78)		2	2; 1.93 (3.15-0.72)		3	2.18 (2.36-1.99)	
**4**	COVID-19	2	5.94 (5.98-5.89)	0.019	0	-	-	2	5.94 (5.98-5.89)	0.021
**5**	Pre-covid-19	13	2.42 (3.22-1.61)		7	2.36 (3.12-1.61)		6	2.49 (4.28-0.7)	
**5**	COVID-19	5	2.33 (2.65-2.01)	0.86	4	2.2 (2.46-1.95)	0.86	1	2.85	-
**6A**	Pre-covid-19	9	4.46 (5.28-3.64)		7	4.56 (5.62-3.5)		2	4.11 (6.12-2.1)	
**6A**	COVID-19	4	4.42 (5.81-3.03)	0.91	3	3.87 (5.01-2.72)	0.5	1	6.09	-
**6B**	Pre-covid-19	9	3.53 (4.15-2.92)		4	3.54 (4.42-2.67)		5	3.52 (4.63-2.41)	
**6B**	COVID-19	8	4.36 (4.96-3.77)	0.11	4	4.45 (5.58-3.32)	0.27	4	4.28 (5.21-3.35)	0.37
**7AF**	Pre-covid-19	2	2.52 (4.06-0.98)		1	3.04		1	1.99	
**7AF**	COVID-19	4	2.97 (3.49-2.44)	0.004	1	3.7	-	3	2.72 (2.87-2.58)	-
**9AV**	Pre-covid-19	0	-		0	-		0	-	
**9AV**	COVID-19	0	-	-	0	-	-	0	-	-
**14**	Pre-covid-19	8	3.98 (4.34-3.62)		5	4.13 (4.7-3.57)		3	3.72 (4.15-3.28)	
**14**	COVID-19	4	3.47 (4.49-2.46)	0.38	1	3.25	-	3	3.55 (5.11-1.98)	0.78
**18C**	Pre-covid-19	0	-		0	-		0	-	
**18C**	COVID-19	2	2; 3.79 (4.97-2.61)	-	1	1; 3.39	-	1	1; 4.2	-
**19A**	Pre-covid-19	9	9; 3.8 (4.33-3.26)		8	8; 3.71 (4.31-3.12)		1	1; 4.45	
**19A**	COVID-19	0	-	-	0	-	-	0	-	-
**19F**	Pre-covid-19	46	4.63 (4.78-4.47)		20	4.54 (4.8-4.29)		26	4.7 (4.9-4.5)	
**19F**	COVID-19	35	4.23 (4.40-4.06)	0.0067	21	4.26 (4.47-4.06)	0.11	14	4.18 (4.52-3.84)	0.032
**23F**	Pre-covid-19	6	4.32 (5.28-3.36)		5	4.08 (5.15-3.01)		1	5.49	
**23F**	COVID-19	9	4.69 (5.34-4.05)	0.52	5	4.89 (5.76-4.02)	0.4	4	4.45 (5.78-3.11)	-
**2**	Pre-covid-19	3	3; 2.37 (3.67-1.08)		1	1; 2.78		2	2; 2.17 (4.75-0.42)	
**2**	COVID-19	0	-	-	0	-	-	0	-	-
**6C**	Pre-covid-19	15	4.91 (5.42-4.41)		9	4.92 (5.68-4.17)		6	4.9 (5.69-4.12)	
**6C**	COVID-19	6	4.83 (5.48-4.17)	0.87	2	4.51 (7.2-1.82)	0.4	4	4.98 (5.71-4.26)	0.95
**6D**	Pre-covid-19	0	-		0	-		0	-	
**6D**	COVID-19	11	11; 4.03 (4.6-3.47)	-	3	3; 4.12 (5.51-2.72)	-	8	8; 4 (4.74-3.26)	-
**7BC40**	Pre-covid-19	5	3.57 (4.23-2.90)		3	3.79 (5.0-2.57)		2	3.23 (4.13-2.34)	

**7BC40**	COVID-19	8	3.86 (4.60-3.12)	0.35	3	4.88 (6.5-3.26)	0.32	5	3.25 (3.72-2.78)	0.68
**8**	Pre-covid-19	3	3.56 (5.23-1.89)		3	3.56 (5.23-1.89)		0	-	
**8**	COVID-19	3	3.24 (4.26-2.23)	0.95	2	3.26 (5.44-1.08)	0.95	1	1; 3.21	-
**9LN**	Pre-covid-19	5	4.9 (6.64-3.17)		4	4.76 (7.10-2.42)		1	5.49	
**9LN**	COVID-19	7	3.67 (4.31-3.03)	0.15	5	3.32 (4.0-2.63)	0.23	2	4.54 (5.76-3.33)	-
**10A**	Pre-covid-19	5	4.04 (5.1-2.98)		3	3; 4.33 (6.2-2.45)		2	3.6 (5.77-1.43)	
**10A**	COVID-19	2	2.1 (2.8-1.39)	0.37	0	-	-	2	2.1 (2.80-1.39)	0.19
**10B**	Pre-covid-19	1	1; 1.38		0	-		1	1; 1.38	
**10B**	COVID-19	0	-	-	0	-	-	0	-	-
**10CF**	Pre-covid-19	3	3; 2.67 (3.29-2.05)		2	2; 2.9 (3.58-2.22)		1	1; 2.22	
**10CF**	COVID-19	0	-	-	0	-	-	0	-	-
**11AD**	Pre-covid-19	16	4.21 (4.65-3.78)		7	4.35 (4.87-3.84)		9	4.1 (4.83-3.37)	
**11AD**	COVID-19	19	3.81 (4.22-3.39)	0.3	6	4.46 (5.42-3.51)	0.83	13	3.5 (3.92-3.08)	0.27
**11BC**	Pre-covid-19	1	1; 2.13		1	1; 2.13		0	-	
**11BC**	COVID-19	0	-	-	0	-	-	0	-	-
**11F**	Pre-covid-19	0	-		0	-		0	-	
**11F**	COVID-19	0	-	-	0	-	-	0	-	-
**12AF44**	Pre-covid-19	2	2; 3.8 (5.20-2.41)		1	1; 4.28		1	1; 3.33	
**12AF44**	COVID-19	0	-	-	0	-	-	0	-	-
**12B**	Pre-covid-19	2	2; 3.5 (4.89-2.11)		1	1; 3.98		1	1; 3.03	
**12B**	COVID-19	0	-	-	0	-	-	0	-	-
**13**	Pre-covid-19	13	3.71 (4.42-2.99)		7	3.69 (4.57-2.8)		6	3.73 (5.17-2.29)	
**13**	COVID-19	9	3.07 (3.79-2.36)	0.29	3	3.72 (5.68-1.76)	0.87	6	2.75 (3.54-1.96)	0.27
**15AF**	Pre-covid-19	16	4.05 (4.43-3.67)		10	4.09 (4.58-3.61)		6	3.98 (4.77-3.19)	
**15AF**	COVID-19	10	4.61 (4.89-4.32)	0.082	5	4.75 (5.35-4.14)	0.38	5	4.47 (4.69-4.24)	0.25
**15BC**	Pre-covid-19	10	3.91 (4.37-3.45)		4	3.41 (4.01-2.8)		6	4.25 (4.88-3.61)	
**15BC**	COVID-19	22	3.8 (4.07-3.54)	0.68	13	3.93 (4.31-3.55)	0.52	9	3.62 (4.01-3.24)	0.18
**16A**	Pre-covid-19	6	6; 1.58 (1.97-1.2)		3	3; 1.74 (2.60-0.87)		3	3; 1.43 (1.88-0.98)	
**16A**	COVID-19	0	-	-	0	-	-	0	-	-
**16F**	Pre-covid-19	13	3.7 (4.08-3.32)		5	3.46 (3.95-2.97)		8	3.85 (4.44-3.26)	
**16F**	COVID-19	16	3.36 (3.76-2.96)	0.041	5	3.5 (4.10-2.89)	0.89	11	3.3 (3.86-2.74)	0.027
**17A**	Pre-covid-19	0	-		0	-		0	-	
**17A**	COVID-19	0	-	-	0	-	-	0	-	-
**17F**	Pre-covid-19	17	3.02 (3.53-2.51)		10	10; 3.05 (3.82-2.28)		7	2.97 (3.77-2.17)	
**17F**	COVID-19	2	2.91 (4.51-1.32)	0.77	0	-	-	2	2.91 (4.51-1.32)	0.76
**18A**	Pre-covid-19	0	-		0	-		0	-	
**18A**	COVID-19	0	-	-	0	-	-	0	-	-
**18B**	Pre-covid-19	1	1; 3.3		0	-		1	1; 3.3	
**18B**	COVID-19	0	-	-	0	-	-	0	-	-
**18F**	Pre-covid-19	0	-		0	-		0	-	
**18F**	COVID-19	0	-	-	0	-	-	0	-	-
**19B**	Pre-covid-19	4	3.15 (4.59-1.72)		2	2; 3.55 (7.77--0.67)		2	2.75 (4.40-1.11)	
**19B**	COVID-19	2	3.14 (3.79-2.49)	0.89	0	-	-	2	3.14 (3.79-2.49)	0.89
**20**	Pre-covid-19	4	4.03 (4.76-3.3)		3	4.02 (5.16-2.88)		1	4.07	
**20**	COVID-19	2	3.3 (5.28-1.32)	0.35	1	3.98	-	1	2.62	-
**21**	Pre-covid-19	13	3.67 (4.16-3.18)		7	3.37 (4.15-2.60)		6	4.02 (4.7-3.34)	
**21**	COVID-19	15	3.85 (4.35-3.35)	0.88	8	3.98 (4.73-3.23)	0.5	7	3.7 (4.52-2.88)	0.5
**22A**	Pre-covid-19	2	2; 2.03 (2.04-2.02)		0	-		2	2; 2.03 (2.04-2.02)	

**22A**	COVID-19	0	-	-	0	-	-	0	-	-
**22F**	Pre-covid-19	1	5.62		0	-		1	1; 5.62	
**22F**	COVID-19	2	3.68 (4.94-2.41)	-	2	2; 3.68 (4.94-2.41)	-	0	-	-
**23A**	Pre-covid-19	10	3.98 (4.80-3.17)		8	3.93 (4.86-3.0)		2	4.19 (8.41-0.04)	
**23A**	COVID-19	7	4.25 (4.65-3.85)	0.91	4	4.56 (4.84-4.28)	0.82	3	3.83 (4.68-2.98)	0.93
**23B**	Pre-covid-19	21	4.55 (4.91-4.19)		7	5.18 (5.84-4.53)		14	4.23 (4.63-3.84)	
**23B**	COVID-19	10	4.14 (4.82-3.46)	0.24	4	4.38 (5.86-2.91)	0.33	6	3.97 (4.88-3.07)	0.34
**24A**	Pre-covid-19	2	2; 1.74 (3.75--0.27)		1	1; 2.43		1	1; 1.05	
**24A**	COVID-19	0	-	-	0	-	-	0	-	-
**24BF**	Pre-covid-19	5	5; 3.44 (4.18-2.70)		4	4; 3.32 (4.27-2.37)		1	1; 3.92	
**24BF**	COVID-19	0	-	-	0	-	-	0	-	-
**25AF**	Pre-covid-19	0	-		0	-		0	-	
**25AF**	COVID-19	0	-	-	0	-	-	0	-	-
**27**	Pre-covid-19	4	4; 3.26 (3.46-3.06)		3	3; 3.2 (3.45-2.96)		1	1; 3.43	
**27**	COVID-19	0	-	-	0	-	-	0	-	-
**28AF**	Pre-covid-19	0	-		0	-		0	-	
**28AF**	COVID-19	0	-	-	0	-	-	0	-	-
**29**	Pre-covid-19	8	8; 2.95 (3.50-2.41)		7	7; 2.86 (3.48-2.25)		1	1; 3.59	
**29**	COVID-19	0	-	-	0	-	-	0	-	-
**31**	Pre-covid-19	6	6; 2.07 (3.35-0.78)		4	4; 2.2 (4.31-0.10)		2	2; 1.8 (4.10-0.51)	
**31**	COVID-19	0	-	-	0	-	-	0	-	-
**32AF**	Pre-covid-19	1	1; 2.67		0	-		1	1; 2.67	
**32AF**	COVID-19	0	-	-	0	-	-	0	-	-
**33AF**	Pre-covid-19	3	3; 1.72 (3.07-0.36)		2	2; 1.14 (1.19-1.09)		1	1; 2.87	
**33AF**	COVID-19	0	-	-	0	-	-	0	-	-
**33B**	Pre-covid-19	8	8; 1.52 (1.71-1.33)		3	3; 1.62 (2.11-1.13)		5	5; 1.46 (1.7-1.23)	
**33B**	COVID-19	0	-	-	0	-	-	0	-	-
**33C**	Pre-covid-19	5	5; 1.68 (2.2-1.15)		1	1; 1.18		4	4; 1.8 (2.43-1.17)	
**33C**	COVID-19	0	-	-	0	-	-	0	-	-
**33D**	Pre-covid-19	0	-		0	-		0	-	
**33D**	COVID-19	0	-	-	0	-	-	0	-	-
**34**	Pre-covid-19	14	3.58 (4.02-3.15)		5	4.03 (4.69-3.37)		9	3.34 (3.92-2.75)	
**34**	COVID-19	7	2.87 (3.39-2.35)	0.024	5	3.14 (3.74-2.55)	0.046	2	2.19 (2.78-1.61)	0.24
**35AC42**	Pre-covid-19	8	1.96 (2.46-1.46)		4	1.98 (2.78-1.18)		4	1.94 (2.88-0.99)	
**35AC42**	COVID-19	8	2.72 (3.52-1.91)	0.04	6	2.86 (3.92-1.81)	0.16	2	2.28 (4.45-0.11)	0.31
**35B**	Pre-covid-19	13	4.16 (4.45-3.87)		7	4.19 (4.64-3.74)		6	4.13 (4.6-3.65)	
**35B**	COVID-19	7	4.33 (5.21-3.46)	0.64	4	4.74 (5.92-3.55)	0.86	3	3.79 (5.65-1.94)	0.4
**35F**	Pre-covid-19	6	2.68 (3.11-2.24)		3	3; 2.77 (3.91-1.62)		3	2.59 (2.75-2.43)	
**35F**	COVID-19	1	3.34	-	0	-	-	1	3.34	-
**36**	Pre-covid-19	3	3; 1.36 (1.44-1.27)		0	-		3	3; 1.36 (1.44-1.27)	
**36**	COVID-19	0	-	-	0	-	-	0	-	-
**37**	Pre-covid-19	0	-		0	-		0	-	
**37**	COVID-19	2	2; 2.09 (3.34-0.83)	-	1	1; 1.66	-	1	1; 2.52	-
**38**	Pre-covid-19	0	-		0	-		0	-	
**38**	COVID-19	0	-	-	0	-	-	0	-	-
**39**	Pre-covid-19	0	-		0	-		0	-	
**39**	COVID-19	0	-	-	0	-	-	0	-	-
**41A**	Pre-covid-19	2	2; 1.64 (2.14-1.13)		0	-		2	2; 1.64 (2.14-1.13)	

**41A**	COVID-19	0	-	-	0	-	-	0	-	-
**41F**	Pre-covid-19	0	-		0	-		0	-	
**41F**	COVID-19	0	-	-	0	-	-	0	-	-
**43**	Pre-covid-19	3	3.16 (5.14-1.18)		1	4.84		2	2.32 (2.67-1.97)	
**43**	COVID-19	3	2.09 (2.29-1.89)	0.058	2	2.15 (2.42-1.88)	-	1	1.96	-
**45**	Pre-covid-19	12	1.86 (2.11-1.61)		3	1.75 (2.25-1.26)		9	1.9 (2.23-1.56)	
**45**	COVID-19	4	1.6 (1.67-1.53)	0.55	1	1.62	-	3	1.59 (1.70-1.49)	0.71
**46**	Pre-covid-19	2	2; 3.5 (4.89-2.11)		1	1; 3.98		1	1; 3.03	
**46**	COVID-19	0	-	-	0	-	-	0	-	-
**47A**	Pre-covid-19	11	2.17 (2.99-1.34)		4	1.93 (2.56-1.3)		7	7; 2.31 (3.67-0.94)	
**47A**	COVID-19	1	1.56	-	1	1.56	-	0	-	-
**47F**	Pre-covid-19	0	-		0	-		0	-	
**47F**	COVID-19	0	-	-	0	-	-	0	-	-
**48**	Pre-covid-19	5	5; 1.93 (2.51-1.35)		3	3; 1.64 (1.99-1.29)		2	2; 2.36 (4.3-0.43)	
**48**	COVID-19	0	-	-	0	-	-	0	-	-

n is the number of samples. Density of carriage was determined through quantitative real-time nanofluidic PCR in the Fluidigm.

Supplementary Table 12: Concurrent pneumococcal colonization

	**Pre-COVID-19 (n=571)**<br>**n (%; 95% CI)**	**COVID-19 (n=536)**<br>**n (%; 95% CI)**	**aOR; 95% CI; p-value**
**Not colonised**	289/571 (50.6; 46.4-54.8)	262/536 (48.9; 44.6-53.2)	1.03 (0.95-1.12); p=0.423
**Colonised**	282/571 (49.4; 45.3-53.5)	274 (51.1; 46.9-55.3)	
1 serotype only	187/282 (66.3; 60.6-71.6)	231/274 (84.3; 79.5-88.1)	1.42 (1.24-1.63); p<0.0001
2 serotypes	57/282 (20.2; 15.9-25.3)	34/274 (12.4; 9-16.8)	0.82 (0.7-0.96); p=0.013
3 serotypes	20/282 (7.1; 4.6-10.7)	8/274 (2.9; 1.5-5.7)	0.71 (0.53-0.94); p=0.017
≥ 4 serotypes	18/282 (6.4; 4.1-9.9)	1/274 (0.4; 0.1-2)	0.37 (0.19-0.73); p=0.0039

Supplementary Table 13: Ranking of multiple concurrent pneumococcal colonisations detected in nasopharyngeal swab samples in the pre-COVID period (2018, N=572) and COVID-19 period (2021, N=536)

Study period	Serotype/serogroup	Primary coloniser (a) n/N (%; 95%CI)	Single serotype (b) n/N (%; 95%CI)	Concurrently carried (c) n/N (%; 95%CI)	First serotype (d) n/N (%; 95%CI)	Second serotype (e) n/N (%; 95%CI)	Third serotype (f) n/N (%; 95%CI)	≥ Four serotypes (g) n/N (%; 95%CI)
Pre-COVID-19	PCV13 VT	80/106 (75.5; 66.5-82.7)	59/106 (55.7; 46.2-64.8)	47/106 (44.3; 35.2-53.8)	21/106 (19.8; 13.3-28.4)	27/106 (25.5; 18.1-34.5)	4/106 (3.8; 1.5-9.3)	6/106 (5.7; 2.6-11.8)
COVID-19	PCV13 VT	59/72 (81.9; 71.5-89.1)	49/72 (68.1; 56.6-77.7)	23/72 (31.9; 22.3-43.4)	10/72 (13.9; 7.7-23.7)	14/72 (19.4; 12-30)	5/72 (6.9; 3-15.2)	-
Pre-COVID-19	NVT	202/216 (93.5; 89.4-96.1)	128/216 (59.3; 52.6-65.6)	88/216 (40.7; 34.4-47.4)	74/216 (34.3; 28.3-40.8)	68/216 (31.5; 25.7-38)	34/216 (15.7; 11.5-21.2)	15/216 (6.9; 4.3-11.1)
COVID-19	NVT	215/222 (96.8; 93.6-98.5)	182/222 (82; 76.4-86.5)	40/222 (18; 13.5-23.6)	33/222 (14.9; 10.8-20.1)	29/222 (13.1; 9.3-18.1)	4/222 (1.8; 0.7-4.5)	1/222 (0.5; 0.1-2.5)
Pre-COVID-19	Non-typeable SP	34/34 (100; 89.8-100)	32/34 (94.1; 80.9-98.4)	2/34 (5.9; 1.6-19.1)	2/34 (5.9; 1.6-19.1)	-	-	-
COVID-19	Non-typeable SP	63/63 (100; 94.3-100)	63/63 (100; 94.3-100)	-	-	-	-	-
Pre-COVID-19	3	6/11 (54.5; 28-78.7)	5/11 (45.5; 21.3-72)	6/11 (54.5; 28-78.7)	1/11 (9.1; 1.6-37.7)	4/11 (36.4; 15.2-64.6)	-	1/11 (9.1; 1.6-37.7)
COVID-19	3	3/5 (60; 23.1-88.2)	3/5 (60; 23.1-88.2)	2/5 (40; 11.8-76.9)	-	1/5 (20; 3.6-62.4)	1/5 (20; 3.6-62.4)	-
Pre-COVID-19	4	3/5 (60; 23.1-88.2)	2/5 (40; 11.8-76.9)	3/5 (60; 23.1-88.2)	1/5 (20; 3.6-62.4)	2/5 (40; 11.8-76.9)	-	-
COVID-19	4	2/2 (100; 34.2-100)	1/2 (50; 9.5-90.5)	1/2 (50; 9.5-90.5)	1/2 (50; 9.5-90.5)	-	-	-
Pre-COVID-19	5	3/13 (23.1; 8.2-50.3)	1/13 (7.7; 1.4-33.3)	12/13 (92.3; 66.7-98.6)	2/13 (15.4; 4.3-42.2)	7/13 (53.8; 29.1-76.8)	2/13 (15.4; 4.3-42.2)	1/13 (7.7; 1.4-33.3)
COVID-19	5	1/5 (20; 3.6-62.4)	-	5/5 (100; 56.6-100)	1/5 (20; 3.6-62.4)	2/5 (40; 11.8-76.9)	2/5 (40; 11.8-76.9)	-
Pre-COVID-19	6A	7/9 (77.8; 45.3-93.7)	6/9 (66.7; 35.4-87.9)	3/9 (33.3; 12.1-64.6)	1/9 (11.1; 2-43.5)	-	1/9 (11.1; 2-43.5)	1/9 (11.1; 2-43.5)
COVID-19	6A	4/4 (100; 51-100)	4/4 (100; 51-100)	-	-	-	-	-
Pre-COVID-19	6B	5/9 (55.6; 26.7-81.1)	4/9 (44.4; 18.9-73.3)	5/9 (55.6; 26.7-81.1)	1/9 (11.1; 2-43.5)	4/9 (44.4; 18.9-73.3)	-	-
COVID-19	6B	8/8 (100; 67.6-100)	6/8 (75; 40.9-92.9)	2/8 (25; 7.1-59.1)	2/8 (25; 7.1-59.1)	-	-	-
Pre-COVID-19	7AF	-	-	2/2 (100; 34.2-100)	-	2/2 (100; 34.2-100)	-	-
COVID-19	7AF	1/4 (25; 4.6-69.9)	1/4 (25; 4.6-69.9)	3/4 (75; 30.1-95.4)	-	1/4 (25; 4.6-69.9)	2/4 (50; 15-85)	-
Pre-COVID-19	14	5/8 (62.5; 30.6-86.3)	3/8 (37.5; 13.7-69.4)	5/8 (62.5; 30.6-86.3)	2/8 (25; 7.1-59.1)	2/8 (25; 7.1-59.1)	1/8 (12.5; 2.2-47.1)	-
COVID-19	14	3/4 (75; 30.1-95.4)	2/4 (50; 15-85)	2/4 (50; 15-85)	1/4 (25; 4.6-69.9)	1/4 (25; 4.6-69.9)	-	-
Pre-COVID-19	18C							
COVID-19	18C	1/2 (50; 9.5-90.5)	1/2 (50; 9.5-90.5)	1/2 (50; 9.5-90.5)	-	1/2 (50; 9.5-90.5)	-	-
Pre-COVID-19	19A	4/9 (44.4; 18.9-73.3)	4/9 (44.4; 18.9-73.3)	5/9 (55.6; 26.7-81.1)	-	2/9 (22.2; 6.3-54.7)	-	3/9 (33.3; 12.1-64.6)
COVID-19	19A							
Pre-COVID-19	19F	43/46 (93.5; 82.5-97.8)	31/46 (67.4; 53-79.1)	15/46 (32.6; 20.9-47)	12/46 (26.1; 15.6-40.3)	2/46 (4.3; 1.2-14.5)	-	1/46 (2.2; 0.4-11.3)
COVID-19	19F	32/35 (91.4; 77.6-97)	28/35 (80; 64.1-90)	7/35 (20; 10-35.9)	4/35 (11.4; 4.5-26)	3/35 (8.6; 3-22.4)	-	-
Pre-COVID-19	23F	4/6 (66.7; 30-90.3)	3/6 (50; 18.8-81.2)	3/6 (50; 18.8-81.2)	1/6 (16.7; 3-56.4)	2/6 (33.3; 9.7-70)	-	-
COVID-19	23F	4/9 (44.4; 18.9-73.3)	3/9 (33.3; 12.1-64.6)	6/9 (66.7; 35.4-87.9)	1/9 (11.1; 2-43.5)	5/9 (55.6; 26.7-81.1)	-	-
Pre-COVID-19	2	-	-	3/3 (100; 43.9-100)	-	1/3 (33.3; 6.1-79.2)	1/3 (33.3; 6.1-79.2)	1/3 (33.3; 6.1-79.2)
COVID-19	2							
Pre-COVID-19	6C	13/15 (86.7; 62.1-96.3)	8/15 (53.3; 30.1-75.2)	7/15 (46.7; 24.8-69.9)	5/15 (33.3; 15.2-58.3)	2/15 (13.3; 3.7-37.9)	-	-
COVID-19	6C	6/6 (100; 61-100)	3/6 (50; 18.8-81.2)	3/6 (50; 18.8-81.2)	3/6 (50; 18.8-81.2)	-	-	-
Pre-COVID-19	6D							
COVID-19	6D	9/11 (81.8; 52.3-94.9)	7/11 (63.6; 35.4-84.8)	4/11 (36.4; 15.2-64.6)	2/11 (18.2; 5.1-47.7)	2/11 (18.2; 5.1-47.7)	-	-

Pre-COVID-19	7BC40	4/5 (80; 37.6-96.4)	4/5 (80; 37.6-96.4)	1/5 (20; 3.6-62.4)	-	1/5 (20; 3.6-62.4)	-	-
COVID-19	7BC40	4/8 (50; 21.5-78.5)	3/8 (37.5; 13.7-69.4)	5/8 (62.5; 30.6-86.3)	1/8 (12.5; 2.2-47.1)	3/8 (37.5; 13.7-69.4)	1/8 (12.5; 2.2-47.1)	-
Pre-COVID-19	8	2/3 (66.7; 20.8-93.9)	-	3/3 (100; 43.9-100)	2/3 (66.7; 20.8-93.9)	1/3 (33.3; 6.1-79.2)	-	-
COVID-19	8	2/3 (66.7; 20.8-93.9)	1/3 (33.3; 6.1-79.2)	2/3 (66.7; 20.8-93.9)	1/3 (33.3; 6.1-79.2)	1/3 (33.3; 6.1-79.2)	-	-
Pre-COVID-19	9LN	4/5 (80; 37.6-96.4)	-	5/5 (100; 56.6-100)	4/5 (80; 37.6-96.4)	1/5 (20; 3.6-62.4)	-	-
COVID-19	9LN	6/7 (85.7; 48.7-97.4)	5/7 (71.4; 35.9-91.8)	2/7 (28.6; 8.2-64.1)	1/7 (14.3; 2.6-51.3)	1/7 (14.3; 2.6-51.3)	-	-
Pre-COVID-19	10A	3/5 (60; 23.1-88.2)	2/5 (40; 11.8-76.9)	3/5 (60; 23.1-88.2)	1/5 (20; 3.6-62.4)	1/5 (20; 3.6-62.4)	-	1/5 (20; 3.6-62.4)
COVID-19	10A	2/2 (100; 34.2-100)	2/2 (100; 34.2-100)	-	-	-	-	-
Pre-COVID-19	10B	-	-	1/1 (100; 20.7-100)	-	-	-	1/1 (100; 20.7-100)
COVID-19	10B							
Pre-COVID-19	10CF	2/3 (66.7; 20.8-93.9)	1/3 (33.3; 6.1-79.2)	2/3 (66.7; 20.8-93.9)	1/3 (33.3; 6.1-79.2)	-	1/3 (33.3; 6.1-79.2)	-
COVID-19	10CF							
Pre-COVID-19	11AD	11/16 (68.8; 44.4-85.8)	7/16 (43.8; 23.1-66.8)	9/16 (56.2; 33.2-76.9)	4/16 (25; 10.2-49.5)	1/16 (6.2; 1.1-28.3)	4/16 (25; 10.2-49.5)	-
COVID-19	11AD	14/19 (73.7; 51.2-88.2)	10/19 (52.6; 31.7-72.7)	9/19 (47.4; 27.3-68.3)	4/19 (21.1; 8.5-43.3)	5/19 (26.3; 11.8-48.8)	-	-
Pre-COVID-19	11BC	-	-	1/1 (100; 20.7-100)	-	-	1/1 (100; 20.7-100)	-
COVID-19	11BC							
Pre-COVID-19	12AF44	1/2 (50; 9.5-90.5)	-	2/2 (100; 34.2-100)	1/2 (50; 9.5-90.5)	1/2 (50; 9.5-90.5)	-	-
COVID-19	12AF44							
Pre-COVID-19	12B	-	-	2/2 (100; 34.2-100)	-	1/2 (50; 9.5-90.5)	1/2 (50; 9.5-90.5)	-
COVID-19	12B							
Pre-COVID-19	13	11/13 (84.6; 57.8-95.7)	7/13 (53.8; 29.1-76.8)	6/13 (46.2; 23.2-70.9)	4/13 (30.8; 12.7-57.6)	2/13 (15.4; 4.3-42.2)	-	-
COVID-19	13	7/9 (77.8; 45.3-93.7)	4/9 (44.4; 18.9-73.3)	5/9 (55.6; 26.7-81.1)	3/9 (33.3; 12.1-64.6)	2/9 (22.2; 6.3-54.7)	-	-
Pre-COVID-19	15AF	14/16 (87.5; 64-96.5)	8/16 (50; 28-72)	8/16 (50; 28-72)	6/16 (37.5; 18.5-61.4)	2/16 (12.5; 3.5-36)	-	-
COVID-19	15AF	9/10 (90; 59.6-98.2)	8/10 (80; 49-94.3)	2/10 (20; 5.7-51)	1/10 (10; 1.8-40.4)	1/10 (10; 1.8-40.4)	-	-
Pre-COVID-19	15BC	8/10 (80; 49-94.3)	5/10 (50; 23.7-76.3)	5/10 (50; 23.7-76.3)	3/10 (30; 10.8-60.3)	2/10 (20; 5.7-51)	-	-
COVID-19	15BC	21/22 (95.5; 78.2-99.2)	18/22 (81.8; 61.5-92.7)	4/22 (18.2; 7.3-38.5)	3/22 (13.6; 4.7-33.3)	1/22 (4.5; 0.8-21.8)	-	-
Pre-COVID-19	16A	1/6 (16.7; 3-56.4)	1/6 (16.7; 3-56.4)	5/6 (83.3; 43.6-97)	-	2/6 (33.3; 9.7-70)	1/6 (16.7; 3-56.4)	2/6 (33.3; 9.7-70)
COVID-19	16A							
Pre-COVID-19	16F	12/13 (92.3; 66.7-98.6)	6/13 (46.2; 23.2-70.9)	7/13 (53.8; 29.1-76.8)	6/13 (46.2; 23.2-70.9)	1/13 (7.7; 1.4-33.3)	-	-
COVID-19	16F	15/16 (93.8; 71.7-98.9)	12/16 (75; 50.5-89.8)	4/16 (25; 10.2-49.5)	3/16 (18.8; 6.6-43)	-	-	1/16 (6.2; 1.1-28.3)
Pre-COVID-19	17F	7/17 (41.2; 21.6-64)	4/17 (23.5; 9.6-47.3)	13/17 (76.5; 52.7-90.4)	3/17 (17.6; 6.2-41)	4/17 (23.5; 9.6-47.3)	2/17 (11.8; 3.3-34.3)	4/17 (23.5; 9.6-47.3)
COVID-19	17F	2/2 (100; 34.2-100)	2/2 (100; 34.2-100)	-	-	-	-	-
Pre-COVID-19	18B	1/1 (100; 20.7-100)	1/1 (100; 20.7-100)	-	-	-	-	-
COVID-19	18B							
Pre-COVID-19	19B	2/4 (50; 15-85)	-	4/4 (100; 51-100)	2/4 (50; 15-85)	1/4 (25; 4.6-69.9)	1/4 (25; 4.6-69.9)	-
COVID-19	19B	2/2 (100; 34.2-100)	2/2 (100; 34.2-100)	-	-	-	-	-
Pre-COVID-19	20	3/4 (75; 30.1-95.4)	1/4 (25; 4.6-69.9)	3/4 (75; 30.1-95.4)	2/4 (50; 15-85)	1/4 (25; 4.6-69.9)	-	-
COVID-19	20	2/2 (100; 34.2-100)	2/2 (100; 34.2-100)	-	-	-	-	-
Pre-COVID-19	21	6/13 (46.2; 23.2-70.9)	4/13 (30.8; 12.7-57.6)	9/13 (69.2; 42.4-87.3)	2/13 (15.4; 4.3-42.2)	5/13 (38.5; 17.7-64.5)	1/13 (7.7; 1.4-33.3)	1/13 (7.7; 1.4-33.3)
COVID-19	21	15/15 (100; 79.6-100)	13/15 (86.7; 62.1-96.3)	2/15 (13.3; 3.7-37.9)	2/15 (13.3; 3.7-37.9)	-	-	-
Pre-COVID-19	22A	-	-	2/2 (100; 34.2-100)	-	2/2 (100; 34.2-100)	-	-
COVID-19	22A							
Pre-COVID-19	22F	1/1 (100; 20.7-100)	1/1 (100; 20.7-100)	-	-	-	-	-
COVID-19	22F	2/2 (100; 34.2-100)	2/2 (100; 34.2-100)	-	-	-	-	-
Pre-COVID-19	23A	7/10 (70; 39.7-89.2)	4/10 (40; 16.8-68.7)	6/10 (60; 31.3-83.2)	3/10 (30; 10.8-60.3)	3/10 (30; 10.8-60.3)	-	-

COVID-19	23A	6/7 (85.7; 48.7-97.4)	5/7 (71.4; 35.9-91.8)	2/7 (28.6; 8.2-64.1)	1/7 (14.3; 2.6-51.3)	1/7 (14.3; 2.6-51.3)	-	-
Pre-COVID-19	23B	20/21 (95.2; 77.3-99.2)	11/21 (52.4; 32.4-71.7)	10/21 (47.6; 28.3-67.6)	9/21 (42.9; 24.5-63.5)	1/21 (4.8; 0.8-22.7)	-	-
COVID-19	23B	10/10 (100; 72.2-100)	5/10 (50; 23.7-76.3)	5/10 (50; 23.7-76.3)	5/10 (50; 23.7-76.3)	-	-	-
Pre-COVID-19	24A	-	-	2/2 (100; 34.2-100)	-	1/2 (50; 9.5-90.5)	-	1/2 (50; 9.5-90.5)
COVID-19	24A							
Pre-COVID-19	24BF	1/5 (20; 3.6-62.4)	-	5/5 (100; 56.6-100)	1/5 (20; 3.6-62.4)	-	2/5 (40; 11.8-76.9)	2/5 (40; 11.8-76.9)
COVID-19	24BF							
Pre-COVID-19	27	-	-	4/4 (100; 51-100)	-	-	1/4 (25; 4.6-69.9)	3/4 (75; 30.1-95.4)
COVID-19	27							
Pre-COVID-19	29	-	-	8/8 (100; 67.6-100)	-	5/8 (62.5; 30.6-86.3)	1/8 (12.5; 2.2-47.1)	2/8 (25; 7.1-59.1)
COVID-19	29							
Pre-COVID-19	31	1/6 (16.7; 3-56.4)	-	6/6 (100; 61-100)	1/6 (16.7; 3-56.4)	-	2/6 (33.3; 9.7-70)	3/6 (50; 18.8-81.2)
COVID-19	31							
Pre-COVID-19	32AF	-	-	1/1 (100; 20.7-100)	-	-	-	1/1 (100; 20.7-100)
COVID-19	33AF	-	-	3/3 (100; 43.9-100)	-	-	1/3 (33.3; 6.1-79.2)	2/3 (66.7; 20.8-93.9)
Pre-COVID-19	33B	2/8 (25; 7.1-59.1)	1/8 (12.5; 2.2-47.1)	7/8 (87.5; 52.9-97.8)	1/8 (12.5; 2.2-47.1)	3/8 (37.5; 13.7-69.4)	1/8 (12.5; 2.2-47.1)	2/8 (25; 7.1-59.1)
COVID-19	33B							
Pre-COVID-19	33C	-	-	5/5 (100; 56.6-100)	-	2/5 (40; 11.8-76.9)	1/5 (20; 3.6-62.4)	2/5 (40; 11.8-76.9)
COVID-19	33C							
Pre-COVID-19	34	11/14 (78.6; 52.4-92.4)	10/14 (71.4; 45.4-88.3)	4/14 (28.6; 11.7-54.6)	1/14 (7.1; 1.3-31.5)	2/14 (14.3; 4-39.9)	1/14 (7.1; 1.3-31.5)	-
COVID-19	34	7/7 (100; 64.6-100)	7/7 (100; 64.6-100)	-	-	-	-	-
Pre-COVID-19	35AC42	-	-	8/8 (100; 67.6-100)	-	4/8 (50; 21.5-78.5)	3/8 (37.5; 13.7-69.4)	1/8 (12.5; 2.2-47.1)
COVID-19	35AC42	2/8 (25; 7.1-59.1)	2/8 (25; 7.1-59.1)	6/8 (75; 40.9-92.9)	-	5/8 (62.5; 30.6-86.3)	1/8 (12.5; 2.2-47.1)	-
Pre-COVID-19	35B	11/13 (84.6; 57.8-95.7)	5/13 (38.5; 17.7-64.5)	8/13 (61.5; 35.5-82.3)	6/13 (46.2; 23.2-70.9)	1/13 (7.7; 1.4-33.3)	-	1/13 (7.7; 1.4-33.3)
COVID-19	35B	7/7 (100; 64.6-100)	5/7 (71.4; 35.9-91.8)	2/7 (28.6; 8.2-64.1)	2/7 (28.6; 8.2-64.1)	-	-	-
Pre-COVID-19	35F	4/6 (66.7; 30-90.3)	3/6 (50; 18.8-81.2)	3/6 (50; 18.8-81.2)	1/6 (16.7; 3-56.4)	2/6 (33.3; 9.7-70)	-	-
COVID-19	35F	-	-	1/1 (100; 20.7-100)	-	1/1 (100; 20.7-100)	-	-
Pre-COVID-19	36	-	-	3/3 (100; 43.9-100)	-	1/3 (33.3; 6.1-79.2)	-	2/3 (66.7; 20.8-93.9)
COVID-19	36							
Pre-COVID-19	37							
COVID-19	37	1/2 (50; 9.5-90.5)	1/2 (50; 9.5-90.5)	1/2 (50; 9.5-90.5)	-	1/2 (50; 9.5-90.5)	-	-
Pre-COVID-19	41A	1/2 (50; 9.5-90.5)	1/2 (50; 9.5-90.5)	1/2 (50; 9.5-90.5)	-	-	-	1/2 (50; 9.5-90.5)
COVID-19	41A							
Pre-COVID-19	43	2/3 (66.7; 20.8-93.9)	-	3/3 (100; 43.9-100)	2/3 (66.7; 20.8-93.9)	1/3 (33.3; 6.1-79.2)	-	-
COVID-19	43	1/3 (33.3; 6.1-79.2)	-	3/3 (100; 43.9-100)	1/3 (33.3; 6.1-79.2)	1/3 (33.3; 6.1-79.2)	1/3 (33.3; 6.1-79.2)	-
Pre-COVID-19	45	-	-	12/12 (100; 75.8-100)	-	5/12 (41.7; 19.3-68)	4/12 (33.3; 13.8-60.9)	3/12 (25; 8.9-53.2)
COVID-19	45	-	-	4/4 (100; 51-100)	-	3/4 (75; 30.1-95.4)	1/4 (25; 4.6-69.9)	-
Pre-COVID-19	46	-	-	2/2 (100; 34.2-100)	-	-	1/2 (50; 9.5-90.5)	1/2 (50; 9.5-90.5)
COVID-19	46							
Pre-COVID-19	47A	2/11 (18.2; 5.1-47.7)	1/11 (9.1; 1.6-37.7)	10/11 (90.9; 62.3-98.4)	1/11 (9.1; 1.6-37.7)	4/11 (36.4; 15.2-64.6)	2/11 (18.2; 5.1-47.7)	3/11 (27.3; 9.7-56.6)
COVID-19	47A	-	-	1/1 (100; 20.7-100)	-	1/1 (100; 20.7-100)	-	-
Pre-COVID-19	48	-	-	5/5 (100; 56.6-100)	-	1/5 (20; 3.6-62.4)	1/5 (20; 3.6-62.4)	3/5 (60; 23.1-88.2)
COVID-19	48							

† n is the number of samples in each category for each period and N is the total number of number of samples identified as each serotype/group. The rank was determined according to the colonisation density. a. Primary coloniser - prevalence of either a single coloniser OR a dominant coloniser in the case of multiple colonisation and includes both single coloniser and first serotype in the case of multiple colonisation. b. Single serotype - serotypes which ae not found with other serotypes. c. Concurrently carried - prevalence of a given serotype given other serotypes are present. d. First serotype – includes those that were first serotypes (highest density) of those that are concurrently carried with multiple other serotypes. e. Second serotype - includes serotypes that were second serotypes (second highest density) of those that are concurrently carried with multiple other serotypes. f. Third serotype - includes serotypes that were third serotypes (third highest density) of those that are concurrently carried with multiple other serotypes. g. Fourth serotype - includes serotypes ≥four (≥fourth highest density) of those that are concurrently carried with multiple other serotypes.

Supplementary Table 14: Prevalence of bacterial colonisers

**All age groups combined**					
	**Pre-Covid-19 era (2018), N=571 n (%; 95% CI)**	**Covid-19 era (2021), N=536 n (%; 95% CI)**	**p-value ***	**OR (95% CI); p-value**	**AOR (95% CI); p-value**
A. baumannii	34 (5.95; 4.29-8.21)	102 (19.03; 15.93-22.57)	<0.0001	3.71 (2.44-5.76); p=<0.0001	1.55 (1.35-1.77); p<0.0001
B. holmesii	1 (0.18; 0.03-0.99)	0 (0; 0-0.71)	>0.99	-	-
B. parapertussis/bronchiseptica	1 (0.18; 0.03-0.99)	0 (0; 0-0.71)	>0.99	-	-
B. pertussis	-	-	-	-	-
H. influenzae	4 (0.7; 0.27-1.79)	3 (0.56; 0.19-1.63)	>0.99	0.8 (0.12-4.74); p=>0.99	0.95 (0.57-1.56); p=0.83
H. influenzae-b	-	-	-	-	-
Non-typeable H. influenzae	280 (49.04; 44.96-53.13)	165 (30.78; 27.02-34.82)	<0.0001	0.46 (0.36-0.60); p=<0.0001	0.77 (0.71-0.84); p<0.0001
K. pneumoniae	81 (14.19; 11.56-17.29)	70 (13.06; 10.47-16.18)	0.6	0.91 (0.63-1.30); p=0.6	0.97 (0.87-1.09); p=0.66
M. catarrhalis	328 (57.44; 53.35-61.44)	242 (45.15; 40.99-49.38)	<0.0001	0.61 (0.48-0.78); p=<0.0001	0.85 (0.79-0.92); p=0.0001
N. Lactamica	51 (8.93; 6.86-11.55)	13 (2.43; 1.42-4.11)	<0.0001	0.25 (0.13-0.48); p=<0.0001	0.64 (0.52-0.78); p<0.0001
N. meningitidis	3 (0.53; 0.18-1.53)	0 (0; 0-0.71)	0.25	-	-
S. aureus	29 (5.08; 3.56-7.20)	52 (9.7; 7.48-12.5)	0.0037	2.01 (1.23-3.34); p=0.0037	1.28 (1.10-1.50); p=0.0019
S. oralis	99 (17.34; 14.45-20.66)	11 (2.05; 1.15-3.64)	<0.0001	0.1 (0.05-0.19); p=<0.0001	0.46 (0.37-0.57); p<0.0001
S. pyrogenes	7 (1.23; 0.6-2.51)	3 (0.56; 0.19-1.63)	0.34	0.45 (0.08-2.0); p=0.34	0.77 (0.49-1.21); p=0.26
**≤24 months of age**					
	**Pre-Covid-19 era (2018), N=289 n (%; 95% CI)**	**Covid-19 era (2021), N=267 n (%; 95% CI)**	**p-value ***	**OR (95% CI); p-value**	**AOR (95% CI); p-value**
A. baumannii	19 (6.57; 4.25-10.04)	43 (16.1; 12.18-20.99)	0.00041	2.72 (1.50-5.10); p=0.00041	1.38 (1.14-1.68); p=0.0009
B. holmesii	-	-	-	-	-
B. parapertussis/bronchiseptica	-	-	-	-	-
B. pertussis	-	-	-	-	-
H. influenzae	1 (0.35; 0.06-1.93)	2 (0.75; 0.21-2.69)	0.61	2.17 (0.11-128.58); p=0.61	1.34 (0.6-2.99); p=0.48
H. influenzae-b	-	-	-	-	-
Non-typeable H. influenzae	135 (46.71; 41.04-52.47)	76 (28.46; 23.39-34.15)	<0.0001	0.45 (0.31-0.66); p=<0.0001	0.77 (0.68-0.86); p<0.0001
K. pneumoniae	39 (13.49; 10.03-17.92)	38 (14.23; 10.55-18.93)	0.81	1.06 (0.64-1.77); p=0.807	1.02 (0.86-1.20); p=0.83
M. catarrhalis	173 (59.86; 54.12-65.35)	116 (43.45; 37.63-49.44)	0.00013	0.52 (0.36-0.73); p=0.000129	0.8 (0.71-0.90); p=0.0001
N. Lactamica	27 (9.34; 6.5-13.25)	7 (2.62; 1.28-5.31)	0.0011	0.26 (0.09-0.63); p=0.00113	0.64 (0.48-0.85); p=0.0024
N. meningitidis	3 (1.04; 0.35-3.01)	0 (0; 0-1.42)	0.25	-	-
S. aureus	19 (6.57; 4.25-10.04)	24 (8.99; 6.11-13.03)	0.34	1.4 (0.72-2.78); p=0.341	1.15 (0.93-1.42); p=0.19
S. oralis	38 (13.15; 9.73-17.53)	6 (2.25; 1.03-4.82)	<0.0001	0.15 (0.05-0.37); p=<0.0001	0.52 (0.39-0.71); p<0.0001
S. pyrogenes	2 (0.69; 0.19-2.49)	2 (0.75; 0.21-2.69)	>0.99	1.08 (0.08-15.03); p=>0.9999	1.04 (0.54-2.01); p=0.90
**>24 months of age**					
	**Pre-Covid-19 era (2018), N=282 n (%; 95% CI)**	**Covid-19 era (2021), N=269 n (%; 95% CI)**	**p-value ***	**OR (95% CI); p-value**	**AOR (95% CI); p-value**
A. baumannii	15 (5.32; 3.25-8.59)	59 (21.93; 17.4-27.25)	<0.0001	4.99 (2.70-9.75); p=<0.0001	1.72 (1.41-2.10); p<0.0001
B. holmesii	1 (0.35; 0.06-1.98)	0 (0; 0-1.41)	>0.99	-	-
B. parapertussis/bronchiseptica	1 (0.35; 0.06-1.98)	0 (0; 0-1.41)	>0.99	-	-

B. pertussis	-	-	-	-	-
H. influenzae	3 (1.06; 0.36-3.08)	1 (0.37; 0.07-2.08)	0.62	0.35 (0.01-4.36); p=0.62	0.72 (0.34-1.53); p=0.39
H. influenzae-b	-	-	-	-	-
Non-typeable H. influenzae	145 (51.42; 45.61-57.19)	89 (33.09; 27.74-38.91)	<0.0001	0.47 (0.33-0.67); p=<0.0001	0.78 (0.69-0.88); p<0.0001
K. pneumoniae	42 (14.89; 11.21-19.52)	32 (11.9; 8.55-16.31)	0.32	0.77 (0.45-1.30); p=0.32	0.92 (0.78-1.09); p=0.34
M. catarrhalis	155 (54.96; 49.13-60.67)	126 (46.84; 40.96-52.81)	0.06	0.72 (0.51-1.02); p=0.061	0.91 (0.81-1.02); p=0.092
N. Lactamica	24 (8.51; 5.79-12.35)	6 (2.23; 1.03-4.78)	0.0012	0.25 (0.08-0.63); p=0.0012	0.63 (0.46-0.85); p=0.0028
N. meningitidis	-	-	-	-	-
S. aureus	10 (3.55; 1.94-6.4)	28 (10.41; 7.3-14.63)	0.0021	3.15 (1.45-7.43); p=0.0021	1.48 (1.15-1.89); p=0.0021
S. oralis	61 (21.63; 17.22-26.80)	5 (1.86; 0.80-4.28)	<0.0001	0.07 (0.02-0.17); p=<0.0001	0.41 (0.3-0.56); p<0.0001
S. pyrogenes	5 (1.77; 0.76-4.08)	1 (0.37; 0.07-2.08)	0.22	0.21 (0-1.87); p=0.22	0.59 (0.29-1.21); p=0.15

aOR. adjusted odd ratio and 95% CI calculated using logistic regression analyse. When odd ratios (OR) could not be calculated, Fishers exact test was used to determine significance.

Supplementary Table 15: Bacterial carriage density from Nasopharyngeal Swab samples collected from children 0-60 months of age in the Pre-COVID-19 period (2018, N=572) and COVID-19 period (2021, N=536)

		All ages			<24 months of age			>24 months of age		
**Bacteria**	**Study period**	**n***	**Mean Log_10 GE/ml (95%CI)**	**p-value**	**n***	**Mean Log_10 GE/ml (95%CI)**	**p-value**	**n***	**Mean Log_10 GE/ml (95%CI)**	**p-value**
A. baumannii	Pre-COVID-19	34	1.56 (1.65-1.46)		19	1.56 (1.68-1.43)		15	1.56 (1.73-1.39)	
A. baumannii	COVID-19	102	1.61 (1.68-1.55)	0.26	43	1.7 (1.82-1.58)	0.18	59	1.55 (1.63-1.48)	0.95
B. holmesii	Pre-COVID-19	1	1; 5.62		0	-		1	1; 5.62	
B. holmesii	COVID-19	0	-	-	0	-	-	0	-	-
B. parapertussis/bronchiseptica	Pre-COVID-19	1	1; 5.34		0	-		1	1; 5.34	
B. parapertussis/bronchiseptica	COVID-19	0	-	-	0	-	-	0	-	-
B. pertussis	Pre-COVID-19	0	-		0	-		0	-	
B. pertussis	COVID-19	0	-	-	0	-	-	0	-	-
H. influenzae	Pre-COVID-19	4	4.88 (5.88-3.88)		1	3.5		3	5.34 (5.62-5.07)	
H. influenzae	COVID-19	3	3.94 (5.32-2.56)	0.81	2	3.83 (6.76-0.91)	-	1	4.14	-
H. influenzae-b	Pre-COVID-19	0	-		0	-		0	-	
H. influenzae-b	COVID-19	0	-	-	0	-	-	0	-	-
Non-typeable H. influenzae	Pre-COVID-19	280	3.2 (3.32-3.08)		135	3.32 (3.49-3.15)		145	3.09 (3.25-2.92)	
Non-typeable H. influenzae	COVID-19	165	2.79 (2.93-2.65)	0.00043	76	2.7 (2.91-2.49)	0.00024	89	2.86 (3.06-2.67)	0.12
K. pneumoniae	Pre-COVID-19	81	2.27 (2.42-2.12)		39	2.24 (2.47-2.01)		42	2.3 (2.51-2.09)	
K. pneumoniae	COVID-19	70	1.53 (1.61-1.45)	<0.0001	38	1.54 (1.65-1.43)	<0.0001	32	1.51 (1.62-1.4)	<0.0001
M. catarrhalis	Pre-COVID-19	328	4.43 (4.54-4.32)		173	4.57 (4.71-4.43)		155	4.28 (4.44-4.11)	
M. catarrhalis	COVID-19	242	3.81 (3.94-3.68)	<0.0001	116	3.95 (4.15-3.76)	<0.0001	126	3.68 (3.85-3.50)	<0.0001
N. Lactamica	Pre-COVID-19	51	2.33 (2.51-2.16)		27	2.43 (2.66-2.2)		24	2.22 (2.49-1.95)	
N. Lactamica	COVID-19	13	2.17 (2.56-1.77)	0.51	7	2.53 (3.19-1.87)	0.92	6	1.75 (2.06-1.44)	0.36
N. meningitidis	Pre-COVID-19	3	3; 2.51 (3.35-1.67)		3	3; 2.51 (3.35-1.67)		0	-	
N. meningitidis	COVID-19	0	-	-	0	-	-	0	-	-
S. aureus	Pre-COVID-19	29	3.15 (3.52-2.79)		19	3.16 (3.65-2.68)		10	3.13 (3.75-2.5)	
S. aureus	COVID-19	52	2.78 (3.0-2.56)	0.16	24	2.86 (3.20-2.53)	0.42	28	2.71 (3.01-2.4)	0.24
S. pneumoniae	Pre-COVID-19	282	4.72 (4.8-4.63)		147	4.84 (4.96-4.72)		135	4.58 (4.7-4.46)	
S. pneumoniae	COVID-19	274	3.96 (4.07-3.85)	<0.0001	135	4.06 (4.22-3.9)	<0.0001	139	3.87 (4.01-3.72)	<0.0001
S. oralis	Pre-COVID-19	99	2.45 (2.55-2.35)		38	2.37 (2.54-2.21)		61	2.5 (2.63-2.38)	
S. oralis	COVID-19	11	2.19 (2.49-1.88)	0.3	6	2.14 (2.70-1.59)	0.67	5	2.24 (2.62-1.85)	0.42
S. pyrogenes	Pre-COVID-19	7	1.66 (2.11-1.2)		2	2.08 (4.60-0.43)		5	1.48 (1.76-1.20)	
S. pyrogenes	COVID-19	3	3.4 (7.20-0.4)	0.11	2	1.79 (2.28-1.30)	0.95	1	6.63	-

n is the number of samples. Density of carriage was determined through quantitative real-time nanofluidic PCR in the Fluidigm.

Supplementary Table 16: Concurrent bacterial colonisation

	Pre-COVID-19	COVID-19	aOR (95%CI); p-value
**Not colonised**	76/571 (13.3; 10.8-16.3)	103/536 (19.2; 16.1-22.8)	0.87 (0.79-0.96); 0.0064
**Colonised**	495/571 (86.7; 83.7-89.2)	433/536 (80.8; 77.2-83.9)	
**1 bacteria**	125/495 (25.3; 21.6-29.3)	144/433 (33.3; 29-37.8)	0.0091.14 (1.04-1.26); 0.0064
**2 bacteria**	158/495 (31.9; 28-36.2)	143/433 (33; 28.8-37.6)	1.01 (0.92-1.11); 0.85
**3 bacteria**	120/495 (24.2; 20.7-28.2)	93/433 (21.5; 17.9-25.6)	0.95 (0.86-1.06); 0.38
**>=4 bacteria**	92/495 (18.6; 15.4-22.2)	53/433 (12.2; 9.5-15.7)	0.85 (0.75-0.96); 0.0092

Supplementary Table 17: Multiple concurrent bacterial colonisation detected in Nasopharyngeal Swab samples collected from children 0-60 months of age in Pre-COVID period (2018, N=572) and COVID-19 period (2021, N=536)

Study period	Bacteria	Primary bacteria (a) n/N (%; 95%CI)	Single bacteria (b) n/N (%; 95%CI)	Concurrently carried (c) n/N (%; 95%CI)	First bacteria (d) n/N (%; 95%CI)	Second bacteria (e) n/N (%; 95%CI)	Third bacteria (f) n/N (%; 95%CI)	≥ Four bacteria (g) n/N (%; 95%CI)
Pre-COVID-19	*A.baumannii*	4/34 (11.8; 4.7-26.6)	4/34 (11.8; 4.7-26.6)	30/34 (88.2; 73.4-95.3)	-	10/34 (29.4; 16.8-46.2)	4/34 (11.8; 4.7-26.6)	16/34 (47.1; 31.5-63.3)
COVID-19	*A.baumannii*	13/102 (12.7; 7.6-20.6)	9/102 (8.8; 4.7-15.9)	93/102 (91.2; 84.1-95.3)	4/102 (3.9; 1.5-9.7)	28/102 (27.5; 19.7-36.8)	30/102 (29.4; 21.4-38.9)	31/102 (30.4; 22.3-39.9)
Pre-COVID-19	*B. holmesii*	1/1 (100; 20.7-100)	1/1 (100; 20.7-100)	-	-	-	-	-
COVID-19	*B. holmesii*							
Pre-COVID-19	*B. parapertusis*	1/1 (100; 20.7-100)	-	1/1 (100; 20.7-100)	1/1 (100; 20.7-100)	-	-	-
COVID-19	*B. parapertusis*							
Pre-COVID-19	*H. influenzae*	1/4 (25; 4.6-69.9)	-	4/4 (100; 51-100)	1/4 (25; 4.6-69.9)	3/4 (75; 30.1-95.4)	-	-
COVID-19	*H. influenzae*	-	-	3/3 (100; 43.9-100)	-	3/3 (100; 43.9-100)	-	-
Pre-COVID-19	*Non typeable H. influenzae*	64/280 (22.9; 18.3-28.1)	28/280 (10; 7-14.1)	252/280 (90; 85.9-93)	36/280 (12.9; 9.4-17.3)	101/280 (36.1; 30.7-41.8)	95/280 (33.9; 28.6-39.7)	20/280 (7.1; 4.7-10.8)
COVID-19	*Non typeable H. influenzae*	40/165 (24.2; 18.3-31.3)	17/165 (10.3; 6.5-15.9)	148/165 (89.7; 84.1-93.5)	23/165 (13.9; 9.5-20)	52/165 (31.5; 24.9-39)	66/165 (40; 32.8-47.6)	7/165 (4.2; 2.1-8.5)
Pre-COVID-19	*K. pneumoniae*	18/81 (22.2; 14.5-32.4)	9/81 (11.1; 6-19.8)	72/81 (88.9; 80.2-94)	9/81 (11.1; 6-19.8)	25/81 (30.9; 21.9-41.6)	17/81 (21; 13.5-31.1)	21/81 (25.9; 17.6-36.4)
COVID-19	*K. pneumoniae*	17/70 (24.3; 15.8-35.5)	13/70 (18.6; 11.2-29.2)	57/70 (81.4; 70.8-88.8)	4/70 (5.7; 2.2-13.8)	20/70 (28.6; 19.3-40.1)	19/70 (27.1; 18.1-38.5)	14/70 (20; 12.3-30.8)
Pre-COVID-19	*M. catarrhalis*	195/328 (59.5; 54.1-64.6)	45/328 (13.7; 10.4-17.9)	283/328 (86.3; 82.1-89.6)	150/328 (45.7; 40.4-51.1)	96/328 (29.3; 24.6-34.4)	34/328 (10.4; 7.5-14.1)	3/328 (0.9; 0.3-2.7)
COVID-19	*M. catarrhalis*	134/242 (55.4; 49.1-61.5)	33/242 (13.6; 9.9-18.5)	209/242 (86.4; 81.5-90.1)	101/242 (41.7; 35.7-48)	96/242 (39.7; 33.7-45.9)	11/242 (4.5; 2.6-8)	1/242 (0.4; 0.1-2.3)
Pre-COVID-19	*N. Lactamica*	3/51 (5.9; 2-15.9)	2/51 (3.9; 1.1-13.2)	49/51 (96.1; 86.8-98.9)	1/51 (2; 0.3-10.3)	8/51 (15.7; 8.2-28)	18/51 (35.3; 23.6-49)	22/51 (43.1; 30.5-56.7)
COVID-19	*N. Lactamica*	1/13 (7.7; 1.4-33.3)	1/13 (7.7; 1.4-33.3)	12/13 (92.3; 66.7-98.6)	-	1/13 (7.7; 1.4-33.3)	4/13 (30.8; 12.7-57.6)	7/13 (53.8; 29.1-76.8)
Pre-COVID-19	*N. meningitidis*	-	-	3/3 (100; 43.9-100)	-	-	2/3 (66.7; 20.8-93.9)	1/3 (33.3; 6.1-79.2)
COVID-19	*N. meningitidis*							
Pre-COVID-19	*S. aureus*	7/29 (24.1; 12.2-42.1)	4/29 (13.8; 5.5-30.6)	25/29 (86.2; 69.4-94.5)	3/29 (10.3; 3.6-26.4)	8/29 (27.6; 14.7-45.7)	7/29 (24.1; 12.2-42.1)	7/29 (24.1; 12.2-42.1)
COVID-19	*S. aureus*	32/52 (61.5; 48-73.5)	19/52 (36.5; 24.8-50.1)	33/52 (63.5; 49.9-75.2)	13/52 (25; 15.2-38.2)	11/52 (21.2; 12.2-34)	4/52 (7.7; 3-18.2)	5/52 (9.6; 4.2-20.6)
Pre-COVID-19	*S. pneumonaie*	21/99 (21.2; 14.3-30.3)	11/99 (11.1; 6.3-18.8)	88/99 (88.9; 81.2-93.7)	10/99 (10.1; 5.6-17.6)	26/99 (26.3; 18.6-35.7)	23/99 (23.2; 16-32.5)	29/99 (29.3; 21.2-38.9)
COVID-19	*S. pneumonaie*	4/11 (36.4; 15.2-64.6)	2/11 (18.2; 5.1-47.7)	9/11 (81.8; 52.3-94.9)	2/11 (18.2; 5.1-47.7)	1/11 (9.1; 1.6-37.7)	5/11 (45.5; 21.3-72)	1/11 (9.1; 1.6-37.7)
Pre-COVID-19	*S. oralis*	180/282 (63.8; 58.1-69.2)	21/282 (7.4; 4.9-11.1)	261/282 (92.6; 88.9-95.1)	159/282 (56.4; 50.5-62)	91/282 (32.3; 27.1-37.9)	10/282 (3.5; 1.9-6.4)	1/282 (0.4; 0.1-2)
COVID-19	*S. oralis*	191/274 (69.7; 64-74.8)	49/274 (17.9; 13.8-22.9)	225/274 (82.1; 77.1-86.2)	142/274 (51.8; 45.9-57.7)	77/274 (28.1; 23.1-33.7)	6/274 (2.2; 1-4.7)	-
Pre-COVID-19	*S. pyrogenes*	-	-	7/7 (100; 64.6-100)	-	2/7 (28.6; 8.2-64.1)	2/7 (28.6; 8.2-64.1)	3/7 (42.9; 15.8-75)
COVID-19	*S. pyrogenes*	1/3 (33.3; 6.1-79.2)	1/3 (33.3; 6.1-79.2)	2/3 (66.7; 20.8-93.9)	-	-	1/3 (33.3; 6.1-79.2)	1/3 (33.3; 6.1-79.2)

n - is the number of samples in each category for each period and N is the total number of number of samples identified as each serotype/group. The rank was determined according to the colonisation density. a. Primary bacteria - prevalence of either a single coloniser OR a dominant coloniser in the case of multiple colonisation and includes both single coloniser and first bacteria in the case of multiple colonisation. b. Single bacteria - bacteria which ae not found with other bacteria. c. Concurrently carried - prevalence of a given bacteria given other bacteria are present. d. First bacteria – includes those that were first bacteria (highest density) of those that are concurrently carried with multiple other bacteria. e. Second bacteria - includes bacteria that were the second bacteria (second highest density) detected of those that are concurrently carried with multiple other bacteria. f. Third bacteria – includes bacteria that were third bacteria (third highest density) detected of those that are concurrently carried with multiple other bacteria. g. ≥Fourth bacteria - includes bacteria that were ≥four (≥fourth highest density) detected of those that are concurrently carried with multiple other bacteria.

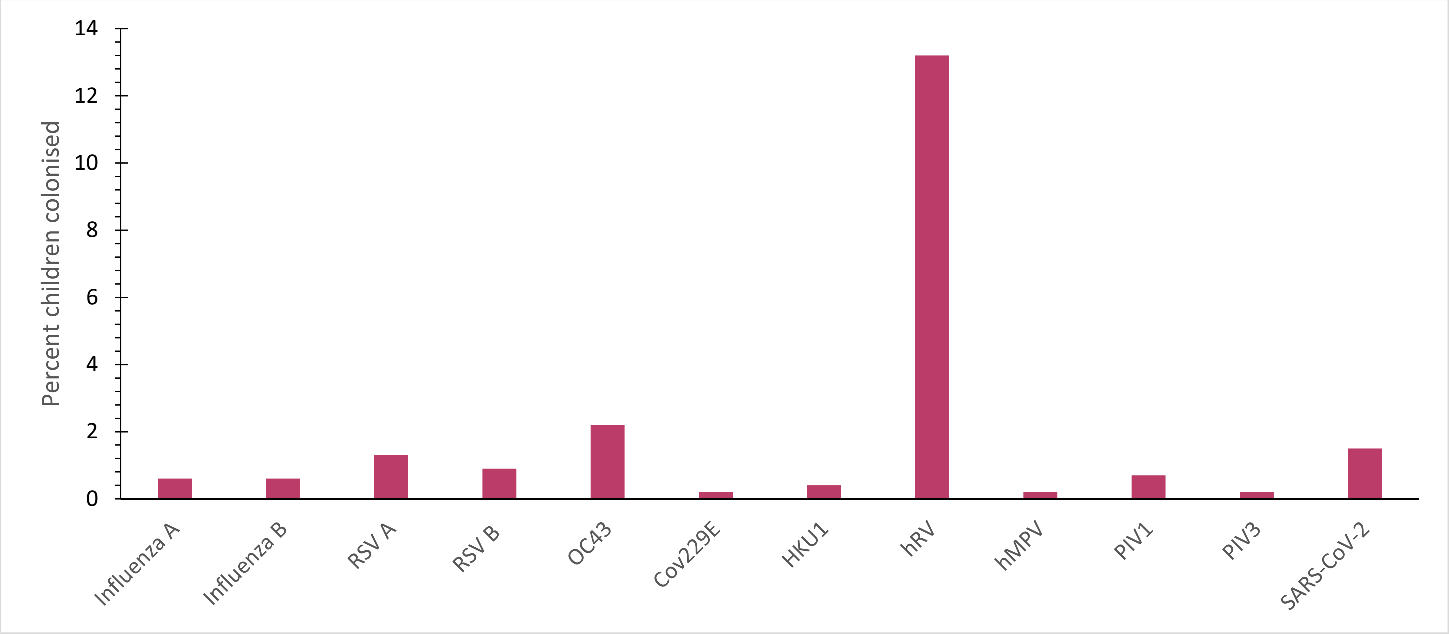

Supplementary Fig 4: Identification of viruses in children 0-60 months of age in the Covid-19 era (2021; n=356)

CoV-NL63 was not detected

Supplementary Table 18: Viral density in Nasopharyngeal Swab samples collected from children 0-60 months of age in the Covid-19 era (2021. n=536).

-	All Ages		<24 months of age		>24 months of age	
	n*	Mean Log_{10} GE/ml (95%CI)	n*	Mean Log_{10} GE/ml (95%CI)	n*	Mean Log_{10} GE/ml (95%CI)
Cov229E	1	1; 2.07	1	1; 2.07	0	-
HKU1	2	2; 2.62 (4.38-0.85)	1	1; 3.22	1	1; 2.01
HMPV	1	1; 3.15	1	1; 3.15	0	-
HRV	71	71; 4.06 (4.25-3.87)	37	37; 4.15 (4.39-3.91)	34	34; 3.96 (4.25-3.66)
INFA	3	3; 5.27 (6.25-4.30)	2	2; 5.61 (6.82-4.41)	1	1; 4.6
INFB	3	3; 2.78 (3.73-1.84)	2	2; 2.46 (3.64-1.28)	1	1; 3.43
OC43	12	12; 3.15 (3.85-2.45)	8	8; 3.08 (4.16-1.99)	4	4; 3.3 (3.99-2.60)
PIV1	4	4; 3.74 (5.14-2.34)	3	3; 3.5 (5.53-1.46)	1	1; 4.47
PIV3	1	1; 4.6	1	1; 4.6	0	-
RSVA	7	7; 3.53 (4.15-2.91)	3	3; 3.7 (3.85-3.55)	4	4; 3.41 (4.69-2.12)
RSVB	5	5; 3.33 (4.22-2.43)	4	4; 2.96 (3.66-2.27)	1	1; 4.79

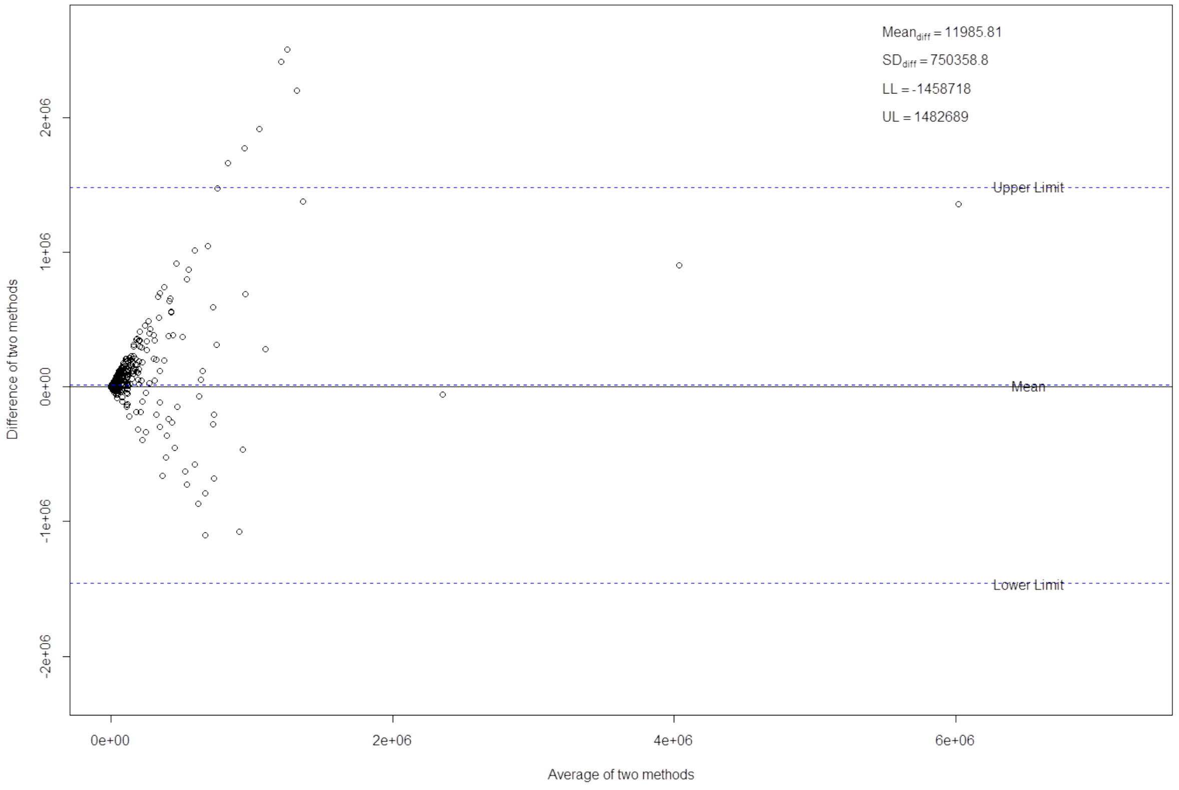

Supplementary Fig 5: Comparison between the density of serotypes and the pneumococcal reference genes

REFERENCES

1. Downs SL, Madhi SA, van Der Merwe L, Nunes MC, Olwagen CP. High-throughput nanofluidic real-time PCR to discriminate Pneumococcal Conjugate Vaccine (PCV)-associated serogroups 6, 18, and 22 to serotypes using modified oligonucleotides. Scientific reports. 2021;11(1):1-11.
